# Metabolomic analyses reveal that graphene oxide alleviates nicosulfuron toxicity in sweet corn

**DOI:** 10.3389/fpls.2025.1529598

**Published:** 2025-02-25

**Authors:** Jian Wang, Yanbing Wang, Yanli Wang, Xuemei Zhong, Xiuping Wang, Xiaohu Lin

**Affiliations:** ^1^ College of Agronomy and Biotechnology, Hebei Key Laboratory of Crop Stress Biology, Hebei Normal University of Science and Technology, Qinhuangdao, China; ^2^ Institute of Cereal and Oil Crops, Hebei Key Laboratory of Crop Genetics and Breeding, Hebei Academy of Agriculture and Forestry Sciences, Shijiazhuang, China; ^3^ College of Agronomy, Shenyang Agricultural University, Shenyang, China

**Keywords:** flavonoid, graphene oxide, metabolomics, nicosulfuron, sweet corn

## Abstract

Nicosulfuron can repress the growth and quality of sweet corn (*Zea mays*), and graphene oxide has been used for sustainable agriculture. However, the underlying mechanism of the toxicity of nicosulfuron that is mediated in sweet corn remains elusive. To explore the potential mechanism of GO-mediated nicosulfuron toxicity in sweet corn in this study, we investigated the effects of graphene oxide on nicosulfuron stress in the sweet corn sister inbred lines of H01 and H20. Furthermore, we performed a metabolomics analysis for the H01 and H20 under different treatments. The results showed that nicosulfuron severely affected the rate of survival, physiological parameters, photosynthetic indicators, and chlorophyll fluorescence parameters of corn seedlings, whereas foliar spraying with graphene oxide promoted the rate of survival under nicosulfuron toxicity. The metabolomics analysis showed that 70 and 90 metabolites differentially accumulated in the H01 and H20 inbred lines under nicosulfuron treatment, respectively. Graphene oxide restored 59 metabolites in the H01 seedlings and 56 metabolites to normal levels in the H20 seedlings, thereby promoting the rate of survival of the sweet corn seedlings. Compared with nicosulfuron treatment alone, graphene oxide resulted in 108 and 66 differential metabolites in the H01 and H20 inbred lines, respectively. A correlation analysis revealed that metabolites, such as doronine and (R)-2-hydroxy-2-hydroxylase-1,4-benzoxazin-3(4-hydroxylase)-1, were significantly correlated with the rate of survival, photosynthetic parameters and chlorophyll fluorescence parameters. Furthermore, metabolites related to the detoxification of graphene oxide were enriched in the flavonoid metabolic pathways. These results collectively indicate that graphene oxide can be used as a regulator of corn growth and provide insights into their use to improve crops in areas that are contaminated with nicosulfuron.

## 1 Introduction

With the continuous improvement of the living standards of Chinese residents, the consumption of sweet corn (*Zea mays*) has also been increasing yearly. The breeders are selecting varieties with higher contents of sugar in the grains to meet the market demand. The high sugar content of the sweet corn grains will inevitably reduce the vitality of seeds, which leads to slow growth and development of the canopy and an inability to manage the increasingly fierce competition from weeds in the field (Williams and Boydston, 2013; Wu et al., 2022). Therefore, the use of herbicides to control weeds in the field is an inevitable choice during the production of sweet corn. Nicosulfuron (NIF) is a sulfonylurea herbicide. Since 1999, nicosulfuron has been approved as a post-sweet corn herbicide in some areas of the US, and serious harm caused by nicosulfuron in different varieties of sweet corn has been frequently reported (Choe and Williams, 2020). Therefore, enhancing the tolerance of sweet corn to NIF is the focus of attention in production.

NIF, as a sulfonylurea herbicide, can reduce the biosynthesis of branched-chain amino acids by inhibiting the activity of acetylhydroxylate synthase (AHAS; also known as acetyllactate synthase, E.C. 4.1.3.18), which affects physiological processes, such as photosynthesis and respiration, in plants, and ultimately leads to their death (Kapusta et al., 1995; Wang et al., 2018). Different tolerant varieties of sweet corn vary in their rates of the metabolic degradation of NIF. Compared to sensitive varieties, varieties that are highly tolerant metabolize and degrade NIF more quickly (Dobbels and Kapusta, 1993). Studies have shown that isoforms of cytochrome P450 (CYP450) play an important role in the metabolism of NIF (Bathe and Tissier, 2019; Brazier-Hicks et al., 2022). Fear and Swanson (1996) studied the mechanism of resistance to monuron at the seedling stage of cotton (*Gossypium hirsutum*) and first found that P450 in microsomes participates in the metabolic activities of herbicides (Fear and Swanson, 1996). Subsequently, many studies have demonstrated that P450s are involved in the metabolic activity of NIF in microsomes. Persans et al., (2001) showed that increasing the activity of CYP450 can reduce the damage of NIF to crops (Persans et al., 2001). Many studies have demonstrated that the sensitivity of corn to herbicides metabolized by P450 is primarily regulated by a single CYP gene or a group of closely linked CYP genes on the short arm of chromosome 5 (Melissa et al., 2022; Muhammad et al., 2020). However, currently, studies on the level of expression of the P450 proteins after spraying sulfonylurea herbicides at the seedling stage and the changes in the quantity of P450 proteins in plants before and after their degradation by herbicides remain unclear, which may be related to the complexity of the P450 genes (Barrett, 2000; Frey et al., 1995). The results of the whole genome sequencing of rice (*Oryza sativa*) showed that there were 328 genes related to P450s and 99 hypothetical ones, and the functions of most of these genes remain unknown. In addition, the low abundance of the transcription of P450 genes also increases the difficulty of studying them (Pataky et al., 2008). Therefore, further in-depth research and exploration are needed to improve the activity and quantity of CYP450 proteins in crops from the perspective of the levels of gene expression, thereby enhancing the tolerance of corn to nicosulfuron.

Graphene oxide (GO) is one of the fastest-growing nanomaterials in recent years and has been widely used in many fields, such as agriculture, biomedicine, and electronics. There are very broad prospects for applying GO in the field of agriculture. In recent years, the application of GO in fertilizer slow release, plant pest control, and plant growth regulation has become a research hotspot (An et al., 2017; Cheraghi et al., 2022; Tong et al., 2018; Wang et al., 2019). Numerous studies have shown that high doses of GO are toxic to plant growth in agricultural applications, while low doses of this compound have beneficial effects on crop growth, which is known as the hormesis effect. Ren et al. (2020) reported that treatment with a high concentration of GO (1,000 mg L^–1^) exacerbates oxidative stress in wheat (*Triticum aestivum*) and inhibits the growth of its roots (Ren et al, 2020). You et al. (2021) revealed that high concentrations of GO reduced the uptake of cadmium by rice but also limited its growth (You et al., 2021). In contrast, Guo et al., (2021) showed that low concentrations of GO (50 and 100 mg L^−1^) effectively increased the accumulation of dry matter in tomato (*Solanum lycopersicum*) plants and promoted the growth of their roots. Sharma et al., (2020) determined that GO significantly increases the chloroplast activity of spinach (*Spinacea oleracea*) leaves, thereby improving their electron transfer efficiency and enhancing their photosynthetic capacity.

The metabolome is a collection of small molecular chemical entities involved in metabolism. It is a powerful tool to identify and quantify plant metabolites, such as sugars, amino acids, and organic acids, in various metabolic pathways to reflect the physiological characteristics of plant metabolism. This is one of the most important technologies that is widely used to study the physiological mechanisms of plant stress resistance. Previous studies have found that GO-NIF, formed by the combination of GO as a safety agent and NIF, can effectively enhance the resistance of sweet corn to NIF. This study utilized a pair of sweet corn sister lines with different levels of resistance to nicosulfuron (HK301 and HK320, which were resistant and sensitive to nicosulfuron, respectively). GO-NIF stress significantly improved the rate of survival of the HK320 seedlings compared to NIF stress under the same concentration of stress (80 mg kg^-1^). However, the mechanism of detoxification of GO as an NIF safety agent remains unclear.

Therefore, in a recent study, we continued to study GO as a safety agent for NIF to detect the survival rate (SR), physiological indicators, and differential metabolites in a pair of sweet corn sister lines. Broad-targeted metabolomics based on the use of liquid chromatography-mass spectrometry (LC-MS) was used to identify metabolites in two sweet corn sister lines following treatment with GO and NIF. Our results showed that the application of GO affected the SR and physiological indices of the inbred lines of sweet corn. A metabolomic analysis revealed the mechanism of how GO regulates the toxicity of NIF in inbred lines of sweet corn.

## 2 Materials and methods

### 2.1 Plant material

The materials used in this study were two sister inbred lines of sweet corn, which were cultivated by the Hebei Normal University of Science and Technology (Qinhuangdao, China). These two sister inbred lines of sweet corn differed significantly in their characteristics of tolerance to NIF. HK301 was tolerant to NIF, while HK320 was sensitive to it (Wu et al., 2022).

### 2.2 Methods

In 2021–2022, field herbicide concentration screening experiments were conducted at a series of sites in China, including the experimental station (40°4’N, 118°95’E) in Qinhuangdao City, Hebei Province; at the Zhejiang Academy of Agricultural Sciences in Dongyang City (28°63’N, 120°31’E), Zhejiang Province; and at the Zhejiang Province South Breeding Experimental Base in Sanya City (18°34’N, 108°42’E), Hainan Province. The results showed that 80 mg kg^-1^ GO+NIF could effectively improve the resistance of sensitive sweet corn inbred line HK320 to nicotinuron (Wang et al., 2024). In 2022–2023, a field test was conducted at the Changli Experimental Station (40°4’N, 118°95′E) in Qinhuangdao City, Hebei Province. The experiment adopted a randomized block design with three replicates. Each test plot was 5 m long, with 10 rows and a plot area of 30 m^2^. Three seeds were planted at each point to ensure an appropriate number of seedlings. The sweet corn seedlings at the four-leaf stage were sprayed with water (CK), GO (80 mg kg^-1^), NIF (80 mg kg^-1^), and GO+NIF (80 mg kg^-1^) using an electric backpack sprayer. Two days after spraying, the physiological indices, including the activity of superoxide dismutase (SOD), peroxidase (POD), ascorbate peroxidase (APX), and catalase (CAT), and the content of superoxide anions (O_2_^-^) and hydrogen peroxide (H_2_O_2_) were evaluated under the GO, NIF, and GO+NIF treatments, respectively. The physiological indices were determined as previously described (Wang et al., 2018). The photosynthetic indicators, including the photosynthetic rate (PR), stomatal conductance (SC), transpiration rate (TR), intercellular CO_2_ concentrations (Ci), and limited stomatal value (Ls), were measured using an Li-6800 (LI-COR, Lincoln, NE, USA), and the chlorophyll fluorescence parameters [quantum yield of PSII photochemistry (Fv/Fm), photochemical quenching coefficient (qP), electron transport rate (ETR), and nonphotochemical quenching (NPQ)] were calculated by a chlorophyll fluorometer PAM2500 (Heinz Walz GmbH, Effeltrich, Germany) (Wu et al., 2022). After 2 weeks of spraying, the SR of the corn seedlings under different treatments was evaluated. SPSS 20.0 (IBM, Inc., Armonk, NY, USA) was used for one-way analyses of variance (ANOVAs). A *post-hoc* ANOVA was performed using the LSD, Tukey’s b, and Waller–Duncan methods. The power analysis of the sample size was conducted by MetaboAnalyst 6.0. The sample size is sufficient when the power value is greater than 0.8 (Luedicke, 2013).

In June 2022, the same experiment was conducted in the field at the Changli Experimental Station (40°4′N, 118°95′E). Changli County has a temperate continental monsoon season, with an average annual accumulated temperature of 3,960°C and an average annual precipitation of 680 mm. The soil type of the test site was medium loam. The content of organic matter in the soil was 18.47 g kg ^– 1^, total nitrogen was 1.51 g kg^-1^, alkaline hydrolysis nitrogen was 109.32 mg kg^-1^, available phosphorus was 17.32 mg kg^-1^, and the available potassium was 74.35 mg kg^-1^. The sweet corn seedlings at the four-leaf stage were sprayed with the CK, 80 mg kg^-1^ GO, NIF, and GO+NIF, respectively. At 24 h after spraying, the aboveground leaves of the seedlings as samples were frozen in liquid nitrogen and stored at -80°C for an additional metabolome analysis, and each treatment was repeated three times. The treated HK301 and HK320 were designated H01_CK, H01_G, H01_N, H01_GN, H20_CK, H20_G, H20_N, and H20_GN, respectively, where H01 indicates HK301, H20 indicates HK320, G indicates GO, N indicates NIF, and GN indicates GO+NIF.

### 2.3 Analyses of the metabolic components

Samples (100 mg) were transferred to 2 mL Eppendorf tubes and resuspended in 500 μL extract solvent (methanol - water = 1:1). After vortexing for 30 s, the samples were homogenized at 35 Hz for 4 min and sonicated in an ice-water bath for 15 min. Extraction was performed overnight at 4°C on a shaker. Then, the samples were centrifuged at 12,000 rpm for 15 min at 4°C. The supernatant was carefully filtered through a 0.22 microporous membrane and transferred to 2 mL glass vials. A 30 μL aliquot from each sample was mixed to prepare a quality control (QC) sample. Finally, the supernatant (50 μL) was transferred to LC vials for subsequent LC-MS analysis. A phase of the liquid chromatography was an aqueous solution that contained 0.1% formic acid, and the B phase was acetonitrile. The temperature of the column oven was 40°C; the temperature of the autosampler was 4°C, and the injection volume was 2 μL. In this study, a mass spectrometry analysis was performed in multiple reaction monitoring (MRM) mode using a SCIEX 6500 QTRAP+ Triple Quadrupole Mass Spectrometer with an IonDrive Turbo V ESI ion source. The ion source parameters were as follows: IonSpray voltage: +5500/-4500 V; curtain gas: 35 psi; temperature: 400°C; ion source gas 1: 60 psi; and ion source gas 2: 60 psi, DP: ± 100 V. The ion source was analyzed in the MRM mode.

### 2.4 Data processing and multivariate analysis

For the 27 samples, missing values in the raw data were simulated using the minimum half method. The data were then filtered to retain only the peaks with missing values <50% of the actual samples, and the filtered data were normalized using each sample’s features combined to perform a sumNormalizer. After the high-quality data was obtained, we performed a series of multivariate model recognition analyses on it. A total of 27 samples were subjected to a correlation analysis, principal component analysis (PCA), and orthogonal partial least squares discriminant analysis (OPLS-DA). The OPLS-DA model between each two groups was established. The current OPLS-DA model was more reliable when the model parameters (R2 and Q2) were higher. Simultaneously, to check the robustness and predictive ability of the OPLS-DA model, 200 permutations were performed. A univariate statistical analysis (UVA) was utilized to screen for differential metabolites. A hierarchical cluster analysis of the differential metabolites was performed using the R environment, and the second generation of differential metabolites was submitted to the Kyoto Encyclopedia of Genes and Genomes (KEGG) website for an enrichment analysis of the related pathways.

## 3 Results

### 3.1 GO affects the SR and physiological index of sweet corn seedlings

**Figure 1:**
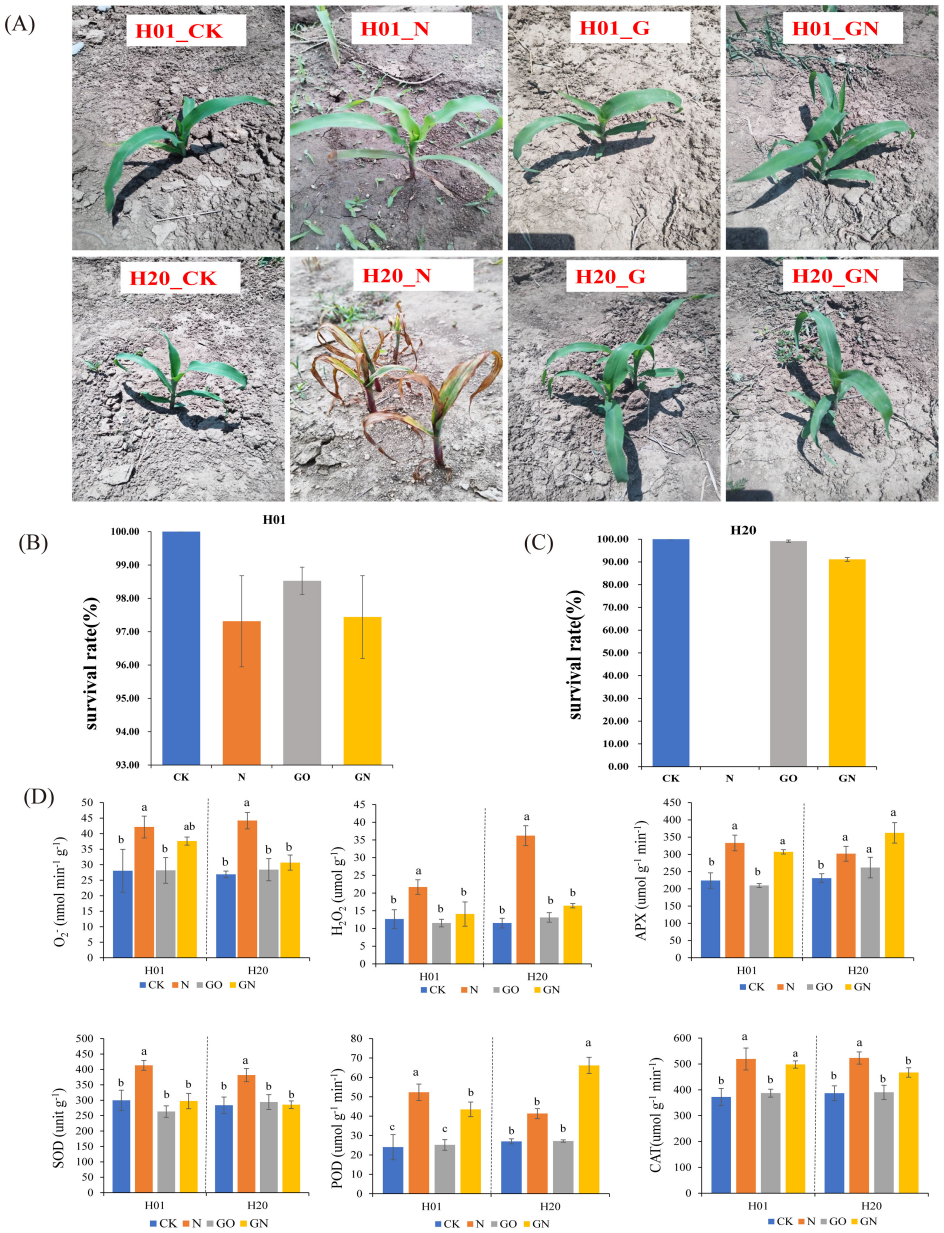
Effects of GO on the growth of sweet corn seeding. **(A)** phenotype of seedings under different treatment; **(B)** SR of H01; **(C)** SR of H20; **(D)** physiological index. Small letters (a, b) indicate differences between values obtained on treatment (*P* < 0.05) according to a least significant difference (LSD) test.

Previous studies showed that inbred line H01 was tolerant to NIF, while inbred line H20 was sensitive to this chemical (Wu et al., 2022). In this study, we found that NIF inhibited the growth of both sweet corn sister inbred line seedlings (**Figure 1A**). The mean value of the SR of the H01 lines was 97.31%, while the values of the H20 lines were 0 under the NIF treatment (**Figure 1B**). To explore whether GO played a role in the detoxification of NIF, we also applied GO and GO-NIF to sweet corn seedlings, respectively. The GO had no significant effects on the SR of the sweet corn seedlings compared with the CK (**Figure 1B**). Compared with the NIF-treated sweet corn seedlings alone, GN increased the SR of the H01 and H20 inbred line seedlings. In particular, the SR of H20 increased to 91.10% (**Figure 1C**). Moreover, the activity of SOD, POD, APX, and CAT and the content of H_2_O_2_, and O_2_^-^ were used to evaluate the physiological changes of different tissues under various treatments. The results showed that all the physiological indices of the H01 and H20 seedlings increased significantly under the NIF treatment compared with the CK. Compared with the sweet corn seedlings treated with NIF alone, the GO markedly decreased the physiological index of the H01 and H20 inbred lines of seedlings (**Figure 1D**). These results indicated that GO significantly restored the NIF-impacted corn seedling traits.

### 3.2 GO affects the photosynthetic and chlorophyll fluorescence parameters of sweet corn seedlings

**Figure 2:**
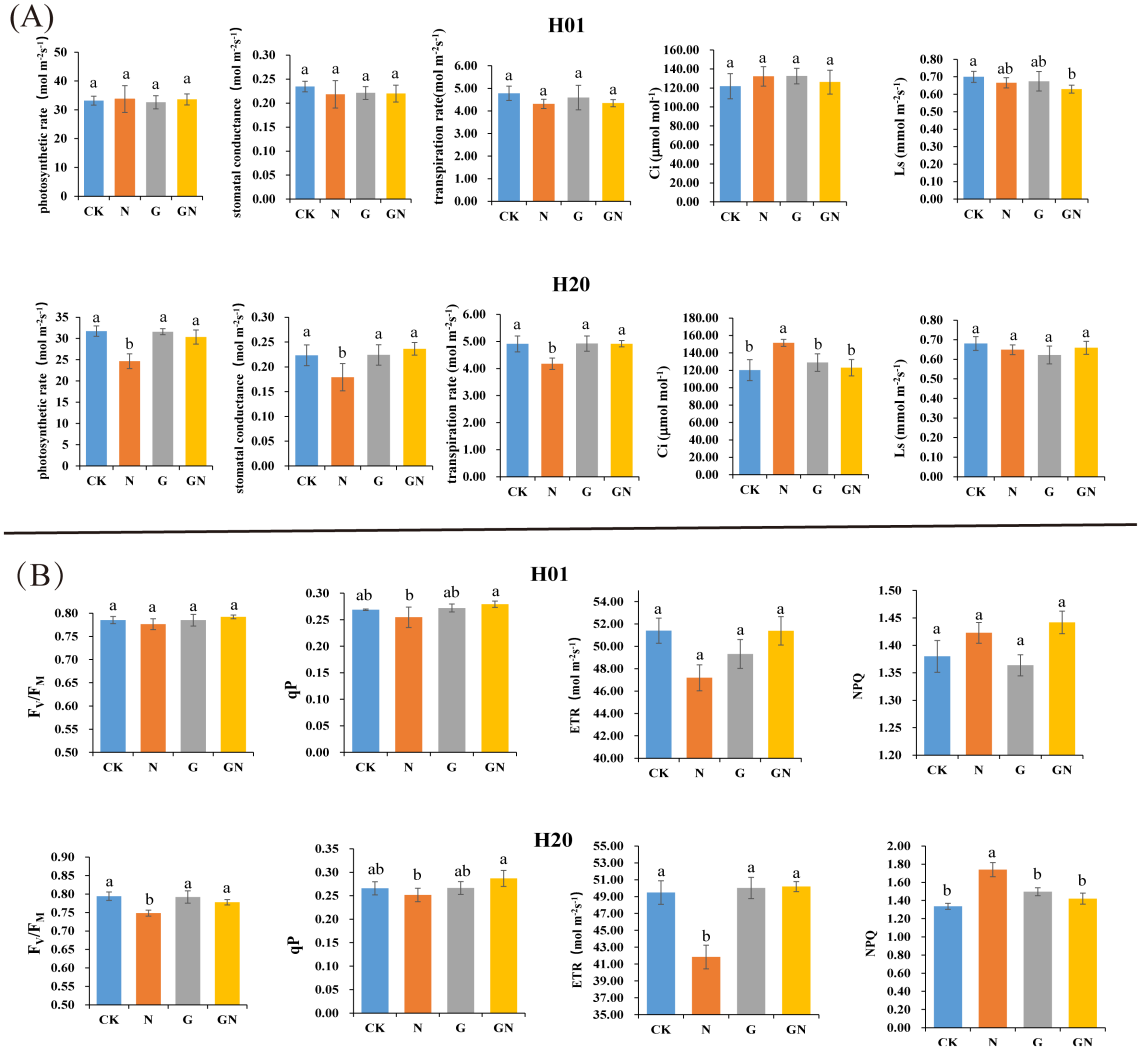
The photosynthetic parameters and chlorophyll fluorescence parameters of H01 and H20. Effects of different treatment on the **(A)** photosyenthetic and **(B)** chlorophyll flouroscence parameters. Small letters (a, b) indicate differences between values obtained on treatment (*P* < 0.05) according to a least significant difference (LSD) test.

At 2 days after spraying, compared with the CK, the photosynthetic parameters of the H01 seedlings treated with NIF did not change significantly. Compared with the NIF treatment, the GN did not significantly affect the various parameters in the H01 inbred lines (**Figure 2A**). However, the NIF treatment significantly affected the PR, SC, TR, and Ci parameters compared to the CK. Furthermore, the GN treatment significantly increased the PR, SC, and TR parameters compared with the NIF treatment in the H20 inbred lines (**Figure 2A**). There were no significant differences in the Fv/FM, qP, and NPQ between the different treatments in the H01 inbred lines. However, in the H20 corn inbred lines, the NIF treatment significantly affected the Fv/FM, qP, ETR, and NPQ parameters compared to those of the CK. Furthermore, GN significantly improved the chlorophyll fluorescence parameters compared to those of the NIF treatment (**Figure 2B**).

These results provided additional evidence that the H20 inbred lines were more sensitive to NIF, and treatment with GO improved the effect of NIF on the chlorophyll fluorescence parameters of H20, thus, improving the efficiency of photosynthesis.

### 3.3 Principal component analysis

**Figure 3:**
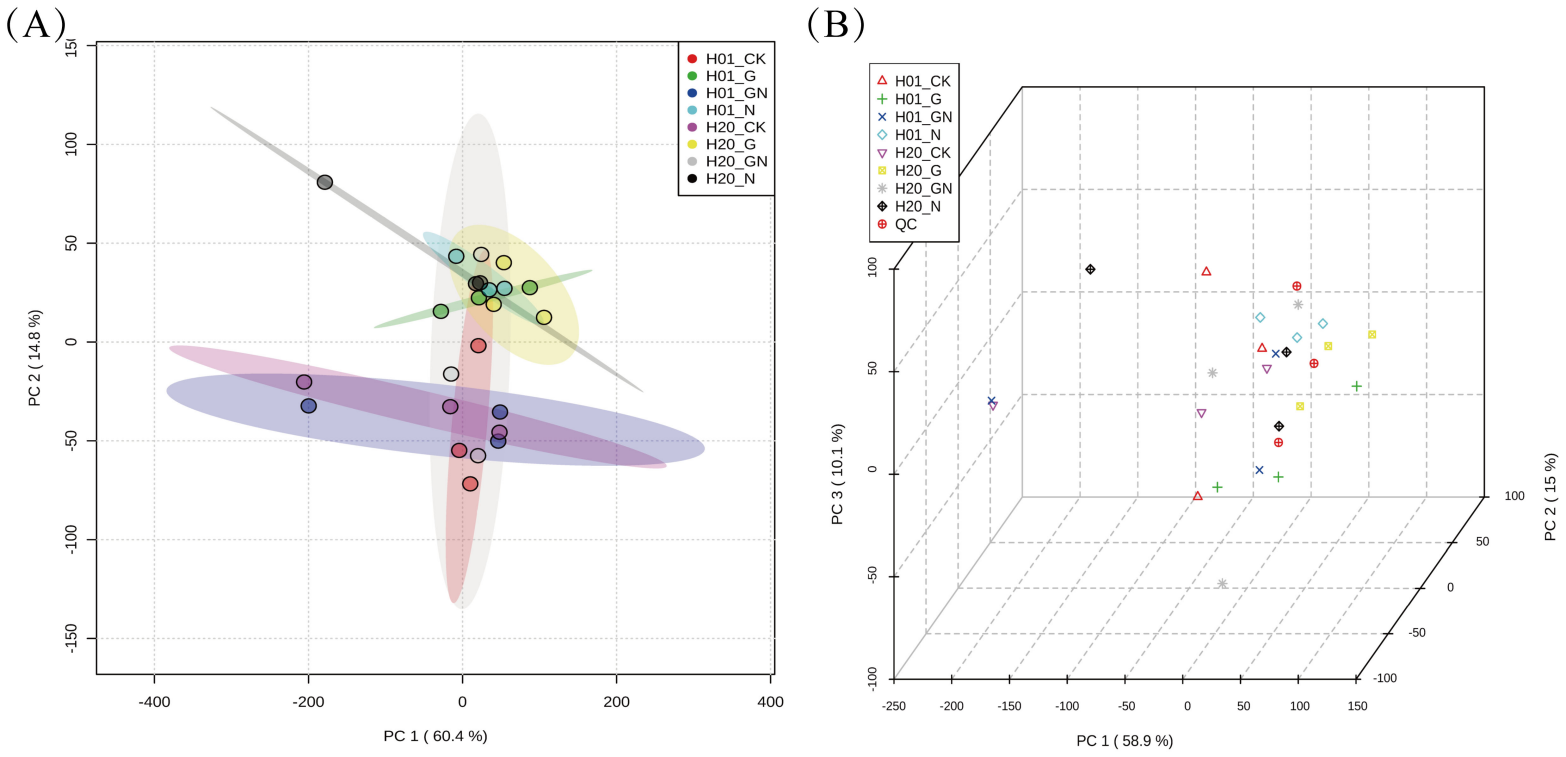
PCA analysis of different treatments. **(A)** 2D score scatter plot of all samples. **(B)** 3D score scatter plot of all samples. Each point in the figure represents a sample, and different colors represent different groups.

The correlation analysis showed that the correlation coefficients of the three replicates of the samples were all > 0.8, which proved the reproducibility of the experiment and the reliability of the results (**Supplementary Figure S1**). In addition, a power analysis of the correlation coefficient showed that the effect value reached 0.90, which showed that the experimental sample was of sufficient size. A PCA was performed on the obtained collated data, and the results are shown in **Figures 3A**, **B**. The three QC samples were clustered together and close to the center of the samples. This indicated that the experiment was highly reproducible. To further understand the within-group differences between the two inbred lines of sweet corn, PCA analyses were performed in the different groups. In the PCA score plot, H01_CK and H01_N were far apart, H01_N and H01_GN were far apart, H20_CK and H20_N were far apart, and H20_N and H01_GN were far apart, which indicated that there was a substantial difference in the metabolites between those in the CK and those treated with the herbicides. This suggested that the incorporation of NIF and GO significantly affected the metabolic process of the inbred corn lines H01 and H20.

### 3.4 OPLS-DA analysis

From the results of the OPLS-DA score chart, all the groups of samples scored significantly, with 95% confidence intervals for all the samples. To compare the variability between the different groups of samples in more detail, an analysis was performed using an OPLS-DA with a substitution test. The different treated groups were also clearly separated in the OPLS-DA. In the permutation test of the OPLS-DA, R2 is the explanatory rate of the model, and Q2 is the predictive power of the model. The statistics of the permutation test showed that the Q2 for H01_N vs H01_CK was 0.59, H01_G vs H01_CK was 0.638, H01_GN vs H01_N was 0.67, H20_GN vs H20_CK was 0.723, H20_GN vs H20_N was 0.64, and H20_N vs H20_CK was 0.691 (**Supplementary Figure S2**). The results indicated that there was significant variation in the metabolites of the different inbred lines in the various treatments.

### 3.5 Analysis of the differential metabolites within the groups

**Figure 4:**
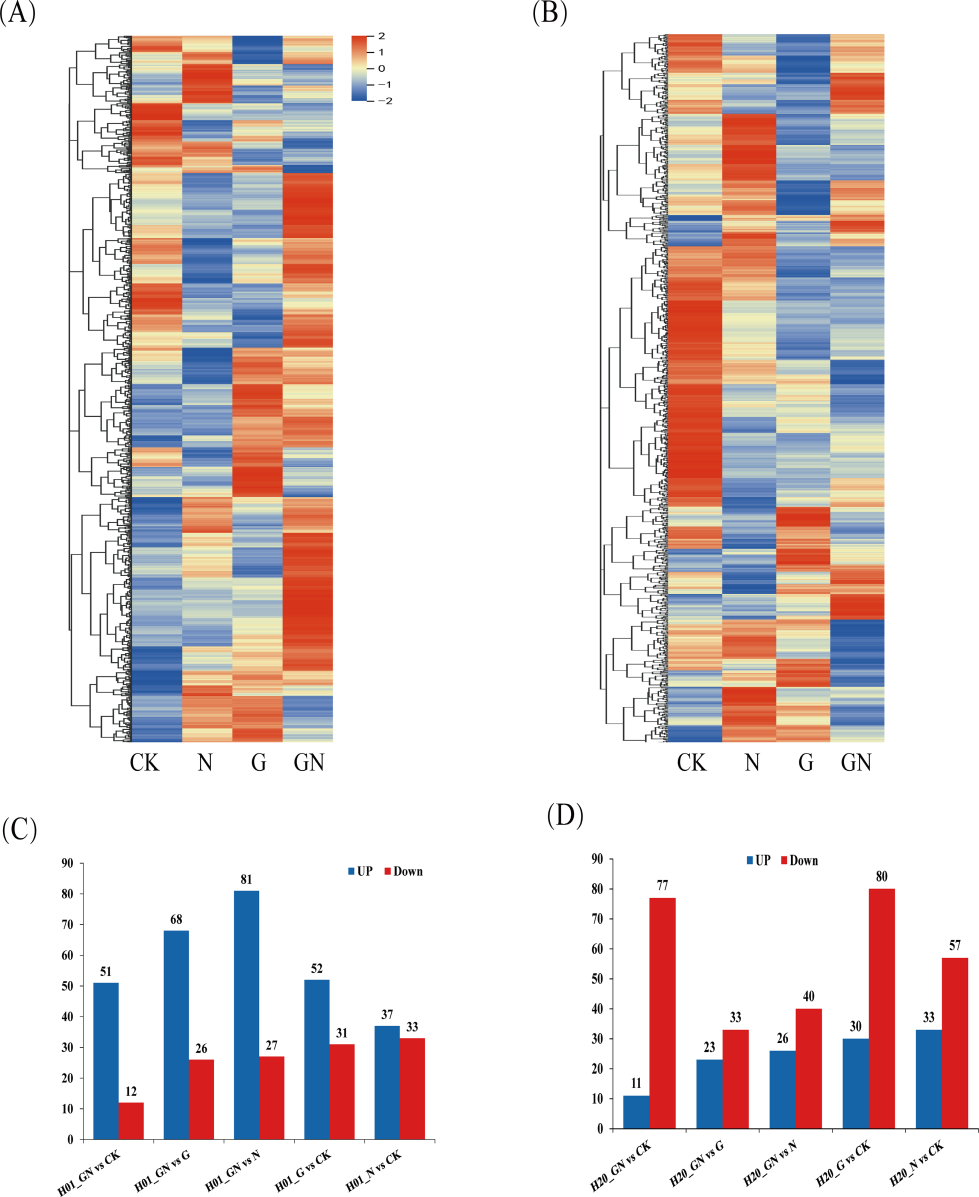
Differentially expressed metabolites among treatments. **(A)** Heat maps of the detected metabolites in the H01 seedings and **(B)** H20 seedings; DEMs in the H01 seedings **(C)** and H20 seedings **(D)**.

To explore the effects of NIF and GO-NIF on the metabolites of sweet corn, the variable importance of projection (VIP) value of the OPLS-DA model (threshold >1) and the p-value of Student’s *t*-test (threshold<0.05) were used to screen for differentially expressed metabolites (DEMs). The results showed that there were different metabolites in the varying groups. The pattern of expression of these DEMs was divided into eight groups based on a K-means clustering algorithm (**Supplementary Figure S3**). A total of 224 and 314 DEMs were identified in inbred lines H01 and H20, respectively (**Figures 4A, B**; **Supplementary Table S1**). Compared with the CK, there were more upregulated metabolites than downregulated ones after the NIF treatment. H01_GN vs N had the most metabolites (108), and there were significantly more upregulated metabolites (81) than downregulated ones (27) in the H01 inbred lines. Among all the treatments in the H20 inbred lines, compared with the CK, there were fewer upregulated metabolites (33) than downregulated metabolites (57) after the NIF treatment.

**Figure 5:**
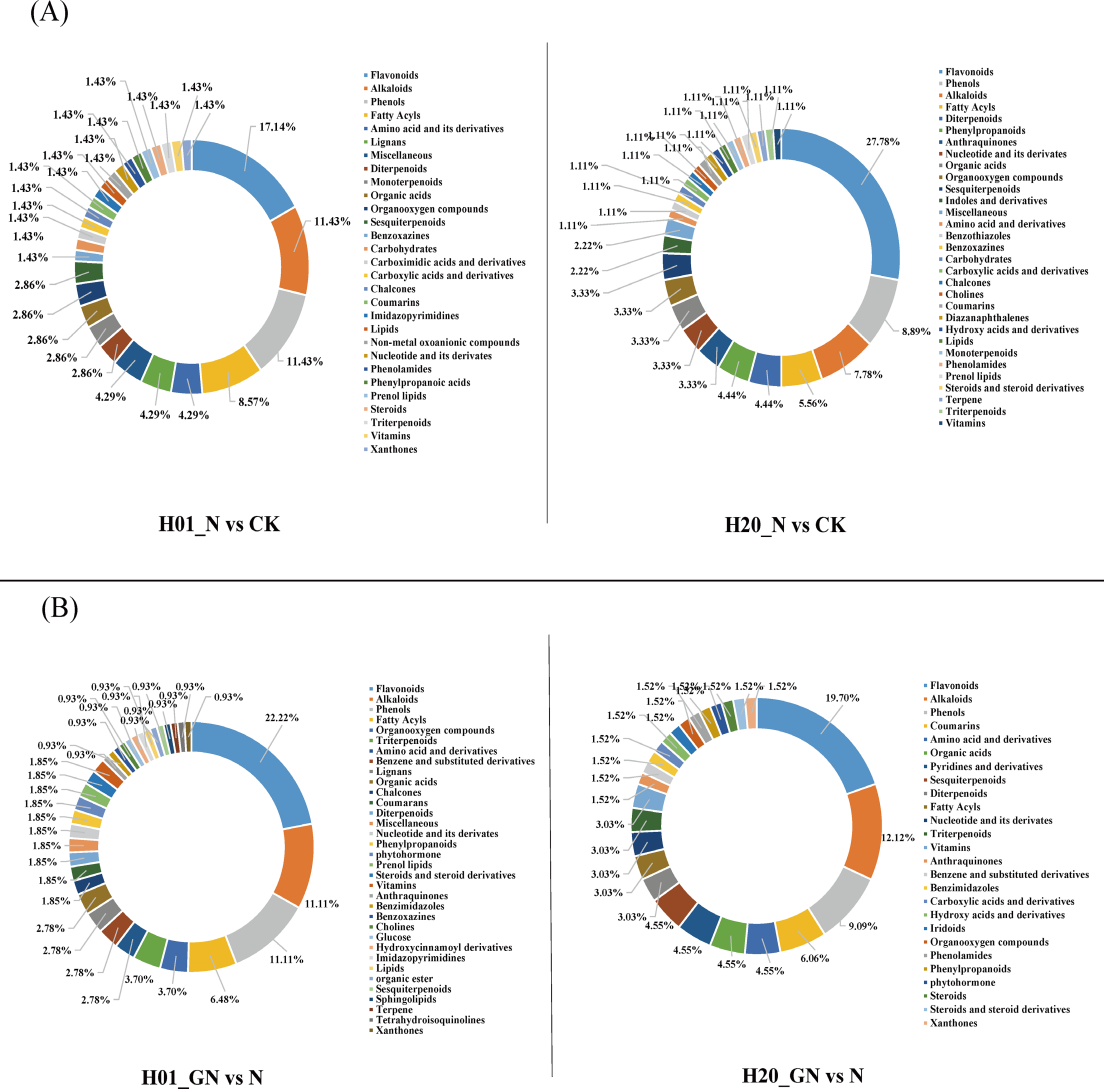
**(A)** Metabolite classes detected in H01_N vs CK and H20_N vs CK groups. **(B)** Metabolite classes detected in H01_GN vs N and H20_GN vs N groups.

Classification of the differential compounds of DEMs showed that the H01_N vs CK group was primarily composed of flavonoids (17.14%), alkaloids (11.43%), phenolics (11.43%), fatty acyls (8.57%), amino acids and their derivatives (4.29%), lignans (4.29%), and miscellaneous (4.29%). The H20_N vs CK group primarily consisted of flavonoids (27.78%), alkaloids (8.89%), phenolics (7.78%), fatty acyls (5.56%), diterpenoids (4.44%), and phenylpropanoids (4.44%) (**Figure 5A**). Furthermore, the H01_GN vs N group primarily contained flavonoids (22.22%), alkaloids (11.11%), phenolics (11.11%), and fatty acyls (6.48%). The H20_GN vs N group mostly consisted of flavonoids (19.70%), alkaloids (12.12%), phenolics (9.09%), coumarins (6.06%), amino acids and derivatives (4.55%), organic acids (4.55%), pyridines and derivatives (4.55%), and sesquiterpenoids (4.55%) (**Figure 5B**).

### 3.6 Metabolic characteristics of the different corn lines under NIF stress

The treatment with NIF resulted in 70 DEMs in the H01 inbred line. However, it resulted in more DEMs in the H20 inbred line (**Supplementary Figure S4**). In the H01 seedlings, the NIF treatment increased 37 DEMs, including two alkaloids, two amino acids and derivatives, five fatty acyls, five flavonoids, two lignans, two miscellaneous, two monoterpenoids, two organooxygen compounds, two phenolics, and two sesquiterpenoids. In contrast, it reduced other DEMs, including six alkaloids, seven flavonoids, and five phenolics (**Figure 4C**; **Supplementary Table S2**). The NIF treatment increased 33 DEMs in the H20 seedlings, including two diterpenoids, four fatty acyls, two flavonoids, two nucleotides and their derivatives, two organooxygen compounds, six phenolics, and two sesquiterpenoids. In contrast, it decreased 57 DEMs, including six alkaloids, two anthraquinones, two diterpenoids, 23 flavonoids, two phenolics, and four phenylpropanoids (**Figure 4D**; **Supplementary Table S2**).

The Venn diagram analysis showed that NIF stress increased 14 metabolites, including three fatty acyls (octadecanamide, oleamide, and oleic acid), two organooxygen compounds (mannose 6-phosphate and glucose 1-phosphate), in both the H01 and H20 seedlings. Moreover, the NIF treatment decreased the DEMs, including four flavonoids (isoscoparin, isosakuranetin, morin, and “2,3,5,7-tetrahydroxyflavone”), one benzoxazine [(R)-2-hydroxy-2-hydroxylase-1,4-benzoxazin-3(4H hydroxylase)-1], one fatty acyl (suberic acid), one organic acid (2-picolinic acid) and one Prenol lipid (norbixin) in both the H01 and H20 seedlings. In contrast, the NIF treatment increased one miscellaneous (β-cryptoxanthin) in the H01 seedlings but decreased it in the H20 seedlings compared with the CK (**Supplementary Figure S5**; **Supplementary Table S2**). These results suggest that NIF stress induces different metabolites in the two inbred lines. Moreover, NIF stress had a more significant impact on the biosynthesis/degradation of metabolites in the H20 lines.

### 3.7 Effects of GO on the different corn lines metabolomes under NIF stress

**Figure 6:**
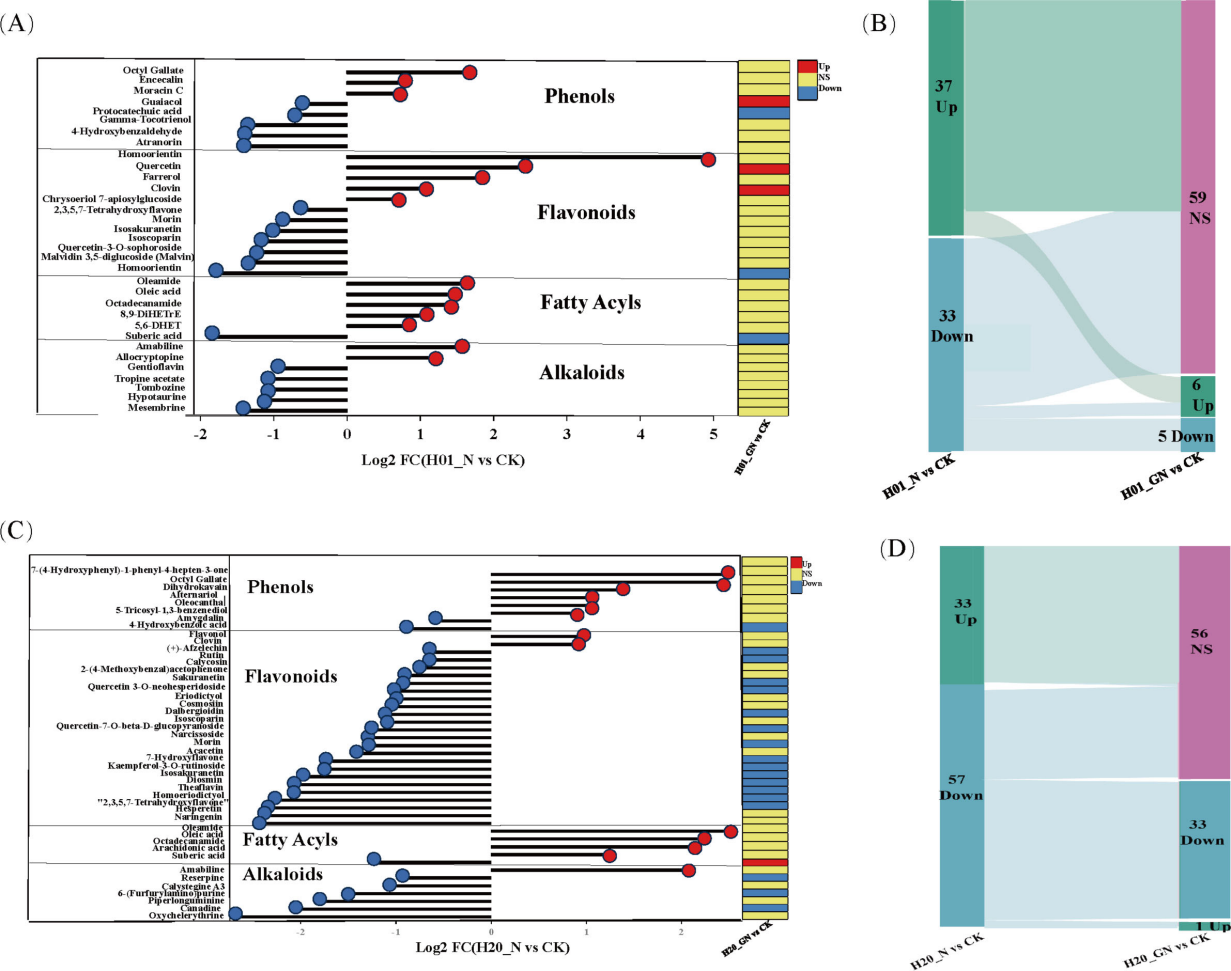
NIF-induced DEMs in different inbred lines. **(A)** The significant DEMs in response to NIF in the H01 seedings. **(B)** Sankey diagram of DEMs in different treatments. **(C)** The significant DEMs in response to NIF in the H20 seedings. **(D)** Sankey diagram of DEMs in different treatments. Up, upregulated metabolites; Down, downregulated metabolites; NS, non-significant metabolites.

In the NIF-treated sweet corn seedlings, most of the DEMs included phenolics, flavonoids, fatty acyls, and alkaloids. In the H01 seedlings, three phenolics, five flavonoids, five fatty acyls, and two alkaloids were upregulated. However, five phenolics, seven flavonoids, one fatty acyl, and five alkaloids were downregulated (**Figure 6A**). Furthermore, we found that GO recovered 59 NIF-responsive metabolites to more normal levels in the H01 corn seedlings (**Figure 6B**; **Supplementary Table S3**). In the H20 seedlings, six phenolics, two flavonoids, four fatty acyls, and one alkaloid increased, while two phenolics, 23 flavonoids, one fatty acyl, and six alkaloids decreased (**Figure 6C**). In addition, the GO treatment restored 56 NIF response metabolites to standard levels in the H20 seedlings (**Figure 6D**; **Supplementary Table S3**), and 16 common metabolites, such as amabiline, miltirone, oleic acid, and Isoscoparin, changed to normal levels in both the H01 and H20 sweet corn seedlings under the GO treatment (**Supplementary Table S3**). These metabolites may contribute to the ability of sweet corn to detoxify NIF.

Compared with the NIF treatment alone, GN induced 81 DEMs, which primarily included flavonoids, phenolics, alkaloids, fatty acyls, phytohormones, steroids and steroid derivatives, organic acids, and vitamins in the H01 seedlings (**Supplementary Figures S6A, B**, **S7A**; **Supplementary Table S4**). Furthermore, GN changed 66 DEMs, mostly including flavonoids, alkaloids, phenolics, fatty acyls, vitamins, phytohormones, steroids, steroid derivatives, and organic acids in the H20 seedlings compared with the NIF-treated seedlings (**Supplementary Figures S6C, D**, **S7B**; **Supplementary Table S4**). The Venn diagram analysis showed that GN increased 10 metabolites, including five flavonoids, one alkaloid, one fatty acyl, one organic acid, one steroid and steroid derivative and one vitamin, while it decreased the number of alkaloids (petasitenine) and flavonoids (chrysoeriol 7-apiosylglucoside) in both the H01 and H20 seedlings compared with the NIF-treated seedlings (**Supplementary Figure S8**; **Supplementary Table S4**). In contrast, this treatment increased three DEMs, including one alkaloid (hordenine), one flavonoid (saponarin), and one phenolic (sesamol) in the H01 seedlings but decreased these in the H20 seedlings compared with the NIF-treated seedlings (**Supplementary Figure S8**; **Supplementary Table S4**). These results indicated that GO regulates varying metabolic profiles under NIF stress in the different lines of sweet corn.

### 3.8 Comparative analyses of the DEMs of the seedlings treated with GO under CK and NIF stress

GO increased 52 metabolites and reduced 31 DEMs in the H01 seedlings (**Figure 4C**; **Supplementary Table S1**), while it increased 30 metabolites and decreased 80 metabolites compared with the CK in the H20 seedlings (**Figure 4D**, **Supplementary Table S1**). The Venn diagram analysis showed that GO increased 12 common metabolites and reduced five mutual metabolites in both the H01 and H20 inbred lines (**Supplementary Figure S9A**; **Supplementary Table S1**). Compared to the CK, GN increased 51 DEMs and reduced 12 DEMs in the H01 seedlings (**Figure 4C**; **Supplementary Table S1**). Furthermore, GN increased 11 metabolites but decreased 76 metabolites in the H20 seedlings (**Figure 4D**; **Supplementary Table S1**). The Venn diagram showed that GO increased two mutual metabolites (deoxyguanosine and “3,4-dihydroxybenzaldehyde”), but it reduced one phenolic (protocatechuic acid) in both the H01 and H20 seedlings. In addition, GN increased the level of XX in the H20 seedlings, but GO decreased the level of fatty acyls and triterpenoids compared to the CK in H01 seedlings (**Supplementary Figure S9B**; **Supplementary Table S1**). These results indicate that these two sweet corn inbred lines regulate the tolerance to NIF through similar or different mechanisms.

### 3.9 Correlation analysis of the DEMs and growth parameters and the physiological index of the sweet corn seedlings

**Figure 7:**
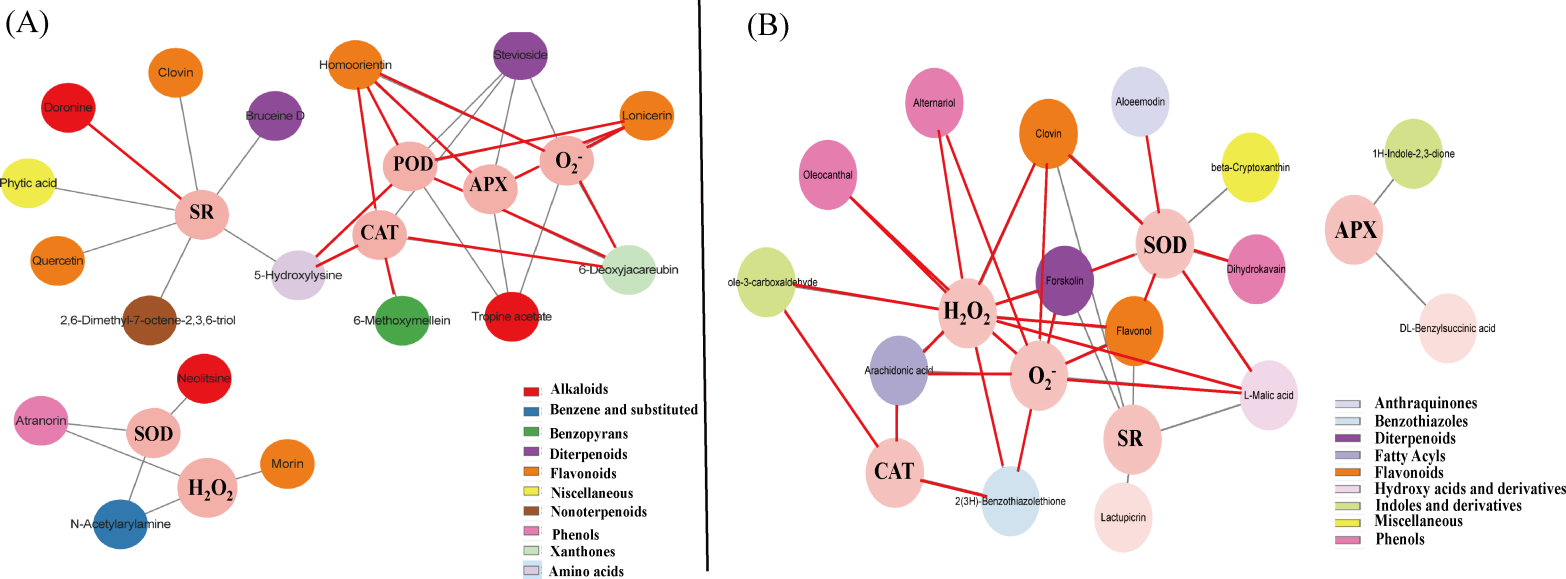
Correlation analysis between SR, physiological parameters, and DEMs. **(A)** The correlation network of DEMs with SR and physiological parameters in H01. **(B)** The correlation network of DEMs with SR and physiological parameters in H20. The red lines indicate positive correlations, and the gray lines indicate negative correlations. Different color represents a different class of metabolites.

Previous studies have shown that GO can significantly reduce the damage of NIF to sweet corn. Moreover, the DEMs were related to the SR and physiological indices of sweet corn seedlings. To confirm this, we performed a Pearson correlation coefficient analysis on the mean of DEMs and SR, and the physiological indices, including the activity of SOD, POD, and APX and the content of H_2_O_2_ and O_2_^-^. In the H01 seedlings, an alkaloid (doronine) was positively correlated with the SR, whereas an amino acid (5-hydroxylysine), diterpenoid (bruceine D), two flavonoids (clovin and quercetin), a miscellaneous (phytic acid), and a monoterpenoid (2,6-dimethyl-7-octene-2,3,6-triol) were negatively correlated with the SR (**Figure 7A**; **Supplementary Table S5**). A phenolic (atranorin), flavonoid (morin), benzene and substituted derivative (N-acetylarylamine), two alkaloids (neolitsine and tropine acetate), and a diterpenoid (stevioside) were negatively correlated with the physiological indices. An amino acid (5-hydroxylysine), nenzopyrans (6-methoxymellein), two flavonoids (homoorientin and lonicerin), and a xanthone (6-deoxyjacareubin) were positively correlated with the physiological indices (**Figure 7A**; **Supplementary Table S5**). In the H20 seedlings, a diterpenoid (forskolin), hydroxy acid and derivative (L-malic acid), two flavonoids (flavonol and clovin), and two sesquiterpenoids (clovin and flavonol) were negatively correlated with the SR. An anthraquinones (aloe emodin), benzothiazole (2(3H)-benzothiazolethione), diterpenoid (forskolin), fatty acyl (arachidonic acid), two flavonoids (clovin and flavonol), a hydroxy acid and derivative (L-malic acid), indole and derivative (indole-3-carboxaldehyde), and three phenolics (dihydrokavain, oleocanthal, and alternariol) were positively correlated with the physiological indices (**Figure 7B**, **Supplementary Table S6**). These results indicate that the changes in metabolites affect the SR and physiological indices of the sweet corn seedlings.

### 3.10 Correlation analysis of the DEMs and photosynthetic parameters and the chlorophyll fluorescence parameters of sweet corn seedlings

**Figure 8:**
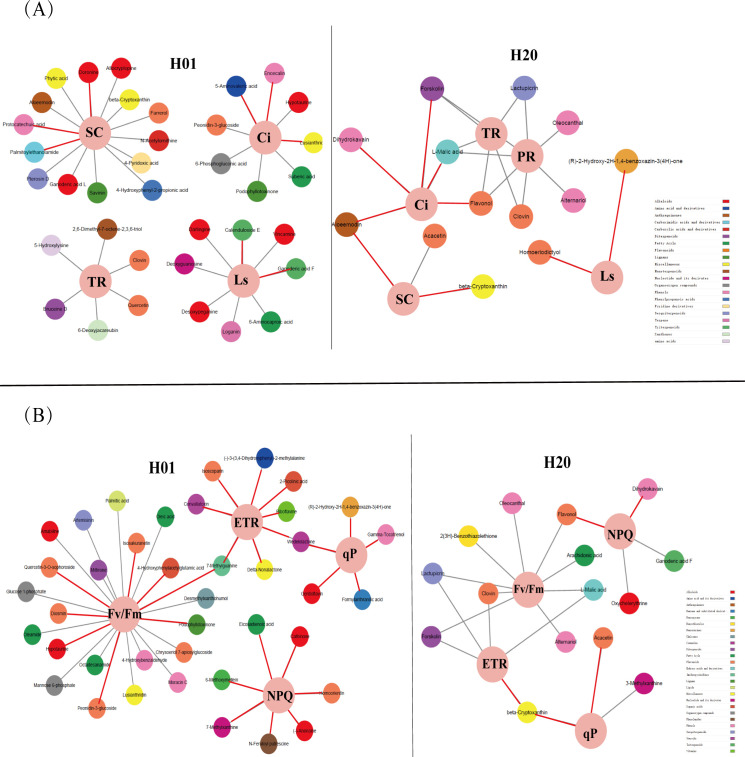
Correlation analysis between photosynthetic parameters, chlorophyll fluorescence parameters, and DEMs. **(A)** The correlation network of DEMs with photosynthetic parameters. **(B)** The correlation network of DEMs with chlorophyll fluorescence parameters. The red lines indicate positive correlations, and the gray lines indicate negative correlations. Different color represents a different class of metabolites.

We then performed a Pearson correlation coefficient analysis on the mean of the DEMs and photosynthetic parameters, as well as the chlorophyll fluorescence parameters. In the H01 seedlings, palmitoylethanolamide, doronine, and protocatechuic acid were positively correlated with SC. 7-Methylxanthine was positively correlated with PR. Ganoderic acid F and calenduloside E were positively correlated with Ls. Lusianthridin, encecalin, and 5-aminovaleric acid were correlated with Ci. For the chlorophyll fluorescence, eight DEMs were positively correlated with the ETR; eight DEMs were positively correlated with the Fv/Fm; seven DEMs were positively correlated with the NPQ, while five DEMs positively correlated with the photochemical quenching coefficient (qP) (**Figures 8A, B**, **Supplementary Tables S7**-**Supplementary Table S8**).

In the H20 seedlings, (R)-2-hydroxy-2-hydroxylase-1,4-benzoxazin-3(4-hydroxylase)-one and homoeriodictyol were positively correlated with Ls. Acacetin and beta-cryptoxanthin were positively correlated with SC. Forskolin, L-malic acid, aloeemodin, flavonol, and dihydrokavain were positively correlated with Ci. For the chlorophyll fluorescence, the ETR was positively correlated with β-cryptoxanthin. Acacetin and β-cryptoxanthin were positively correlated with qP. Flavonol and dihydrokavain were positively correlated with NPQ (**Figures 8A, B**; **Supplementary Table S7**-**S8**). These metabolites were significantly correlated with photosynthesis in the maize inbred lines.

### 3.11 Pathway analysis of the DEMs

**Figure 9:**
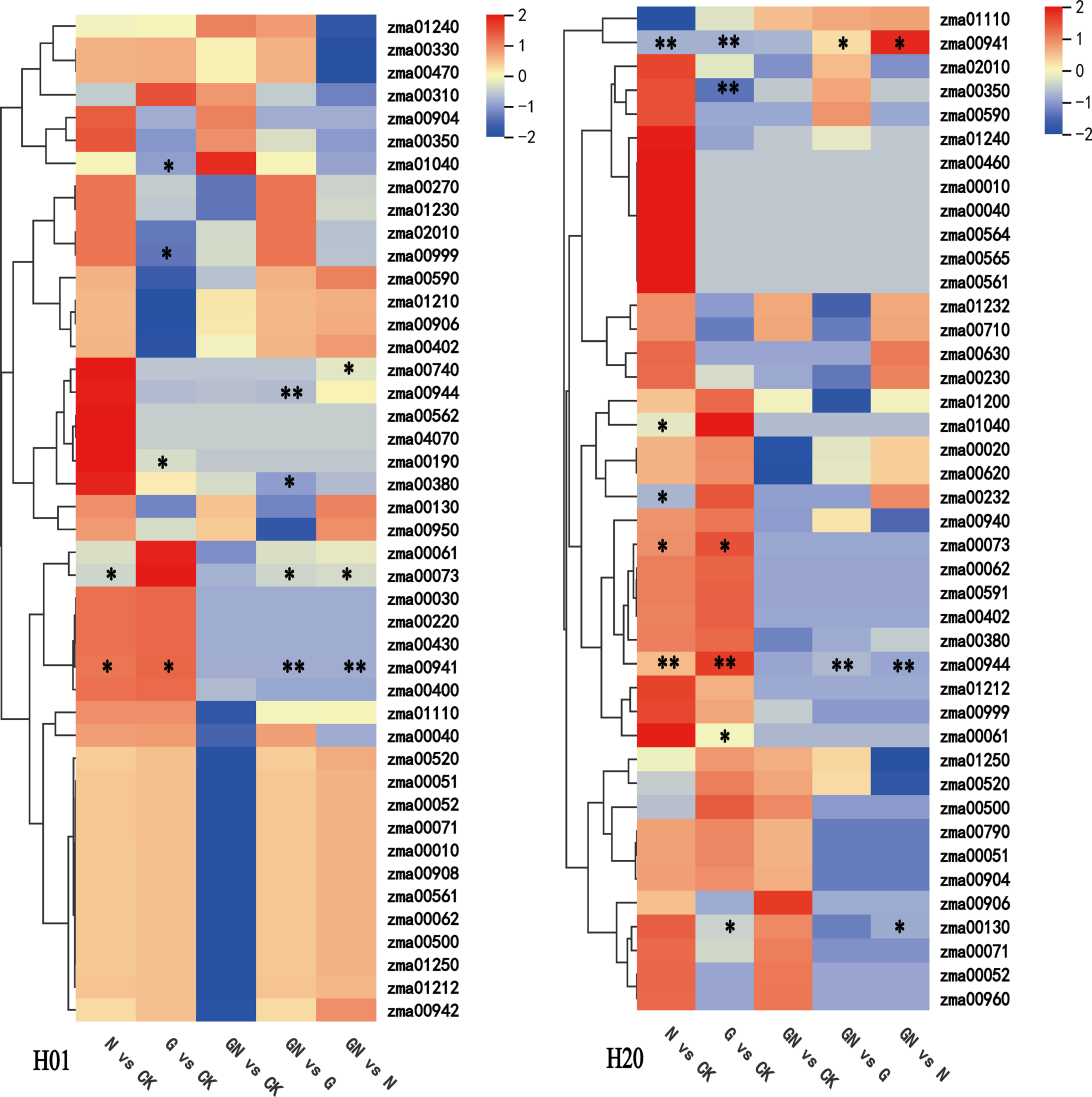
The KEGG pathway analysis of the DEMs. The statistical significance was defined as *P<0.05 or **P<0.01.

A KEGG enrichment pathways analysis was performed to confirm the key metabolic pathways related to the responses of sweet corn to NIF toxicity. NIF toxicity resulted in more metabolites (90) in the H20 inbred lines than in the H01 (70 DEMs) lines. We further identified the key pathways through enrichment and topological analyses. Flavonoid biosynthesis; cutin, suberin, and wax biosynthesis; and benzoxazinoid biosynthesis were the major pathways with the strongest response to NIF (**Supplementary Figures S10A, B**). In the N vs CK and GN vs G groups, flavonoid biosynthesis; cutin, suberin, and wax biosynthesis; and fatty acid biosynthesis were significantly enriched in both the H01 and H20 inbred lines (**Figure 9**).

**Figure 10:**
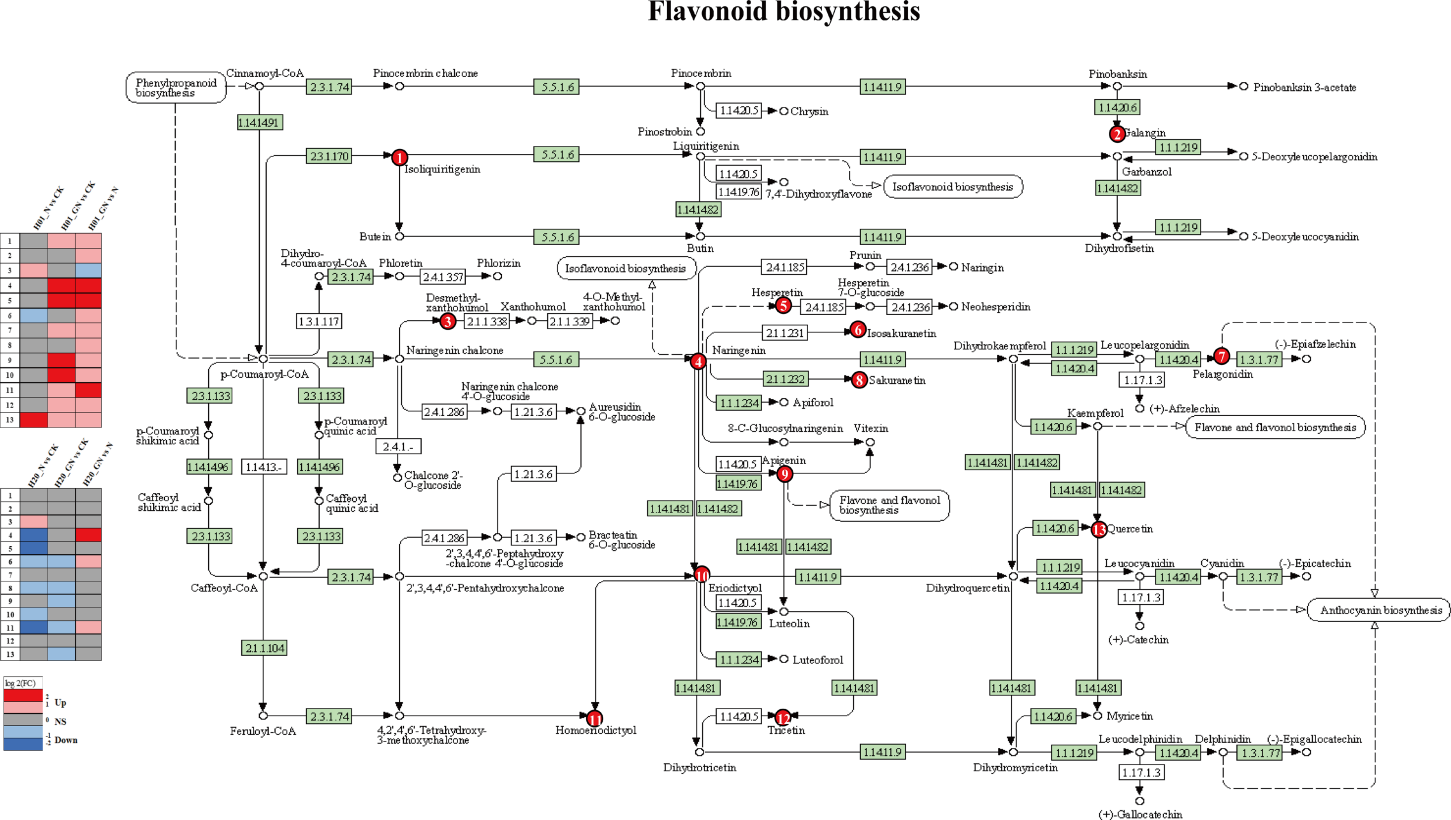
KEGG pathway analysis of flavonoid biosynthesis responses to NIF stress.

We analyzed the metabolites involved in the flavonoid biosynthetic pathway in response to NIF in more detail. We found that treatment with NIF increased two flavonoids, quercetin and desmethylxanthohumol, but decreased isosakuranetin in the H01 sweet corn seedlings. In contrast, NIF decreased six flavonoids, namely, naringenin, hesperetin, homoeriodictyol, isosakuranetin, eriodictyol, and sakuranetin, in the H20 sweet corn seedlings (**Figure 10**). These results indicate that quercetin, hesperetin, naringenin, eriodictyol, isosakuranetin, ssakuranetin, desmethylxanthohumol, and homoeriodictyol in the flavonoid metabolic pathway are involved in the response of sweet corn to NIF (**Figure 10**).

We then analyzed the detoxification pathway used by GO to be a safety agent for NIF. The DEMs that were induced by GO in both the H01 and H20 seedlings treated with NIF were significantly enriched in the pathways of flavonoid biosynthesis and flavone and flavonol biosynthesis (**Supplementary Figures S10C, D**). There was a significant enrichment of the flavonoid biosynthetic pathway in the N vs CK and GN vs N groups (**Figure 9**). In the flavonoid biosynthesis pathway, GN increased the levels of isoliquiritigenin, naringenin, hesperetin, pelargonidin, apigenin, eriodictyol, homoeriodictyol, tricetin, and quercetin compared with the CK. GN also increased the content of galangin, isosakuranetin, and sakuranetin, and GN reduced the content of desmethylxanthohumol compared with the NIF treatment in the H01 seedlings (**Figure 10**). Furthermore, GN increased the contents of naringenin, isosakuranetin, and homoeriodictyol compared with the NIF treatment in the H20 seedlings (**Figure 10**). These results suggest that naringenin, hesperetin, isosakuranetin, and homoeriodictyol may be important metabolites that are involved in the detoxification of GO for NIF in the flavonoid biosynthesis pathway.

## 4 Discussion

### 4.1 Impact of NIF and GO on the SR and physiological indices

NIF toxicity inhibits the SR of sweet corn inbred lines, and the H20 sweet corn inbred lines were more sensitive to herbicides. However, spraying GO alleviated the stress of NIF and improved the growth of seedlings (**Figure 1A**). Studies have shown that spraying GO on leaves can promote plant growth, probably because GO can increase the photosynthetic rate and promote the growth of plants (Chen et al., 2019a). Spraying GO on pigeon peas (*Cajanus cajan*) showed that the GO treatment significantly increased the root length, plant height, and fresh weight of seedlings (Shende et al., 2017). Spraying nanomaterials on rice can increase the seedling biomass and yield of grain (Ahmed et al., 2021). Nanomaterials play an important role in adversity stress. The use of nanomaterials can also enhance the tolerance of rice to salt and its degree of resistance to the disease rice blast caused by *Magnaporthe grisea* (Chen et al., 2017). The use of nanomaterials in wheat can enhance salt tolerance and increase the dry weight of wheat roots, stems, and grains (Muhammad et al., 2023). GO can also reduce the toxicity of cadmium (Cd) to rice seedlings. Our results further confirmed the facilitating effect of GO on the SR of corn seedlings. We found that NIF significantly inhibited the physiological indices of corn seedlings, including the activity of SOD, POD, APX, and CAT and the content of H_2_O_2_ and O_2_^-^. Previous studies have shown that abiotic stress leads to the closure of stomata, which enhances the transfer of O_2_^-^, and SOD rapidly converts O_2_^-^ into H_2_O_2_ (Vaahtera et al., 2014). Previous studies have shown that NIF stress in corn is accompanied by oxidative stress, which significantly inhibits the growth of corn seedlings. Compared with the NIF-tolerant corn inbred lines, the NIF-sensitive corn inbred lines accumulated more H_2_O_2_ and O_2_^-^ (Wang et al., 2018). In this study, compared with the NIF treatment alone, GO reduced these physiological indicators that are harmful to plants, thereby increasing the rate of survival of the sweet corn seedlings (**Figures 1B, C**).

### 4.2 Impact of NIF and GO and photosynthetic parameters and chlorophyll fluorescence parameters

Chlorophyll fluorescence and photosynthetic parameters play an important role in the process of plant photosynthetic activity. In this study, under the NIF treatment, the levels of most parameters were significantly lower than those in the CK, particularly in the H20 corn inbred lines (**Figure 2**). Soluble sugar is the primary product of photosynthesis. Previous studies have shown that the sugar metabolism of corn seedlings is inhibited under the NIF treatment, which affects the growth of seedlings (Xu et al., 2022). The reduction of the photosynthetic parameters in the H20 inbred lines may be owing to the multiple effects of NIF on the leaf tissues and cells after the seedlings had been sprayed. Moreover, the photosynthetic parameters of the H20 seedlings increased significantly after they were sprayed with GN (**Figure 2**). This indicated that GO can reduce the toxicity of NIF and improve its photosynthetic efficiency.

### 4.3 Metabolomic analysis in the different lines of sweet corn

Compared with H01, the H20 seedlings were more sensitive to NIF (**Figure 1A**). A metabolomic analysis revealed that the NIF stress induced 70 DEMs in the H01 seedlings, and it resulted in 90 DEMs in the H20 seedlings. The NIF increased 37 DEMs and decreased 33 DEMs in the H01 seedlings. However, it increased 33 DEMs and decreased 57 DEMs in the H20 seedlings (**Figures 4C, D**). There were 14 common DEMs positively accumulated, and eight mutual DEMs negatively accumulated in both the H01 and H20 inbred lines (**Supplementary Figure S5**). These differential metabolites were primarily derived from the class of flavonoids, phenolics, alkaloids, and fatty acyls (**Supplementary Table S2**). Flavonoid metabolites regulate plant growth and development and play a critical role in biotic and abiotic stress conditions in plants, including stress owing to heavy metals, drought, and salinity (Rao et al., 2020; Kidd et al., 2001; Chen et al., 2019b). In this study, NIF induced a total of 35 flavonoid DEMs (**Supplementary Table S2**). In the NIF-treated H01 seedlings, seven flavonoid metabolites accumulated negatively, while five were positively accumulated. The NIF increased two flavonoid metabolites and decreased 23 flavonoid DEMs in the H20 seedlings. Furthermore, NIF increased one flavonoid (clovin) and reduced four flavonoids (isoscoparin, isosakuranetin, morin, and “2,3,5,7-tetrahydroxyflavone”) in both the H01 and H20 seedlings.

A certain concentration of GO promoted the growth of wheat seedlings and reduced the content of Cd in the rice seedlings (Ren et al., 2020). Our study found that GO increased 52 metabolites and reduced 31 metabolites in the H01 seedlings, but GO increased 30 metabolites and decreased 80 metabolites in the H20 seedlings under the control condition (**Figures 4C, D**). Under the NIF stress conditions, GO increased the accumulation of 81 metabolites but decreased that of 27 metabolites in the H01 seedlings; however, GO increased the levels of 26 metabolites but reduced 40 metabolites in the H20 seedlings (**Figure 4D**). Moreover, the accumulation of petasitenine and chrysoeriol 7-apiosylglucoside decreased, but 10 metabolites were positively accumulated in both the H01 and H20 seedlings treated with GN compared with the NIF treatment (**Supplementary Figure S8**). However, saponarin, sesamol, and hordenine showed an opposite accumulation in the H01 and H20 seedlings when their leaves had been sprayed with GO under NIF stress (**Supplementary Figure S8**). In summary, these results indicated that GO exhibited similar or different mechanisms to enhance the tolerance to NIF in different inbred lines of corn.

Phenolics play an important role in plant growth and development under stress conditions. Phenolic compounds are an important part of the plant cell wall. Moreover, plant growth and resistance to stress require phenolics (Tuladhar et al., 2021). Previous studies have shown that the accumulation of phenolics in corn and rice tended to increase when the plants were stressed (Salama et al., 2015; Minh et al., 2016). In this study, NIF increased the amount of a phenolic (octyl gallate) in both the H01 and H20 seedlings (**Supplementary Table S2**). NIF also increased phenolics, such as oleocanthal, alternariol, and dihydrokavain, in the H20 seedlings (**Supplementary Table S2**). Alkaloids are specific plant protectants against infection by pathogens and attack by other damaging creatures (Abeer et al., 2019). Drought stress in tobacco (*Nicotiana tabacum*) and opium poppy (*Papaver somniferum*) and salt stress in Madagascar periwinkle (*Catharanthus roseus*) increased the accumulation of alkaloids (Cakir and Cebi, 2010). In this study, treatment with NIF increased the amount of an alkaloid (amabiline) in both the H01 and H20 seedlings (**Supplementary Table S2**). Fatty acids are regulators of stress signaling and inducers of oxidative stress (He and Ding, 2020). Treatment with NIF increased three types of fatty acids (octadecanamide, oleamide, and oleic acid) and decreased one fatty acid (suberic acid) in both the H01 and H20 seedlings (**Supplementary Table S2**).

Primary metabolites, such as amino acids, proteins, and nucleic acids, are essential for the maintenance of growth, nutrition, and reproduction in plants. Secondary metabolites, such as flavonoids and alkaloids, also play an important role in drought, salinity, heat, and cold stress (Wu et al., 2023; Brito et al., 2020; Wienkoop et al., 2008). We found that GO restored 59 DEMs in the H01 seedlings and 57 DEMs in the H20 seedlings to normal levels (**Figures 4B, D**). Among them, 16 common DEMs were found in both the H01 and H20 seedlings (**Supplementary Table S3**). These metabolites included flavonoids, alkaloids, fatty acyls, phenolics, lignans, nucleotides, and their derivates. A correlation analysis confirmed that flavonoids, diterpenoids, alkaloids, and amino acids significantly correlated with the SR of sweet corn. Among them, doronine (an alkaloid) was significantly positively correlated with SR (**Figures 7A, B**). Furthermore, flavonoids, alkaloids, amino acids, diterpenoids, fatty acyls, indoles and derivatives, and phenolics were significantly related to the physiological parameters (**Figures 7A, B**). These results indicated that GO promoted the SR of corn seedlings by affecting their physiological indicators. A variety of secondary metabolites were induced by stress, including alkaloids, flavonoids, fatty acyls, and phenolics. These secondary metabolites play an important role in the response of plants to stress (Yang et al., 2018; Yeshi et al., 2022; Tan et al., 2021; Srivastava and Srivastava, 2010). In this study, we found that most of the alkaloids were downregulated, while the fatty acyls were upregulated following induction by NIF. GO restored these metabolites to normal levels in the H01 and H20 seedlings (**Supplementary Table S3**; **Figures 4B, D**). These results indicated that GO affects the SR of sweet corn by regulating the metabolism of alkaloids and fatty acyls.

Flavonoids are secondary metabolites with roles in the mechanisms related to environmental stress and auxin transport. Flavonoids with varying structures have different functions and can provide different protective effects in plants. Flavonoids, as antioxidants, can scavenge the reactive oxygen species (ROS) produced by plants under biotic and abiotic stresses. Under biotic and abiotic stress, flavonoids increase the SR of plants by preventing the production of ROS (Mierziak et al., 2014). In this study, most of the flavonoid metabolites were downregulated in the corn seedlings following treatment with NIF. The results of this study indicate that NIF induces an increase in the synthesis of desmethylxanthohumol and quercetin in the H01 inbred lines. Compared with the NIF treatment alone, GO increased the biosynthesis of isoliquiritigenin, galangin, naringenin, hesperetin, isosakuranetin, pelargonidin, sakuranetin, apigenin, eriodictyol, homoeriodictyol, tricetin, and quercetin. The biosynthesis of 4 and 5 increased significantly. NIF induces an increase in the biosynthesis of desmethylxanthohumol in the H20 inbred lines. Compared with the NIF treatment alone, GO significantly increased the biosynthesis of naringenin, one of the major flavonoids that accumulate when plants are under stress. Naringenin is a major factor that causes different sensitivities to *Phytophthora nicotianae* in resistant and susceptible tobacco (Sun et al., 2022). Hesperetin is a flavonoid that is found widely in plants and has a variety of pharmacological and antioxidant properties. Studies have shown that hesperetin may be a promising functional agent that can prevent heavy metal toxicity (Folake et al., 2024). Our findings are corroborated by that study.

### 4.4 Environmental and practical implications of GO use in agriculture

GO is one of the most representative two-dimensional carbon-based nanomaterials, and has been widely used in agriculture owing to its unparalleled ease of synthesis and excellent compatibility, processability, and versatility (Gao et al., 2022). In recent years, the potential ecological toxicity of GO has attracted widespread attention. Previous studies have shown that GO can cause physiological and biochemical damage to plants, such as delaying seed germination, affecting cell division, changing the biosynthesis of hormones, inducing the accumulation of ROS, and reducing the biosynthesis of chlorophyll (Weng et al., 2022). A high concentration of GO inhibited the length of shoots and fresh weight of wheat (Ren et al., 2020). A total of 200 mg/L GO decreased the main root length and the root/shoot ratio of maize seedlings (Zhao et al., 2021). However, owing to the complexity of the environment, evaluating the impact of GO on plants when it coexists with other pollutants is one of the important tasks to comprehensively evaluate the environmental risks of the spread of GO. The application of GO has a positive role in stress in plants. Previous studies have clearly shown that GO can reduce the accumulation of Cd in rice treated with this heavy metal. The primary reason is that the coexistence of GO and Cd promotes the permeability of the cells of rice roots, thereby accelerating the transport of Cd on the plasma membrane (Li et al., 2020). GO successfully delayed the incidence of tomato vascular wilt and reduced its severity by 29.0% (Diana et al., 2023). In soybean (*Glycine max*), GO can directly increase the plant defense enzymes, hormone content, and drought-related gene expression, thereby improving the resistance of soybean (Zhao et al., 2022). GO enhanced the tolerance of pearl millet (*Pennisetum glaucum*) to salinity stress by modulating its morphological and biophysical traits (Mahmoud and Abdelhameed, 2021). GO improved the saline-alkali parameters, thereby improving the saline-alkali resistance of strawberry (*Fragaria* x *ananassa*) (Malekzadeh et al., 2023). In this study, GO increased the SR and restored the physiological indices and the photosynthetic and chlorophyll fluorescence parameters of sweet corn seedlings under NIF stress (**Figures 1**, **2**). This result indicates that GO can detoxify corn seedlings under NIF stress. In summary, GO can reduce the toxicity of NIF in maize and be used as a safety agent in maize production.

## 5 Conclusion

There are often differences in seedling traits and metabolites of sweet corn inbred lines under NIF and GO treatments, but the relationship between the specific differences in the composition of metabolites and SR should be studied. This study investigated the effects of GO on SR and the response of NIF in different lines of sweet corn seedlings. Spraying GO improved the SR of sweet corn seedlings under the NIF treatment. GO regulated important metabolic pathways, including flavonoids and alkaloids. Moreover, the metabolites involved in this metabolic pathway were significantly correlated with the SR, physiological indices, photosynthetic parameters, and chlorophyll fluorescence parameters in sweet corn. GO restored some NIF-induced differential metabolites to normal levels. This study enabled us to understand how GO regulates metabolites and provides a new perspective to improve the SR of sweet corn seedlings under NIF treatment.

## Data Availability

The original contributions presented in the study are included in the article/**Supplementary Material.** Further inquiries can be directed to the corresponding authors.
