# Supplementary material for: Metabolomic analyses reveal that graphene oxide alleviates nicosulfuron toxicity in sweet corn
Source: Front Plant Sci. 2025 Feb 25;16:1529598. doi: 10.3389/fpls.2025.1529598 (PMC11893866; doi:10.3389/fpls.2025.1529598)
Supplement: Supplementary file 1 [file DataSheet1.pdf]

*Supporting Information for*

# Metabolomic analyses reveal that Graphene oxide alleviate Nicosulfuron toxicity in sweet corn

Jian Wang[1†], Yanbing Wang[2†], Yanli Wang[1*], Xuemei Zhong[3*], Xiuping Wang[1], Xiaohu Lin[1]

[*]Correspondence: yanliwang0720@163.com, xuemei.zhong@163.com

[†]These authors contributed equally to this work

[1] College of Agronomy and Biotechnology, Hebei Normal University of Science and Technology, Qinhuangdao, China; Hebei Key Laboratory of Crop Stress Biology, Qinhuangdao, China,

[2] Institute of Cereal and Oil Crops, Hebei Academy of Agriculture and Forestry Sciences /Hebei Key Laboratory of Crop Genetics and Breeding

[3] College of Agronomy, Shenyang Agricultural University, GA 110866

**Table S1**. The differential expressed metabolites in the seedings of sweet corn H01 and H20 inbred lines

| Group | Compound name | CLASS | VIP | P_VALUE | $log_2$ FC |
|---|---|---|---|---|---|
| H01_GN vs H01_CK | Ganoderic acid F | Triterpenoids | 1.86 | 0.01 | -4.38 |
| H01_GN vs H01_CK | Stevioside | Diterpenoids | 1.71 | 0.03 | -1.91 |
| H01_GN vs H01_CK | Calenduloside E | Triterpenoids | 1.91 | 0.00 | -1.68 |
| H01_GN vs H01_CK | DG | Glycerolipids | 1.69 | 0.04 | -1.65 |
| H01_GN vs H01_CK | Anthranilic acid | Benzoic acid derivatives | 1.74 | 0.03 | -1.54 |
| H01_GN vs H01_CK | Suberic acid | Fatty Acyls | 1.93 | 0.00 | -1.11 |
| H01_GN vs H01_CK | Yohimbic acid monohydrate | Alkaloids | 1.67 | 0.04 | -1.07 |
| H01_GN vs H01_CK | Hepoxilin B3 | Fatty Acyls | 1.68 | 0.03 | -0.95 |
| H01_GN vs H01_CK | Methylisopelletierine | Alkaloids | 1.89 | 0.00 | -0.90 |
| H01_GN vs H01_CK | "Malvidin 3,5-diglucoside (Malvin)" | Flavonoids | 1.72 | 0.04 | -0.73 |
| H01_GN vs H01_CK | Doronine | Alkaloids | 1.67 | 0.03 | -0.71 |
| H01_GN vs H01_CK | Protocatechuic acid | Phenols | 1.68 | 0.04 | -0.69 |
| H01_GN vs H01_CK | Hordenine | Alkaloids | 1.69 | 0.04 | 0.59 |
| H01_GN vs H01_CK | Isoquercitrin | flavonoids | 1.65 | 0.05 | 0.59 |
| H01_GN vs H01_CK | 7-Methylxanthine | Nucleotide and its derivates | 1.68 | 0.04 | 0.60 |
| H01_GN vs H01_CK | (-)-Anonaine | Alkaloids | 1.82 | 0.01 | 0.61 |
| H01_GN vs H01_CK | Gossypetin | Flavonoids | 1.78 | 0.02 | 0.61 |
| H01_GN vs H01_CK | Benzocaine | Benzene and substituted derivatives | 1.82 | 0.01 | 0.62 |
| H01_GN vs H01_CK | Lonicerin | Flavonoids | 1.81 | 0.01 | 0.62 |
| H01_GN vs H01_CK | Undecanolactone | Miscellaneous | 1.75 | 0.02 | 0.64 |
| H01_GN vs H01_CK | vitamin K2 | Vitamins | 1.70 | 0.03 | 0.65 |

| | | | | | |
|---|---|---|---|---|---|
| H01_GN vs H01_CK | "(R)-2-Hydroxy-2H-1,4-benzoxazin-3(4H)-one" | Benzoxazines | 1.91 | 0.00 | 0.68 |
| H01_GN vs H01_CK | Formylanthranilic acid | Benzene and substituted derivatives | 1.77 | 0.02 | 0.71 |
| H01_GN vs H01_CK | 5-Aminovaleric acid | Amino acid and derivatives | 1.80 | 0.01 | 0.73 |
| H01_GN vs H01_CK | L-Gulose | Carbohydrates | 1.77 | 0.03 | 0.74 |
| H01_GN vs H01_CK | "3,4-Dihydroxybenzaldehyde" | Phenols | 1.72 | 0.03 | 0.75 |
| H01_GN vs H01_CK | Dalbergioidin | Flavonoids | 1.86 | 0.01 | 0.79 |
| H01_GN vs H01_CK | Guaiacol | Phenols | 1.98 | 0.00 | 0.80 |
| H01_GN vs H01_CK | Sesamol | Phenols | 1.74 | 0.03 | 0.83 |
| H01_GN vs H01_CK | N-(p-Hydroxyphenethyl)actinidine | Phenols | 1.91 | 0.01 | 0.87 |
| H01_GN vs H01_CK | isoliquiritigenin | flavonoids | 1.91 | 0.00 | 0.96 |
| H01_GN vs H01_CK | Clovin | Flavonoids | 1.83 | 0.01 | 1.01 |
| H01_GN vs H01_CK | N-D-Glucosylarylamine | glucose | 1.77 | 0.02 | 1.09 |
| H01_GN vs H01_CK | Betulinic acid | Triterpenoids | 1.90 | 0.01 | 1.11 |
| H01_GN vs H01_CK | Cathinone | Alkaloids | 1.70 | 0.04 | 1.12 |
| H01_GN vs H01_CK | 3-Indolebutyric acid | phytohormone | 1.66 | 0.04 | 1.13 |
| H01_GN vs H01_CK | Broussonin C | Phenols | 1.78 | 0.03 | 1.14 |
| H01_GN vs H01_CK | Desoxypeganine | Alkaloids | 1.92 | 0.00 | 1.16 |
| H01_GN vs H01_CK | 4-Pyridoxic acid | Pyridine derivatives | 1.77 | 0.02 | 1.19 |
| H01_GN vs H01_CK | Pelargonidin | Flavonoids | 1.79 | 0.02 | 1.22 |
| H01_GN vs H01_CK | Testosterone | Steroids | 1.63 | 0.04 | 1.25 |
| H01_GN vs H01_CK | Picrotoxinin | Sesquiterpenoids | 1.81 | 0.01 | 1.28 |
| H01_GN vs H01_CK | Withaferin A | Steroids and steroid derivatives | 1.83 | 0.01 | 1.30 |
| H01_GN vs H01_CK | Pterosin D | Sesquiterpenoids | 1.79 | 0.02 | 1.38 |
| H01_GN vs H01_CK | Ganoderic acid L | Alkaloids | 1.68 | 0.03 | 1.43 |
| H01_GN vs H01_CK | "Adenosine 2,3-cyclic phosphate" | Purine nucleotides | 1.78 | 0.01 | 1.48 |

| | | | | | |
|---|---|---|---|---|---|
| H01_GN vs H01_CK | Lutein | Prenol lipids | 1.89 | 0.01 | 1.58 |
| H01_GN vs H01_CK | Tricetin | Flavonoids | 1.76 | 0.02 | 1.73 |
| H01_GN vs H01_CK | Homoeriodictyol | Flavonoids | 1.94 | 0.00 | 1.78 |
| H01_GN vs H01_CK | L-Isoleucine | Amino acid and derivatives | 1.94 | 0.00 | 1.80 |
| H01_GN vs H01_CK | Quercetin | flavonoids | 1.83 | 0.01 | 1.91 |
| H01_GN vs H01_CK | Deoxyguanosine | Nucleotide and its derivates | 1.84 | 0.01 | 2.06 |
| H01_GN vs H01_CK | Loganin | Terpene | 1.82 | 0.01 | 2.06 |
| H01_GN vs H01_CK | 6-Methoxymellein | Benzopyrans | 1.73 | 0.03 | 2.11 |
| H01_GN vs H01_CK | Oleocanthal | Phenols | 1.89 | 0.01 | 2.13 |
| H01_GN vs H01_CK | Eriodictyol | flavonoids | 1.96 | 0.00 | 2.22 |
| H01_GN vs H01_CK | Arachidonic acid | Fatty Acyls | 1.72 | 0.04 | 2.31 |
| H01_GN vs H01_CK | Darlingine | Alkaloids | 1.67 | 0.05 | 2.45 |
| H01_GN vs H01_CK | Apigenin | flavonoids | 1.88 | 0.01 | 2.46 |
| H01_GN vs H01_CK | Naringenin | flavonoids | 1.97 | 0.00 | 2.71 |
| H01_GN vs H01_CK | Hesperetin | flavonoids | 1.96 | 0.00 | 2.75 |
| H01_GN vs H01_CK | Bruceine D | Diterpenoids | 1.98 | 0.00 | 3.23 |
| H01_GN vs H01_CK | Glycitein | Flavonoids | 1.85 | 0.01 | 3.56 |
| H01_GN vs H01_G | 7-(4-Hydroxyphenyl)-1-phenyl-4-hepten-3-one | Phenols | 1.92 | 0.00 | -3.13 |
| H01_GN vs H01_G | Glycerophosphocholine | Cholines | 1.79 | 0.01 | -3.02 |
| H01_GN vs H01_G | Octyl Gallate | Phenols | 1.87 | 0.00 | -2.77 |
| H01_GN vs H01_G | Panaxynol | Miscellaneous | 1.72 | 0.01 | -2.77 |
| H01_GN vs H01_G | Bergamotine | Coumarins | 1.78 | 0.01 | -2.55 |
| H01_GN vs H01_G | 4-Hydroxyphenylacetylglutamic acid | Organic acids | 1.91 | 0.00 | -2.52 |
| H01_GN vs H01_G | Octadecanamide | Fatty Acyls | 1.92 | 0.00 | -2.50 |
| H01_GN vs H01_G | Palmitic acid | Lipids | 1.69 | 0.02 | -2.48 |

| | | | | | |
|---|---|---|---|---|---|
| H01_GN vs H01_G | "8,9-DiHETrE" | Fatty Acyls | 1.85 | 0.00 | -2.46 |
| H01_GN vs H01_G | Oleic acid | Fatty Acyls | 1.86 | 0.00 | -2.39 |
| H01_GN vs H01_G | Artemisinin | Sesquiterpenoids | 1.90 | 0.00 | -2.39 |
| H01_GN vs H01_G | Miltirone | Diterpenoids | 1.88 | 0.00 | -2.35 |
| H01_GN vs H01_G | Amabiline | Alkaloids | 1.87 | 0.00 | -2.32 |
| H01_GN vs H01_G | Glucose 1-phosphate | Organooxygen compounds | 1.91 | 0.00 | -2.18 |
| H01_GN vs H01_G | Oleamide | Fatty Acyls | 1.84 | 0.00 | -2.15 |
| H01_GN vs H01_G | Mannose 6-phosphate | Organooxygen compounds | 1.90 | 0.00 | -1.93 |
| H01_GN vs H01_G | Desmethylxanthohumol | Chalcones | 1.76 | 0.01 | -1.90 |
| H01_GN vs H01_G | Curzerene | Sesquiterpenoids | 1.74 | 0.01 | -1.61 |
| H01_GN vs H01_G | Mitraphylline | Alkaloids | 1.65 | 0.03 | -1.51 |
| H01_GN vs H01_G | Moracin C | Phenols | 1.83 | 0.01 | -1.25 |
| H01_GN vs H01_G | "ent-16beta,17-dihydroxy-9(11)-kauren-19-oic acid" | Diterpenoids | 1.69 | 0.02 | -1.20 |
| H01_GN vs H01_G | 5-Aminovaleric acid | Amino acid and derivatives | 1.79 | 0.01 | -1.09 |
| H01_GN vs H01_G | Phillyrin | Phenylpropanoids | 1.61 | 0.04 | -1.02 |
| H01_GN vs H01_G | Yohimbic acid monohydrate | Alkaloids | 1.61 | 0.03 | -0.92 |
| H01_GN vs H01_G | Chrysoeriol 7-apiosylglucoside | Flavonoids | 1.81 | 0.00 | -0.72 |
| H01_GN vs H01_G | Cinnamyl cinnamate | Phenylpropanoids | 1.77 | 0.01 | -0.67 |
| H01_GN vs H01_G | Formylanthranilic acid | Benzene and substituted derivatives | 1.88 | 0.00 | 0.60 |
| H01_GN vs H01_G | (-)-Salsoline | Tetrahydroisoquinolines | 1.64 | 0.04 | 0.61 |
| H01_GN vs H01_G | Symlandine | Alkaloids | 1.77 | 0.01 | 0.61 |
| H01_GN vs H01_G | 3-Hydroxyphenylacetic acid | Phenols | 1.58 | 0.04 | 0.61 |
| H01_GN vs H01_G | Ponasterone A | Steroids | 1.63 | 0.03 | 0.62 |
| H01_GN vs H01_G | 2-Picolinic acid | Organic acids | 1.77 | 0.01 | 0.64 |
| H01_GN vs H01_G | Diosmin | Flavonoids | 1.65 | 0.03 | 0.67 |

| | | | | | |
|---|---|---|---|---|---|
| H01_GN vs H01_G | 5-S-Methyl-5-thioadenosine | Nucleotide and its derivates | 1.75 | 0.01 | 0.71 |
| H01_GN vs H01_G | "(R)-2-Hydroxy-2H-1,4-benzoxazin-3(4H)-one" | Benzoxazines | 1.85 | 0.00 | 0.73 |
| H01_GN vs H01_G | Melatonin | Alkaloids | 1.70 | 0.02 | 0.74 |
| H01_GN vs H01_G | Rutin | flavonoids | 1.77 | 0.01 | 0.76 |
| H01_GN vs H01_G | Kaempferol-3-O-rutinoside | flavonoids | 1.81 | 0.01 | 0.79 |
| H01_GN vs H01_G | Jasmonic acid | Fatty Acyls | 1.67 | 0.03 | 0.80 |
| H01_GN vs H01_G | Tripdiolide | Diterpenoids | 1.66 | 0.03 | 0.81 |
| H01_GN vs H01_G | (-)-Anonaine | Alkaloids | 1.58 | 0.05 | 0.83 |
| H01_GN vs H01_G | 7-Methylguanine | Imidazopyrimidines | 1.66 | 0.03 | 0.85 |
| H01_GN vs H01_G | Xanthohumol | Flavanone | 1.63 | 0.03 | 0.86 |
| H01_GN vs H01_G | Lonicerin | Flavonoids | 1.73 | 0.01 | 0.86 |
| H01_GN vs H01_G | Xanthurenic acid | Quinolines and derivatives | 1.80 | 0.01 | 0.90 |
| H01_GN vs H01_G | Sinapine | Cholines | 1.65 | 0.02 | 0.90 |
| H01_GN vs H01_G | Medicagenic acid | Triterpenoids | 1.61 | 0.05 | 0.92 |
| H01_GN vs H01_G | Sinapyl alcohol | Hydroxycinnamoyl derivatives | 1.61 | 0.05 | 0.93 |
| H01_GN vs H01_G | Nordihydroguaiaretic acid | Lignans | 1.76 | 0.01 | 0.94 |
| H01_GN vs H01_G | Lutein | Prenol lipids | 1.66 | 0.03 | 0.94 |
| H01_GN vs H01_G | Picrotoxinin | Sesquiterpenoids | 1.77 | 0.01 | 0.94 |
| H01_GN vs H01_G | Dalbergioidin | Flavonoids | 1.73 | 0.02 | 0.95 |
| H01_GN vs H01_G | "5-Tricosyl-1,3-benzenediol" | Phenols | 1.78 | 0.01 | 0.95 |
| H01_GN vs H01_G | Carbofuran | Coumarans | 1.56 | 0.05 | 0.99 |
| H01_GN vs H01_G | Rhein | Anthraquinones | 1.67 | 0.02 | 1.00 |
| H01_GN vs H01_G | Isoquercitrin | flavonoids | 1.85 | 0.00 | 1.05 |
| H01_GN vs H01_G | Glucoiberin | Organooxygen compounds | 1.62 | 0.03 | 1.06 |
| H01_GN vs H01_G | Loganin | Terpene | 1.62 | 0.04 | 1.08 |

| | | | | | |
|---|---|---|---|---|---|
| H01_GN vs H01_G | Rebaudioside B | Diterpenoids | 1.58 | 0.04 | 1.08 |
| H01_GN vs H01_G | 1-Caffeoylquinic acid | Phenylpropanoids | 1.72 | 0.02 | 1.16 |
| H01_GN vs H01_G | Sakuranetin | Flavonoids | 1.63 | 0.04 | 1.17 |
| H01_GN vs H01_G | L-Pipecolic acid | Amino acid and derivatives | 1.83 | 0.00 | 1.19 |
| H01_GN vs H01_G | Isoeugenol | Phenols | 1.74 | 0.01 | 1.20 |
| H01_GN vs H01_G | Gossypetin | Flavonoids | 1.76 | 0.01 | 1.24 |
| H01_GN vs H01_G | N-D-Glucosylarylamine | glucose | 1.73 | 0.01 | 1.30 |
| H01_GN vs H01_G | Guaiacol | Phenols | 1.83 | 0.00 | 1.30 |
| H01_GN vs H01_G | Cathinone | Alkaloids | 1.72 | 0.02 | 1.37 |
| H01_GN vs H01_G | Isosakuranetin | Flavonoids | 1.76 | 0.01 | 1.44 |
| H01_GN vs H01_G | Suberic acid | Fatty Acyls | 1.84 | 0.01 | 1.50 |
| H01_GN vs H01_G | Quercetin-3-O-sophoroside | Flavonoids | 1.66 | 0.03 | 1.60 |
| H01_GN vs H01_G | N-Acetyl-L-phenylalanine | Amino acid and derivatives | 1.66 | 0.03 | 1.62 |
| H01_GN vs H01_G | (-)-Sativan | Flavonoids | 1.58 | 0.04 | 1.65 |
| H01_GN vs H01_G | Withaferin A | Steroids and steroid derivatives | 1.83 | 0.00 | 1.68 |
| H01_GN vs H01_G | Reserpine | Alkaloids | 1.88 | 0.00 | 1.70 |
| H01_GN vs H01_G | Benzocaine | Benzene and substituted derivatives | 1.88 | 0.00 | 1.71 |
| H01_GN vs H01_G | Picrasin B | Diterpenoids | 1.74 | 0.01 | 1.71 |
| H01_GN vs H01_G | "2,3,5,7-Tetrahydroxyflavone" | Flavonoids | 1.75 | 0.01 | 1.76 |
| H01_GN vs H01_G | Naringenin | flavonoids | 1.86 | 0.00 | 1.91 |
| H01_GN vs H01_G | Pelargonidin | Flavonoids | 1.77 | 0.01 | 1.92 |
| H01_GN vs H01_G | L-Isoleucine | Amino acid and derivatives | 1.88 | 0.00 | 1.94 |
| H01_GN vs H01_G | 6-Methoxymellein | Benzopyrans | 1.59 | 0.04 | 1.97 |
| H01_GN vs H01_G | Piperlonguminine | Alkaloids | 1.90 | 0.00 | 2.00 |
| H01_GN vs H01_G | Nandrolone | Steroids and steroid derivatives | 1.91 | 0.00 | 2.06 |

| | | | | | |
|---|---|---|---|---|---|
| H01_GN vs H01_G | Homoeriodictyol | Flavonoids | 1.90 | 0.00 | 2.08 |
| H01_GN vs H01_G | Eriodictyol | flavonoids | 1.85 | 0.00 | 2.08 |
| H01_GN vs H01_G | Samidin | Coumarins | 1.79 | 0.01 | 2.14 |
| H01_GN vs H01_G | Apigenin | flavonoids | 1.80 | 0.01 | 2.20 |
| H01_GN vs H01_G | DL-Tyrosine | Alkaloids | 1.62 | 0.03 | 2.20 |
| H01_GN vs H01_G | 2-(Methylamino)benzoic acid | Benzene and substituted derivatives | 1.64 | 0.03 | 2.21 |
| H01_GN vs H01_G | Norbixin | Prenol lipids | 1.75 | 0.01 | 2.29 |
| H01_GN vs H01_G | Hepoxilin B3 | Fatty Acyls | 1.83 | 0.00 | 2.31 |
| H01_GN vs H01_G | Glycitin | Flavonoids | 1.92 | 0.00 | 2.52 |
| H01_GN vs H01_G | Glycitein | Flavonoids | 1.77 | 0.01 | 3.21 |
| H01_GN vs H01_G | Hesperetin | flavonoids | 1.88 | 0.00 | 3.40 |
| H01_GN vs H01_N | Petasitenine | Alkaloids | 1.79 | 0.00 | -4.85 |
| H01_GN vs H01_N | Aristolochic acid A | Miscellaneous | 1.70 | 0.01 | -3.88 |
| H01_GN vs H01_N | Glycerophosphocholine | Cholines | 1.84 | 0.00 | -3.02 |
| H01_GN vs H01_N | 7-(4-Hydroxyphenyl)-1-phenyl-4-hepten-3-one | Phenols | 1.74 | 0.01 | -2.84 |
| H01_GN vs H01_N | Octyl Gallate | Phenols | 1.83 | 0.00 | -2.72 |
| H01_GN vs H01_N | Panaxynol | Miscellaneous | 1.74 | 0.01 | -2.49 |
| H01_GN vs H01_N | 4-Hydroxyphenylacetylglutamic acid | Organic acids | 1.86 | 0.00 | -2.49 |
| H01_GN vs H01_N | Oleic acid | Fatty Acyls | 1.86 | 0.00 | -2.48 |
| H01_GN vs H01_N | Artemisinin | Sesquiterpenoids | 1.78 | 0.00 | -2.45 |
| H01_GN vs H01_N | Palmitic acid | Lipids | 1.75 | 0.01 | -2.41 |
| H01_GN vs H01_N | "8,9-DiHETrE" | Fatty Acyls | 1.85 | 0.00 | -2.38 |
| H01_GN vs H01_N | Miltirone | Diterpenoids | 1.85 | 0.00 | -2.29 |
| H01_GN vs H01_N | Amabiline | Alkaloids | 1.86 | 0.00 | -2.28 |
| H01_GN vs H01_N | Octadecanamide | Fatty Acyls | 1.84 | 0.00 | -2.23 |

| | | | | | |
|---|---|---|---|---|---|
| H01_GN vs H01_N | Glucose 1-phosphate | Organooxygen compounds | 1.77 | 0.00 | -2.21 |
| H01_GN vs H01_N | Bergamotine | Coumarins | 1.86 | 0.00 | -2.08 |
| H01_GN vs H01_N | Oleamide | Fatty Acyls | 1.82 | 0.00 | -1.93 |
| H01_GN vs H01_N | Desmethylxanthohumol | Chalcones | 1.84 | 0.00 | -1.88 |
| H01_GN vs H01_N | Mannose 6-phosphate | Organooxygen compounds | 1.85 | 0.00 | -1.80 |
| H01_GN vs H01_N | Symlandine | Alkaloids | 1.76 | 0.00 | -1.43 |
| H01_GN vs H01_N | Methylisopelletierine | Alkaloids | 1.54 | 0.04 | -1.13 |
| H01_GN vs H01_N | Mitraphylline | Alkaloids | 1.64 | 0.02 | -1.08 |
| H01_GN vs H01_N | Moracin C | Phenols | 1.70 | 0.01 | -0.94 |
| H01_GN vs H01_N | Cyanidin-3-O-rhamnoside chloride | Flavonoids | 1.67 | 0.02 | -0.92 |
| H01_GN vs H01_N | Chrysoeriol 7-apiosylglucoside | Flavonoids | 1.65 | 0.02 | -0.74 |
| H01_GN vs H01_N | Calenduloside E | Triterpenoids | 1.72 | 0.01 | -0.73 |
| H01_GN vs H01_N | Phosphonoacetate | Organic acids | 1.61 | 0.03 | -0.68 |
| H01_GN vs H01_N | Hordenine | Alkaloids | 1.60 | 0.03 | 0.60 |
| H01_GN vs H01_N | Desoxypeganine | Alkaloids | 1.80 | 0.00 | 0.61 |
| H01_GN vs H01_N | 1H-Indole-3-carboxylic acid | Alkaloids | 1.64 | 0.02 | 0.66 |
| H01_GN vs H01_N | L-Arabitol | Organooxygen compounds | 1.50 | 0.05 | 0.68 |
| H01_GN vs H01_N | "3,4-Dihydroxybenzaldehyde" | Phenols | 1.66 | 0.02 | 0.70 |
| H01_GN vs H01_N | Daidzein | Flavonoids | 1.67 | 0.02 | 0.70 |
| H01_GN vs H01_N | vitamin K2 | Vitamins | 1.69 | 0.01 | 0.72 |
| H01_GN vs H01_N | Suberic acid | Fatty Acyls | 1.58 | 0.04 | 0.73 |
| H01_GN vs H01_N | Podophyllotoxinone | Lignans | 1.71 | 0.01 | 0.74 |
| H01_GN vs H01_N | 5-S-Methyl-5-thioadenosine | Nucleotide and its derivates | 1.84 | 0.00 | 0.74 |
| H01_GN vs H01_N | (-)-Salsoline | Tetrahydroisoquinolines | 1.61 | 0.03 | 0.75 |
| H01_GN vs H01_N | "5-Tricosyl-1,3-benzenediol" | Phenols | 1.68 | 0.01 | 0.77 |

| | | | | | |
|---|---|---|---|---|---|
| H01_GN vs H01_N | Carbofuran | Coumarans | 1.55 | 0.04 | 0.77 |
| H01_GN vs H01_N | isoliquiritigenin | flavonoids | 1.74 | 0.01 | 0.79 |
| H01_GN vs H01_N | "(-)-3-(3,4-Dihydroxyphenyl)-2-methylalanine" | Amino acid and its derivatives | 1.56 | 0.05 | 0.87 |
| H01_GN vs H01_N | Gossypetin | Flavonoids | 1.65 | 0.02 | 0.88 |
| H01_GN vs H01_N | Sesamol | Phenols | 1.70 | 0.01 | 0.89 |
| H01_GN vs H01_N | Garbanzol | Flavonoids | 1.53 | 0.05 | 0.91 |
| H01_GN vs H01_N | Dimethylbenzimidazole | Benzimidazoles | 1.52 | 0.05 | 0.92 |
| H01_GN vs H01_N | 3-Hydroxyphenylacetic acid | Phenols | 1.62 | 0.02 | 0.92 |
| H01_GN vs H01_N | Melatonin | Alkaloids | 1.56 | 0.04 | 0.96 |
| H01_GN vs H01_N | Riboflavine | Vitamins | 1.83 | 0.00 | 0.96 |
| H01_GN vs H01_N | Dehydronuciferine | Alkaloids | 1.79 | 0.00 | 0.97 |
| H01_GN vs H01_N | "cis-3-(Carboxy-ethyl)-3,5-cyclo-hexadiene-1,2-diol" | organic ester | 1.58 | 0.03 | 0.98 |
| H01_GN vs H01_N | Betulinic acid | Triterpenoids | 1.75 | 0.01 | 1.01 |
| H01_GN vs H01_N | N-(p-Hydroxyphenethyl)actinidine | Phenols | 1.74 | 0.01 | 1.02 |
| H01_GN vs H01_N | Benzocaine | Benzene and substituted derivatives | 1.78 | 0.00 | 1.03 |
| H01_GN vs H01_N | Withaferin A | Steroids and steroid derivatives | 1.76 | 0.00 | 1.04 |
| H01_GN vs H01_N | Quercetin-3-O-glucuronide | Flavonoids | 1.58 | 0.03 | 1.04 |
| H01_GN vs H01_N | Pelargonidin | Flavonoids | 1.56 | 0.04 | 1.05 |
| H01_GN vs H01_N | Deoxypodophyllotoxin | Lignans | 1.62 | 0.02 | 1.05 |
| H01_GN vs H01_N | "trans-3,5-Dimethoxy-4-hydroxy cinnamaldehydee" | Phenylpropanoids | 1.63 | 0.02 | 1.06 |
| H01_GN vs H01_N | Tripdiolide | Diterpenoids | 1.70 | 0.01 | 1.07 |
| H01_GN vs H01_N | Euxanthone | Xanthones | 1.55 | 0.04 | 1.07 |
| H01_GN vs H01_N | L-Isoleucine | Amino acid and derivatives | 1.70 | 0.01 | 1.07 |
| H01_GN vs H01_N | Kaempferol-3-O-rutinoside | flavonoids | 1.83 | 0.00 | 1.07 |
| H01_GN vs H01_N | Taxifolin | flavonoids | 1.77 | 0.01 | 1.08 |

| | | | | | |
|---|---|---|---|---|---|
| H01_GN vs H01_N | Sinapyl alcohol | Hydroxycinnamoyl derivatives | 1.59 | 0.03 | 1.08 |
| H01_GN vs H01_N | 11-Keto-beta-boswellic acid | Triterpenoids | 1.70 | 0.01 | 1.10 |
| H01_GN vs H01_N | Dalbergioidin | Flavonoids | 1.78 | 0.00 | 1.11 |
| H01_GN vs H01_N | Deoxyguanosine | Nucleotide and its derivates | 1.66 | 0.02 | 1.12 |
| H01_GN vs H01_N | O-Succinyl-L-homoserine | Amino acid and derivatives | 1.54 | 0.05 | 1.13 |
| H01_GN vs H01_N | Tricetin | Flavonoids | 1.55 | 0.04 | 1.15 |
| H01_GN vs H01_N | Glucoiberin | Organooxygen compounds | 1.56 | 0.04 | 1.16 |
| H01_GN vs H01_N | 7-Methylguanine | Imidazopyrimidines | 1.76 | 0.01 | 1.21 |
| H01_GN vs H01_N | Piperlonguminine | Alkaloids | 1.82 | 0.00 | 1.23 |
| H01_GN vs H01_N | Isoscoparin | Flavonoids | 1.81 | 0.00 | 1.24 |
| H01_GN vs H01_N | Formylanthranilic acid | Benzene and substituted derivatives | 1.85 | 0.00 | 1.25 |
| H01_GN vs H01_N | Stearidonic acid | Fatty Acyls | 1.59 | 0.03 | 1.27 |
| H01_GN vs H01_N | Yatein | Lignans | 1.56 | 0.04 | 1.28 |
| H01_GN vs H01_N | Atranorin | Phenols | 1.54 | 0.05 | 1.28 |
| H01_GN vs H01_N | Cascaroside A | Anthraquinones | 1.60 | 0.03 | 1.32 |
| H01_GN vs H01_N | "2,3,5,7-Tetrahydroxyflavone" | Flavonoids | 1.81 | 0.00 | 1.33 |
| H01_GN vs H01_N | N-D-Glucosylarylamine | glucose | 1.65 | 0.02 | 1.38 |
| H01_GN vs H01_N | Nandrolone | Steroids and steroid derivatives | 1.85 | 0.00 | 1.40 |
| H01_GN vs H01_N | Guaiacol | Phenols | 1.85 | 0.00 | 1.42 |
| H01_GN vs H01_N | 2-Picolinic acid | Organic acids | 1.64 | 0.02 | 1.43 |
| H01_GN vs H01_N | Cinnamyl cinnamate | Phenylpropanoids | 1.73 | 0.01 | 1.48 |
| H01_GN vs H01_N | "2,6-Dihydroxy 4-methoxydihydrochalcone" | Chalcones | 1.66 | 0.02 | 1.49 |
| H01_GN vs H01_N | Isosakuranetin | Flavonoids | 1.74 | 0.01 | 1.51 |
| H01_GN vs H01_N | "(R)-2-Hydroxy-2H-1,4-benzoxazin-3(4H)-one" | Benzoxazines | 1.84 | 0.00 | 1.54 |
| H01_GN vs H01_N | Loganin | Terpene | 1.60 | 0.03 | 1.56 |

| | | | | | |
|---|---|---|---|---|---|
| H01_GN vs H01_N | Psychosine | Sphingolipids | 1.59 | 0.03 | 1.58 |
| H01_GN vs H01_N | Eriodictyol | flavonoids | 1.82 | 0.00 | 1.58 |
| H01_GN vs H01_N | Medicagenic acid | Triterpenoids | 1.77 | 0.01 | 1.61 |
| H01_GN vs H01_N | Apigenin | flavonoids | 1.66 | 0.02 | 1.67 |
| H01_GN vs H01_N | Mesembrine | Alkaloids | 1.72 | 0.01 | 1.68 |
| H01_GN vs H01_N | Blumeatin | Flavonoids | 1.57 | 0.03 | 1.72 |
| H01_GN vs H01_N | Lutein | Prenol lipids | 1.71 | 0.01 | 1.73 |
| H01_GN vs H01_N | Norbixin | Prenol lipids | 1.65 | 0.02 | 1.75 |
| H01_GN vs H01_N | 1-Acetoxychavicol acetate | Phenols | 1.53 | 0.05 | 1.77 |
| H01_GN vs H01_N | 3-Indolebutyric acid | phytohormone | 1.63 | 0.02 | 1.78 |
| H01_GN vs H01_N | Saponarin | Flavonoids | 1.61 | 0.02 | 1.79 |
| H01_GN vs H01_N | Dihydrojasmonic Acid | phytohormone | 1.57 | 0.04 | 1.88 |
| H01_GN vs H01_N | Quercetin-3-O-sophoroside | Flavonoids | 1.70 | 0.01 | 1.92 |
| H01_GN vs H01_N | Oleocanthal | Phenols | 1.74 | 0.01 | 1.95 |
| H01_GN vs H01_N | Arachidonic acid | Fatty Acyls | 1.58 | 0.04 | 2.28 |
| H01_GN vs H01_N | Glycitein | Flavonoids | 1.72 | 0.01 | 2.93 |
| H01_GN vs H01_N | Hesperetin | flavonoids | 1.85 | 0.00 | 3.05 |
| H01_GN vs H01_N | Naringenin | flavonoids | 1.84 | 0.00 | 3.18 |
| H01_GN vs H01_N | Homoeriodictyol | Flavonoids | 1.84 | 0.00 | 3.22 |
| H01_G vs H01_CK | Alpha-Obscurine | Alkaloids | 1.59 | 0.05 | -9.01 |
| H01_G vs H01_CK | Hepoxilin B3 | Fatty Acyls | 1.86 | 0.00 | -3.26 |
| H01_G vs H01_CK | Suberic acid | Fatty Acyls | 1.93 | 0.00 | -2.61 |
| H01_G vs H01_CK | Glycitin | Flavonoids | 1.66 | 0.03 | -2.48 |
| H01_G vs H01_CK | Samidin | Coumarins | 1.89 | 0.00 | -2.42 |
| H01_G vs H01_CK | Anthranilic acid | Benzoic acid derivatives | 1.74 | 0.02 | -2.03 |

| | | | | | |
|---|---|---|---|---|---|
| H01_G vs H01_CK | 4-Hydroxybenzaldehyde | Phenols | 1.77 | 0.01 | -2.02 |
| H01_G vs H01_CK | Reserpine | Alkaloids | 1.89 | 0.00 | -1.89 |
| H01_G vs H01_CK | Matairesinol | Lignans | 1.68 | 0.03 | -1.75 |
| H01_G vs H01_CK | 6-Phosphogluconic acid | Organooxygen compounds | 1.61 | 0.04 | -1.69 |
| H01_G vs H01_CK | Beta-Sitosterol | Steroids and steroid derivatives | 1.81 | 0.01 | -1.67 |
| H01_G vs H01_CK | Norbixin | Prenol lipids | 1.89 | 0.00 | -1.50 |
| H01_G vs H01_CK | Homoorientin | flavonoids | 1.91 | 0.00 | -1.40 |
| H01_G vs H01_CK | Symlandine | Alkaloids | 1.76 | 0.02 | -1.39 |
| H01_G vs H01_CK | Hypotaurine | Alkaloids | 1.70 | 0.02 | -1.35 |
| H01_G vs H01_CK | "1,7-Dimethylxanthine" | Nucleotide and its derivates | 1.81 | 0.01 | -1.15 |
| H01_G vs H01_CK | Isosakuranetin | Flavonoids | 1.88 | 0.00 | -1.10 |
| H01_G vs H01_CK | Benzocaine | Benzene and substituted derivatives | 1.89 | 0.00 | -1.09 |
| H01_G vs H01_CK | (-)-Sativan | Flavonoids | 1.63 | 0.04 | -1.06 |
| H01_G vs H01_CK | Palmitoylethanolamide | Carboximidic acids and derivatives | 1.89 | 0.00 | -1.00 |
| H01_G vs H01_CK | Rebaudioside B | Diterpenoids | 1.65 | 0.03 | -0.91 |
| H01_G vs H01_CK | Deltonin | Steroids and steroid derivatives | 1.63 | 0.04 | -0.88 |
| H01_G vs H01_CK | Anisatin | Sesquiterpenoids | 1.72 | 0.02 | -0.81 |
| H01_G vs H01_CK | Caffeine | Alkaloids | 1.79 | 0.01 | -0.75 |
| H01_G vs H01_CK | Convolvine | Alkaloids | 1.80 | 0.01 | -0.69 |
| H01_G vs H01_CK | Peonidin-3-glucoside | Flavonoids | 1.86 | 0.00 | -0.68 |
| H01_G vs H01_CK | 5-S-Methyl-5-thioadenosine | Nucleotide and its derivates | 1.70 | 0.02 | -0.67 |
| H01_G vs H01_CK | 2-Picolinic acid | Organic acids | 1.66 | 0.03 | -0.66 |
| H01_G vs H01_CK | Diosmin | Flavonoids | 1.63 | 0.04 | -0.64 |
| H01_G vs H01_CK | Eicosadienoic acid | Fatty Acyls | 1.69 | 0.03 | -0.63 |
| H01_G vs H01_CK | Jasmonic acid | Fatty Acyls | 1.59 | 0.05 | -0.59 |

| | | | | | |
|---|---|---|---|---|---|
| H01_G vs H01_CK | Fumaric acid | Organic acids and derivatives | 1.67 | 0.03 | 0.63 |
| H01_G vs H01_CK | Sesamol | Phenols | 1.68 | 0.03 | 0.63 |
| H01_G vs H01_CK | ESCULETIN | Coumarins | 1.80 | 0.01 | 0.64 |
| H01_G vs H01_CK | Desoxypeganine | Alkaloids | 1.59 | 0.05 | 0.65 |
| H01_G vs H01_CK | Chrysoeriol 7-apiosylglucoside | Flavonoids | 1.87 | 0.00 | 0.65 |
| H01_G vs H01_CK | 5-oxoproline | Amino acid and derivatives | 1.64 | 0.04 | 0.73 |
| H01_G vs H01_CK | Pimelic acid | Fatty Acyls | 1.73 | 0.02 | 0.74 |
| H01_G vs H01_CK | Sparteine | Alkaloids | 1.84 | 0.01 | 0.75 |
| H01_G vs H01_CK | Neocnidilide | Lactones | 1.68 | 0.03 | 0.79 |
| H01_G vs H01_CK | O-Succinyl-L-homoserine | Amino acid and derivatives | 1.59 | 0.05 | 0.83 |
| H01_G vs H01_CK | 5-Methyldeoxycytidine | Pyrimidine nucleosides | 1.61 | 0.04 | 0.85 |
| H01_G vs H01_CK | Bornyl acetate | Monoterpenoids | 1.82 | 0.01 | 0.86 |
| H01_G vs H01_CK | Quillaic acid | Triterpenoids | 1.62 | 0.04 | 0.88 |
| H01_G vs H01_CK | Neolitsine | Alkaloids | 1.80 | 0.01 | 0.95 |
| H01_G vs H01_CK | Precocene II | Phenols | 1.63 | 0.04 | 0.97 |
| H01_G vs H01_CK | Loganin | Terpene | 1.63 | 0.04 | 0.99 |
| H01_G vs H01_CK | Lusianthridin | Miscellaneous | 1.92 | 0.00 | 1.00 |
| H01_G vs H01_CK | Testosterone | Steroids | 1.69 | 0.03 | 1.00 |
| H01_G vs H01_CK | Moracin C | Phenols | 1.84 | 0.01 | 1.01 |
| H01_G vs H01_CK | "1H-Indole-2,3-dione" | Indoles and derivatives | 1.69 | 0.03 | 1.03 |
| H01_G vs H01_CK | Levodopa | Amino acid and derivatives | 1.66 | 0.03 | 1.05 |
| H01_G vs H01_CK | 6-Aminocaproic acid | Fatty Acyls | 1.82 | 0.01 | 1.09 |
| H01_G vs H01_CK | Undecanolactone | Miscellaneous | 1.82 | 0.01 | 1.11 |
| H01_G vs H01_CK | "5,6-DHET" | Fatty Acyls | 1.78 | 0.01 | 1.12 |
| H01_G vs H01_CK | Vincamine | Alkaloids | 1.62 | 0.04 | 1.14 |

| | | | | | |
|---|---|---|---|---|---|
| H01_G vs H01_CK | Scopolamine N-oxide hydrobromide | Alkaloids | 1.92 | 0.00 | 1.20 |
| H01_G vs H01_CK | Aloeemodin | Anthraquinones | 1.76 | 0.01 | 1.28 |
| H01_G vs H01_CK | Allocryptopine | Alkaloids | 1.73 | 0.02 | 1.31 |
| H01_G vs H01_CK | Desmethylxanthohumol | Chalcones | 1.60 | 0.05 | 1.36 |
| H01_G vs H01_CK | Icariin | flavonoids | 1.71 | 0.02 | 1.37 |
| H01_G vs H01_CK | Curzerene | Sesquiterpenoids | 1.75 | 0.02 | 1.43 |
| H01_G vs H01_CK | Pyrophosphate | Non-metal oxoanionic compounds | 1.62 | 0.04 | 1.46 |
| H01_G vs H01_CK | Mannose 6-phosphate | Organooxygen compounds | 1.70 | 0.03 | 1.50 |
| H01_G vs H01_CK | Phillyrin | Phenylpropanoids | 1.79 | 0.01 | 1.51 |
| H01_G vs H01_CK | "ent-16beta,17-dihydroxy-9(11)-kauren-19-oic acid" | Diterpenoids | 1.85 | 0.00 | 1.52 |
| H01_G vs H01_CK | 3-Ethoxy-4-hydroxybenzaldehyde | Phenols | 1.90 | 0.00 | 1.54 |
| H01_G vs H01_CK | Amabiline | Alkaloids | 1.67 | 0.03 | 1.58 |
| H01_G vs H01_CK | Tricetin | Flavonoids | 1.74 | 0.02 | 1.58 |
| H01_G vs H01_CK | Glucose 1-phosphate | Organooxygen compounds | 1.67 | 0.03 | 1.68 |
| H01_G vs H01_CK | Octyl Gallate | Phenols | 1.68 | 0.03 | 1.68 |
| H01_G vs H01_CK | Octadecanamide | Fatty Acyls | 1.75 | 0.02 | 1.69 |
| H01_G vs H01_CK | Oleamide | Fatty Acyls | 1.67 | 0.03 | 1.72 |
| H01_G vs H01_CK | Miltirone | Diterpenoids | 1.73 | 0.02 | 1.77 |
| H01_G vs H01_CK | 4-Hydroxyphenylacetylglutamic acid | Organic acids | 1.72 | 0.02 | 1.79 |
| H01_G vs H01_CK | Artemisinin | Sesquiterpenoids | 1.76 | 0.01 | 1.83 |
| H01_G vs H01_CK | 5-Aminovaleric acid | Amino acid and derivatives | 1.88 | 0.00 | 1.83 |
| H01_G vs H01_CK | Oleocanthal | Phenols | 1.94 | 0.00 | 1.93 |
| H01_G vs H01_CK | N-Acetylarylamine | Benzene and substituted derivatives | 1.60 | 0.04 | 1.99 |
| H01_G vs H01_CK | Arachidonic acid | Fatty Acyls | 1.71 | 0.02 | 2.05 |
| H01_G vs H01_CK | Palmitic acid | Lipids | 1.61 | 0.04 | 2.17 |

| | | | | | |
|---|---|---|---|---|---|
| H01_G vs H01_CK | Bergapten | Coumarins | 1.86 | 0.00 | 2.85 |
| H01_G vs H01_CK | N-((-)-jasmonoyl)-S-isoleucine | phytohormone | 1.61 | 0.04 | 3.02 |
| H01_N vs H01_CK | Stevioside | Diterpenoids | 1.90 | 0.00 | -2.49 |
| H01_N vs H01_CK | Wedelolactone | Coumarins | 1.88 | 0.01 | -2.22 |
| H01_N vs H01_CK | Suberic acid | Fatty Acyls | 1.96 | 0.00 | -1.83 |
| H01_N vs H01_CK | Mesembrine | Alkaloids | 1.78 | 0.02 | -1.78 |
| H01_N vs H01_CK | "Malvidin 3,5-diglucoside (Malvin)" | Flavonoids | 1.96 | 0.00 | -1.77 |
| H01_N vs H01_CK | 2-Picolinic acid | Organic acids | 1.73 | 0.03 | -1.45 |
| H01_N vs H01_CK | Hypotaurine | Alkaloids | 1.76 | 0.02 | -1.37 |
| H01_N vs H01_CK | Atranorin | Phenols | 1.72 | 0.03 | -1.36 |
| H01_N vs H01_CK | 4-Hydroxybenzaldehyde | Phenols | 1.77 | 0.02 | -1.34 |
| H01_N vs H01_CK | Quercetin-3-O-sophoroside | Flavonoids | 1.74 | 0.02 | -1.33 |
| H01_N vs H01_CK | Gamma-Tocotrienol | Phenols | 1.65 | 0.04 | -1.25 |
| H01_N vs H01_CK | Isoscoparin | Flavonoids | 1.93 | 0.00 | -1.21 |
| H01_N vs H01_CK | Isosakuranetin | Flavonoids | 1.99 | 0.00 | -1.17 |
| H01_N vs H01_CK | Tombozine | Alkaloids | 1.83 | 0.01 | -1.12 |
| H01_N vs H01_CK | Neolitsine | Alkaloids | 1.64 | 0.04 | -1.07 |
| H01_N vs H01_CK | Tropine acetate | Alkaloids | 1.81 | 0.01 | -1.06 |
| H01_N vs H01_CK | 7-Methylguanine | Imidazopyrimidines | 1.79 | 0.02 | -1.02 |
| H01_N vs H01_CK | Delta-Nonalactone | Miscellaneous | 1.64 | 0.05 | -0.98 |
| H01_N vs H01_CK | Morin | Flavonoids | 1.69 | 0.04 | -0.97 |
| H01_N vs H01_CK | Norbixin | Prenol lipids | 1.85 | 0.01 | -0.96 |
| H01_N vs H01_CK | Calenduloside E | Triterpenoids | 1.81 | 0.01 | -0.95 |
| H01_N vs H01_CK | Gentioflavin | Alkaloids | 1.80 | 0.01 | -0.94 |
| H01_N vs H01_CK | Riboflavine | Vitamins | 1.89 | 0.00 | -0.93 |

| | | | | | |
|---|---|---|---|---|---|
| H01_N vs H01_CK | Podophyllotoxinone | Lignans | 1.79 | 0.02 | -0.91 |
| H01_N vs H01_CK | Palmitoylethanolamide | Carboximidic acids and derivatives | 1.80 | 0.01 | -0.90 |
| H01_N vs H01_CK | "2,3,5,7-Tetrahydroxyflavone" | Flavonoids | 1.95 | 0.00 | -0.86 |
| H01_N vs H01_CK | "(R)-2-Hydroxy-2H-1,4-benzoxazin-3(4H)-one" | Benzoxazines | 1.91 | 0.00 | -0.86 |
| H01_N vs H01_CK | Convallatoxin | Steroids | 1.63 | 0.05 | -0.84 |
| H01_N vs H01_CK | "(-)-3-(3,4-Dihydroxyphenyl)-2-methylalanine" | Amino acid and its derivatives | 1.65 | 0.04 | -0.79 |
| H01_N vs H01_CK | Protocatechuic acid | Phenols | 1.66 | 0.04 | -0.73 |
| H01_N vs H01_CK | 5-S-Methyl-5-thioadenosine | Nucleotide and its derivates | 1.92 | 0.00 | -0.71 |
| H01_N vs H01_CK | Peonidin-3-glucoside | Flavonoids | 1.89 | 0.00 | -0.65 |
| H01_N vs H01_CK | Guaiacol | Phenols | 1.83 | 0.01 | -0.61 |
| H01_N vs H01_CK | N-Feruloyl putrescine | Phenolamides | 1.87 | 0.01 | 0.59 |
| H01_N vs H01_CK | Bornyl acetate | Monoterpenoids | 1.76 | 0.02 | 0.67 |
| H01_N vs H01_CK | Chrysoeriol 7-apiosylglucoside | Flavonoids | 1.78 | 0.02 | 0.67 |
| H01_N vs H01_CK | beta-Cryptoxanthin | Miscellaneous | 1.65 | 0.04 | 0.67 |
| H01_N vs H01_CK | Moracin C | Phenols | 1.82 | 0.01 | 0.70 |
| H01_N vs H01_CK | Encecalin | Phenols | 1.77 | 0.02 | 0.75 |
| H01_N vs H01_CK | "5,6-DHET" | Fatty Acyls | 1.87 | 0.01 | 0.79 |
| H01_N vs H01_CK | 5-Hydroxylysine | amino acids | 1.81 | 0.01 | 0.89 |
| H01_N vs H01_CK | Clovin | Flavonoids | 1.76 | 0.02 | 1.04 |
| H01_N vs H01_CK | Pyrophosphate | Non-metal oxoanionic compounds | 1.78 | 0.02 | 1.04 |
| H01_N vs H01_CK | "8,9-DiHETrE" | Fatty Acyls | 1.64 | 0.04 | 1.06 |
| H01_N vs H01_CK | Pterosin D | Sesquiterpenoids | 1.92 | 0.00 | 1.08 |
| H01_N vs H01_CK | Allocryptopine | Alkaloids | 1.66 | 0.04 | 1.17 |
| H01_N vs H01_CK | 4-Hydroxyphenyl-2-propionic acid | Phenylpropanoic acids | 1.72 | 0.03 | 1.21 |
| H01_N vs H01_CK | 5-Aminovaleric acid | Amino acid and derivatives | 1.89 | 0.00 | 1.26 |

| | | | | | |
|---|---|---|---|---|---|
| H01_N vs H01_CK | Desmethylxanthohumol | Chalcones | 1.78 | 0.02 | 1.33 |
| H01_N vs H01_CK | Mannose 6-phosphate | Organooxygen compounds | 1.68 | 0.04 | 1.37 |
| H01_N vs H01_CK | 6-Deoxyjacareubin | Xanthones | 1.72 | 0.03 | 1.38 |
| H01_N vs H01_CK | Octadecanamide | Fatty Acyls | 1.65 | 0.04 | 1.42 |
| H01_N vs H01_CK | Oleamide | Fatty Acyls | 1.66 | 0.04 | 1.49 |
| H01_N vs H01_CK | Amabiline | Alkaloids | 1.73 | 0.03 | 1.55 |
| H01_N vs H01_CK | Oleic acid | Fatty Acyls | 1.65 | 0.04 | 1.62 |
| H01_N vs H01_CK | Octyl Gallate | Phenols | 1.70 | 0.03 | 1.63 |
| H01_N vs H01_CK | Phytic acid | Miscellaneous | 1.65 | 0.04 | 1.65 |
| H01_N vs H01_CK | Miltirone | Diterpenoids | 1.79 | 0.02 | 1.71 |
| H01_N vs H01_CK | N-Acetylornithine | Carboxylic acids and derivatives | 1.70 | 0.03 | 1.71 |
| H01_N vs H01_CK | Glucose 1-phosphate | Organooxygen compounds | 1.65 | 0.04 | 1.71 |
| H01_N vs H01_CK | "2,6-Dimethyl-7-octene-2,3,6-triol" | Monoterpenoids | 1.76 | 0.02 | 1.72 |
| H01_N vs H01_CK | 4-Hydroxyphenylacetylglutamic acid | Organic acids | 1.75 | 0.02 | 1.76 |
| H01_N vs H01_CK | Farrerol | flavonoids | 1.83 | 0.01 | 1.80 |
| H01_N vs H01_CK | Artemisinin | Sesquiterpenoids | 1.73 | 0.03 | 1.89 |
| H01_N vs H01_CK | 4-Demethylpodophyllotoxin | Lignans | 1.90 | 0.00 | 2.07 |
| H01_N vs H01_CK | Palmitic acid | Lipids | 1.74 | 0.02 | 2.10 |
| H01_N vs H01_CK | Quercetin | flavonoids | 1.63 | 0.05 | 2.40 |
| H01_N vs H01_CK | Savinin | Lignans | 1.64 | 0.05 | 2.41 |
| H01_N vs H01_CK | 2-Deoxyribose 5-phosphate | Carbohydrates | 1.71 | 0.03 | 3.86 |
| H01_N vs H01_CK | Homoorientin | flavonoids | 1.99 | 0.00 | 4.87 |
| H20_GN vs H20_CK | Reserpine | Alkaloids | 1.84 | 0.00 | -3.18 |
| H20_GN vs H20_CK | Ginkgolic acid C15:1 | Phenols | 1.63 | 0.03 | -2.68 |
| H20_GN vs H20_CK | N-Acetyl-D-glucosamine 6-phosphate | Organooxygen compounds | 1.73 | 0.01 | -2.48 |

| | | | | | |
|---|---|---|---|---|---|
| H20_GN vs H20_CK | Bruceine D | Diterpenoids | 1.79 | 0.00 | -2.34 |
| H20_GN vs H20_CK | Saponarin | Flavonoids | 1.79 | 0.00 | -2.29 |
| H20_GN vs H20_CK | Canadine | Alkaloids | 1.82 | 0.00 | -2.25 |
| H20_GN vs H20_CK | Dyclonine | Organooxygen compounds | 1.87 | 0.00 | -2.14 |
| H20_GN vs H20_CK | pyrocatechol | flavonoids | 1.60 | 0.03 | -2.11 |
| H20_GN vs H20_CK | Cimifugin | Flavonoids | 1.60 | 0.03 | -2.10 |
| H20_GN vs H20_CK | 1-Caffeoylquinic acid | Phenylpropanoids | 1.78 | 0.01 | -2.09 |
| H20_GN vs H20_CK | 7-Hydroxyflavone | Flavonoids | 1.84 | 0.00 | -2.00 |
| H20_GN vs H20_CK | "2,3,5,7-Tetrahydroxyflavone" | Flavonoids | 1.75 | 0.01 | -1.82 |
| H20_GN vs H20_CK | Mannitol | Alcohols and polyols | 1.86 | 0.00 | -1.80 |
| H20_GN vs H20_CK | Micromelin | Coumarins | 1.58 | 0.04 | -1.79 |
| H20_GN vs H20_CK | Ganoderic acid L | Alkaloids | 1.58 | 0.04 | -1.78 |
| H20_GN vs H20_CK | Theaflavin | Flavonoids | 1.64 | 0.03 | -1.77 |
| H20_GN vs H20_CK | Quinapril | Carboxylic acids and derivatives | 1.72 | 0.01 | -1.64 |
| H20_GN vs H20_CK | Glycyrrhetinic acid | Triterpenoids | 1.72 | 0.01 | -1.61 |
| H20_GN vs H20_CK | Quercetin 3-O-neohesperidoside | Flavonoids | 1.78 | 0.01 | -1.53 |
| H20_GN vs H20_CK | Norbixin | Prenol lipids | 1.85 | 0.00 | -1.53 |
| H20_GN vs H20_CK | 6-(Furfurylamino)purine | Alkaloids | 1.70 | 0.01 | -1.52 |
| H20_GN vs H20_CK | Convallatoxin | Steroids | 1.56 | 0.04 | -1.52 |
| H20_GN vs H20_CK | Deoxyvasicinone | Alkaloids | 1.58 | 0.04 | -1.50 |
| H20_GN vs H20_CK | Lathyrol | Diterpenoids | 1.65 | 0.02 | -1.49 |
| H20_GN vs H20_CK | DL-Benzylsuccinic acid | Phenylpropanoids | 1.77 | 0.01 | -1.46 |
| H20_GN vs H20_CK | Baicalin | Flavonoids | 1.71 | 0.02 | -1.45 |
| H20_GN vs H20_CK | Cyanidin-3-O-rhamnoside chloride | Flavonoids | 1.70 | 0.01 | -1.41 |
| H20_GN vs H20_CK | Anacrotine | Alkaloids | 1.57 | 0.04 | -1.38 |

| | | | | | |
|---|---|---|---|---|---|
| H20_GN vs H20_CK | Quercetin-7-O-beta-D-glucopyranoside | Flavonoids | 1.69 | 0.01 | -1.35 |
| H20_GN vs H20_CK | "1H-Indole-2,3-dione" | Indoles and derivatives | 1.65 | 0.03 | -1.33 |
| H20_GN vs H20_CK | Capsanthin | Terpene | 1.63 | 0.03 | -1.33 |
| H20_GN vs H20_CK | Convolvine | Alkaloids | 1.77 | 0.01 | -1.30 |
| H20_GN vs H20_CK | Myristoleic acid | Lipids | 1.82 | 0.00 | -1.27 |
| H20_GN vs H20_CK | gamma-Diasarone | Lignans | 1.69 | 0.02 | -1.27 |
| H20_GN vs H20_CK | Apigenin | flavonoids | 1.58 | 0.04 | -1.24 |
| H20_GN vs H20_CK | Diosmin | Flavonoids | 1.88 | 0.00 | -1.24 |
| H20_GN vs H20_CK | Cephalotaxine | Alkaloids | 1.77 | 0.01 | -1.24 |
| H20_GN vs H20_CK | Isosakuranetin | Flavonoids | 1.84 | 0.00 | -1.22 |
| H20_GN vs H20_CK | Coniferylaldehyde | Phenylpropanoids | 1.59 | 0.04 | -1.22 |
| H20_GN vs H20_CK | Dalbergioidin | Flavonoids | 1.85 | 0.00 | -1.20 |
| H20_GN vs H20_CK | Echinocystic acid | Triterpenoids | 1.58 | 0.04 | -1.16 |
| H20_GN vs H20_CK | Glucofrangulin B | Anthraquinones | 1.86 | 0.00 | -1.15 |
| H20_GN vs H20_CK | Furfuryl acetate | Miscellaneous | 1.55 | 0.04 | -1.12 |
| H20_GN vs H20_CK | Esculin | Coumarins | 1.82 | 0.00 | -1.06 |
| H20_GN vs H20_CK | Vicenin 2 | Flavonoids | 1.73 | 0.01 | -1.04 |
| H20_GN vs H20_CK | Hordenine | Alkaloids | 1.87 | 0.00 | -1.03 |
| H20_GN vs H20_CK | D-Fructose 6-phosphate | Carbohydrates | 1.56 | 0.05 | -0.96 |
| H20_GN vs H20_CK | Isoquercitrin | flavonoids | 1.63 | 0.03 | -0.93 |
| H20_GN vs H20_CK | 3-Isomangostin hydrate | Xanthones | 1.85 | 0.00 | -0.93 |
| H20_GN vs H20_CK | Tussilagine | Alkaloids | 1.72 | 0.01 | -0.93 |
| H20_GN vs H20_CK | Ecgonine | Alkaloids | 1.68 | 0.02 | -0.92 |
| H20_GN vs H20_CK | Catalpalactone | Sesquiterpenoids | 1.79 | 0.00 | -0.91 |
| H20_GN vs H20_CK | Shikimic acid | Organic acids and derivatives | 1.78 | 0.01 | -0.89 |

| | | | | | |
|---|---|---|---|---|---|
| H20_GN vs H20_CK | Cynaroside | flavonoids | 1.87 | 0.00 | -0.88 |
| H20_GN vs H20_CK | Pyrocurzerenone | Sesquiterpenoids | 1.56 | 0.04 | -0.86 |
| H20_GN vs H20_CK | Peonidin-3-glucoside | Flavonoids | 1.87 | 0.00 | -0.85 |
| H20_GN vs H20_CK | Kaempferol-3-O-rutinoside | flavonoids | 1.80 | 0.00 | -0.84 |
| H20_GN vs H20_CK | Morin | Flavonoids | 1.67 | 0.02 | -0.83 |
| H20_GN vs H20_CK | Homoeriodictyol | Flavonoids | 1.79 | 0.00 | -0.83 |
| H20_GN vs H20_CK | "2,6-Dimethyl-7-octene-2,3,6-triol" | Monoterpenoids | 1.62 | 0.03 | -0.82 |
| H20_GN vs H20_CK | Ferulic acid | Phenylpropanoids | 1.75 | 0.01 | -0.82 |
| H20_GN vs H20_CK | Curzerene | Sesquiterpenoids | 1.60 | 0.03 | -0.82 |
| H20_GN vs H20_CK | N5-(L-1-Carboxyethyl)-L-ornithine | Organic acids | 1.60 | 0.03 | -0.80 |
| H20_GN vs H20_CK | Glycyrrhetic acid 3-O-mono-beta-D-glucuronide | Triterpenoids | 1.78 | 0.01 | -0.77 |
| H20_GN vs H20_CK | Rutin | flavonoids | 1.80 | 0.00 | -0.76 |
| H20_GN vs H20_CK | Glutaric acid | Organic acids and derivatives | 1.79 | 0.00 | -0.74 |
| H20_GN vs H20_CK | 3-Hydroxyphenylacetic acid | Phenols | 1.83 | 0.00 | -0.72 |
| H20_GN vs H20_CK | D-(-)-Quinic acid | Quinolines and derivatives | 1.86 | 0.00 | -0.70 |
| H20_GN vs H20_CK | 8-Hydroxybergapten | Coumarins | 1.69 | 0.02 | -0.69 |
| H20_GN vs H20_CK | 4-Hydroxybenzoic acid | Phenols | 1.76 | 0.01 | -0.69 |
| H20_GN vs H20_CK | Protocatechuic acid | Phenols | 1.75 | 0.01 | -0.69 |
| H20_GN vs H20_CK | Kaurenoic acid | Diterpenoids | 1.70 | 0.02 | -0.67 |
| H20_GN vs H20_CK | Sakuranetin | Flavonoids | 1.80 | 0.00 | -0.66 |
| H20_GN vs H20_CK | vitamin K2 | Vitamins | 1.75 | 0.01 | -0.64 |
| H20_GN vs H20_CK | 5-S-Methyl-5-thioadenosine | Nucleotide and its derivates | 1.70 | 0.01 | -0.63 |
| H20_GN vs H20_CK | (+)-Afzelechin | Flavonoids | 1.75 | 0.01 | -0.63 |
| H20_GN vs H20_CK | Sipeimine | Alkaloids | 1.54 | 0.05 | -0.61 |
| H20_GN vs H20_CK | (-)-Isocorypalmine | Alkaloids | 1.64 | 0.03 | 0.59 |

| | | | | | |
|---|---|---|---|---|---|
| H20_GN vs H20_CK | "3,4-Dihydroxybenzaldehyde" | Phenols | 1.76 | 0.01 | 0.67 |
| H20_GN vs H20_CK | Suberic acid | Fatty Acyls | 1.69 | 0.02 | 0.72 |
| H20_GN vs H20_CK | Deoxyguanosine | Nucleotide and its derivates | 1.81 | 0.00 | 0.89 |
| H20_GN vs H20_CK | Calenduloside E | Triterpenoids | 1.60 | 0.03 | 0.91 |
| H20_GN vs H20_CK | "4,7-Dihydroxyflavone" | Flavonoids | 1.81 | 0.00 | 0.92 |
| H20_GN vs H20_CK | Cassiaside B | Phenols | 1.69 | 0.02 | 1.08 |
| H20_GN vs H20_CK | Pyridoxine | Vitamins | 1.84 | 0.00 | 1.37 |
| H20_GN vs H20_CK | Genipin-1-O-gentiobioside | Iridoids | 1.69 | 0.02 | 2.15 |
| H20_GN vs H20_CK | AICAR | Imidazole ribonucleosides and ribonucleotides | 1.63 | 0.03 | 2.23 |
| H20_GN vs H20_CK | Carbendazim | Benzimidazoles | 1.78 | 0.00 | 3.70 |
| H20_GN vs H20_G | Psychosine | Sphingolipids | 2.06 | 0.00 | -3.55 |
| H20_GN vs H20_G | Icariin | flavonoids | 1.96 | 0.01 | -2.49 |
| H20_GN vs H20_G | N-Acetyl-D-glucosamine 6-phosphate | Organooxygen compounds | 2.04 | 0.00 | -2.22 |
| H20_GN vs H20_G | Fumitremorgin B | Alkaloids | 1.70 | 0.05 | -2.20 |
| H20_GN vs H20_G | Malvidin-3-O-galactoside | Flavonoids | 1.92 | 0.01 | -2.06 |
| H20_GN vs H20_G | Reserpine | Alkaloids | 1.84 | 0.02 | -1.89 |
| H20_GN vs H20_G | Picrotoxinin | Sesquiterpenoids | 1.78 | 0.03 | -1.87 |
| H20_GN vs H20_G | Saponarin | Flavonoids | 1.85 | 0.02 | -1.71 |
| H20_GN vs H20_G | N6-isopentenyladenosine | phytohormone | 1.74 | 0.04 | -1.58 |
| H20_GN vs H20_G | Inosine 5-monophosphate | Nucleotide and its derivates | 1.85 | 0.02 | -1.52 |
| H20_GN vs H20_G | Micromelin | Coumarins | 1.84 | 0.02 | -1.50 |
| H20_GN vs H20_G | Convallatoxin | Steroids | 1.96 | 0.01 | -1.32 |
| H20_GN vs H20_G | N-Feruloyl putrescine | Phenolamides | 2.05 | 0.00 | -1.29 |
| H20_GN vs H20_G | Coniferylaldehyde | Phenylpropanoids | 1.79 | 0.03 | -1.17 |

| | | | | | |
|---|---|---|---|---|---|
| H20_GN vs H20_G | Testosterone | Steroids | 1.92 | 0.01 | -1.13 |
| H20_GN vs H20_G | Chrysoeriol 7-apiosylglucoside | Flavonoids | 1.97 | 0.00 | -1.13 |
| H20_GN vs H20_G | Zeorin | Triterpenoids | 1.80 | 0.03 | -1.11 |
| H20_GN vs H20_G | Baicalin | Flavonoids | 1.74 | 0.04 | -1.01 |
| H20_GN vs H20_G | Swertiamarin | Iridoids | 1.74 | 0.04 | -0.91 |
| H20_GN vs H20_G | Deoxyguanosine | Nucleotide and its derivates | 1.73 | 0.04 | -0.89 |
| H20_GN vs H20_G | Dyclonine | Organooxygen compounds | 1.92 | 0.01 | -0.88 |
| H20_GN vs H20_G | Cyanidin-3-O-rhamnoside chloride | Flavonoids | 1.76 | 0.03 | -0.83 |
| H20_GN vs H20_G | Samidin | Coumarins | 1.77 | 0.03 | -0.82 |
| H20_GN vs H20_G | Rhoifolin | flavonoids | 1.95 | 0.01 | -0.82 |
| H20_GN vs H20_G | Absinthiin | Triterpenoids | 1.78 | 0.03 | -0.79 |
| H20_GN vs H20_G | Quercetin-7-O-beta-D-glucopyranoside | Flavonoids | 1.83 | 0.02 | -0.73 |
| H20_GN vs H20_G | Vicenin 2 | Flavonoids | 1.79 | 0.03 | -0.73 |
| H20_GN vs H20_G | Deoxyvasicinone | Alkaloids | 1.75 | 0.04 | -0.70 |
| H20_GN vs H20_G | 1H-Indole-3-carboxylic acid | Alkaloids | 1.98 | 0.00 | -0.69 |
| H20_GN vs H20_G | Pimelic acid | Fatty Acyls | 1.77 | 0.03 | -0.68 |
| H20_GN vs H20_G | Isovitexin | Flavonoids | 1.99 | 0.00 | -0.67 |
| H20_GN vs H20_G | Esculin | Coumarins | 1.97 | 0.01 | -0.63 |
| H20_GN vs H20_G | Fumaric acid | Organic acids and derivatives | 1.96 | 0.01 | -0.62 |
| H20_GN vs H20_G | Cassythicine | Alkaloids | 1.83 | 0.02 | 0.65 |
| H20_GN vs H20_G | Isoquercitrin | flavonoids | 1.73 | 0.04 | 0.65 |
| H20_GN vs H20_G | Leukotriene A4 | Fatty Acyls | 2.05 | 0.00 | 0.66 |
| H20_GN vs H20_G | Kaempferol-3-O-rutinoside | flavonoids | 2.01 | 0.00 | 0.69 |
| H20_GN vs H20_G | Benzyl cinnamate | Phenylpropanoids | 1.84 | 0.02 | 0.69 |
| H20_GN vs H20_G | Narcissoside | flavonoids | 2.01 | 0.00 | 0.69 |

| | | | | | |
|---|---|---|---|---|---|
| H20_GN vs H20_G | Acetyl tryptophan | Amino acid and derivatives | 1.71 | 0.05 | 0.81 |
| H20_GN vs H20_G | Isosakuranetin | Flavonoids | 2.02 | 0.00 | 0.86 |
| H20_GN vs H20_G | Isoscoparin | Flavonoids | 2.00 | 0.00 | 0.91 |
| H20_GN vs H20_G | Phosphonoacetate | Organic acids | 1.72 | 0.04 | 0.94 |
| H20_GN vs H20_G | 1-Naphthol | Naphthalenes | 1.78 | 0.03 | 0.95 |
| H20_GN vs H20_G | 7-Ethoxycoumarin | Coumarins | 1.76 | 0.03 | 0.99 |
| H20_GN vs H20_G | Scopolamine N-oxide hydrobromide | Alkaloids | 1.77 | 0.04 | 1.05 |
| H20_GN vs H20_G | Lactaroviolin | Prenol lipids | 1.82 | 0.02 | 1.09 |
| H20_GN vs H20_G | Oxoadipic acid | Organic acids | 1.87 | 0.02 | 1.22 |
| H20_GN vs H20_G | 2-Picolinic acid | Organic acids | 1.86 | 0.02 | 1.30 |
| H20_GN vs H20_G | Euxanthone | Xanthones | 1.87 | 0.02 | 1.40 |
| H20_GN vs H20_G | Naringenin | flavonoids | 2.03 | 0.00 | 1.41 |
| H20_GN vs H20_G | 3-O-Acetylpinobanksin | Flavonoids | 1.77 | 0.03 | 1.68 |
| H20_GN vs H20_G | Ponasterone A | Steroids | 1.97 | 0.01 | 1.86 |
| H20_GN vs H20_G | Homoeriodictyol | Flavonoids | 1.92 | 0.01 | 2.21 |
| H20_GN vs H20_G | Suberic acid | Fatty Acyls | 2.07 | 0.00 | 2.95 |
| H20_GN vs H20_G | Genipin-1-O-gentiobioside | Iridoids | 1.93 | 0.01 | 3.60 |
| H20_GN vs H20_N | alpha-Tocopherol | Phenols | 1.84 | 0.01 | -2.82 |
| H20_GN vs H20_N | Picrotoxinin | Sesquiterpenoids | 1.69 | 0.03 | -2.73 |
| H20_GN vs H20_N | Fumitremorgin B | Alkaloids | 1.77 | 0.02 | -2.36 |
| H20_GN vs H20_N | Reserpine | Alkaloids | 1.86 | 0.01 | -2.26 |
| H20_GN vs H20_N | Forskolin | Diterpenoids | 1.68 | 0.04 | -2.20 |
| H20_GN vs H20_N | Petasitenine | Alkaloids | 1.70 | 0.03 | -2.05 |
| H20_GN vs H20_N | Saponarin | Flavonoids | 1.78 | 0.02 | -1.81 |
| H20_GN vs H20_N | Gamma-Tocotrienol | Phenols | 1.66 | 0.04 | -1.79 |

| | | | | | |
|---|---|---|---|---|---|
| H20_GN vs H20_N | N-Acetyl-L-glutamate 5-semialdehyde | Carboxylic acids and derivatives | 1.93 | 0.00 | -1.75 |
| H20_GN vs H20_N | Aloeemodin | Anthraquinones | 1.79 | 0.02 | -1.67 |
| H20_GN vs H20_N | (+/-)-Jasmonic acid | phytohormone | 1.81 | 0.01 | -1.58 |
| H20_GN vs H20_N | Tetrahymanol | Triterpenoids | 1.70 | 0.03 | -1.51 |
| H20_GN vs H20_N | Folinic acid | Pteridines and derivatives | 1.80 | 0.01 | -1.47 |
| H20_GN vs H20_N | Niloticin | Triterpenoids | 1.67 | 0.04 | -1.45 |
| H20_GN vs H20_N | Cimifugin | Flavonoids | 1.64 | 0.05 | -1.43 |
| H20_GN vs H20_N | 3-Methylxanthine | Nucleotide and its derivates | 1.93 | 0.00 | -1.30 |
| H20_GN vs H20_N | Sesamol | Phenols | 1.64 | 0.05 | -1.25 |
| H20_GN vs H20_N | Testosterone | Steroids | 1.85 | 0.01 | -1.14 |
| H20_GN vs H20_N | Dodecanedioic aicd | Fatty Acyls | 1.64 | 0.05 | -1.12 |
| H20_GN vs H20_N | L-Malic acid | Hydroxy acids and derivatives | 1.72 | 0.03 | -1.11 |
| H20_GN vs H20_N | Lathyrol | Diterpenoids | 1.67 | 0.04 | -1.04 |
| H20_GN vs H20_N | beta-Asarone | Phenylpropanoids | 1.65 | 0.04 | -1.02 |
| H20_GN vs H20_N | Dihydrokavain | Phenols | 1.68 | 0.04 | -1.01 |
| H20_GN vs H20_N | Parthenolide | Sesquiterpenoids | 1.88 | 0.01 | -0.99 |
| H20_GN vs H20_N | Lactupicrin | Sesquiterpenoids | 1.62 | 0.05 | -0.98 |
| H20_GN vs H20_N | 3-Isomangostin hydrate | Xanthones | 1.97 | 0.00 | -0.96 |
| H20_GN vs H20_N | Chrysoeriol 7-apiosylglucoside | Flavonoids | 1.88 | 0.01 | -0.95 |
| H20_GN vs H20_N | N-Feruloyl putrescine | Phenolamides | 1.93 | 0.00 | -0.93 |
| H20_GN vs H20_N | Esculin | Coumarins | 1.85 | 0.01 | -0.90 |
| H20_GN vs H20_N | Dyclonine | Organooxygen compounds | 1.64 | 0.05 | -0.86 |
| H20_GN vs H20_N | Samidin | Coumarins | 1.70 | 0.03 | -0.80 |
| H20_GN vs H20_N | Hordenine | Alkaloids | 1.92 | 0.00 | -0.78 |
| H20_GN vs H20_N | 5-Carboxyvanillic acid | Organic acids | 1.70 | 0.03 | -0.76 |

| | | | | | |
|---|---|---|---|---|---|
| H20_GN vs H20_N | 5-oxoproline | Amino acid and derivatives | 1.78 | 0.02 | -0.75 |
| H20_GN vs H20_N | Rhoifolin | flavonoids | 1.75 | 0.02 | -0.75 |
| H20_GN vs H20_N | Protocatechuic acid | Phenols | 1.62 | 0.05 | -0.74 |
| H20_GN vs H20_N | Decursinol | Coumarins | 1.86 | 0.01 | -0.71 |
| H20_GN vs H20_N | Cynaroside | flavonoids | 1.87 | 0.01 | -0.71 |
| H20_GN vs H20_N | 4-Hydroxyphenylacetic acid | Phenols | 1.73 | 0.03 | -0.70 |
| H20_GN vs H20_N | Isovitexin | Flavonoids | 1.80 | 0.02 | -0.61 |
| H20_GN vs H20_N | Diphenylamine | Benzene and substituted derivatives | 1.67 | 0.04 | 0.63 |
| H20_GN vs H20_N | L-Ornithine | Amino acid and derivatives | 1.82 | 0.01 | 0.63 |
| H20_GN vs H20_N | Neoglycyrol | Coumarins | 1.92 | 0.00 | 0.72 |
| H20_GN vs H20_N | Isosakuranetin | Flavonoids | 1.93 | 0.00 | 0.73 |
| H20_GN vs H20_N | Harmaline | Alkaloids | 1.89 | 0.00 | 0.73 |
| H20_GN vs H20_N | Cosmosiin | Flavonoids | 1.97 | 0.00 | 0.77 |
| H20_GN vs H20_N | Acetyl tryptophan | Amino acid and derivatives | 1.67 | 0.04 | 0.84 |
| H20_GN vs H20_N | Narcissoside | flavonoids | 1.91 | 0.00 | 0.89 |
| H20_GN vs H20_N | Isoscoparin | Flavonoids | 1.94 | 0.00 | 0.90 |
| H20_GN vs H20_N | Kaempferol-3-O-rutinoside | flavonoids | 1.97 | 0.00 | 0.91 |
| H20_GN vs H20_N | vitamin K2 | Vitamins | 1.85 | 0.01 | 1.24 |
| H20_GN vs H20_N | Oxoadipic acid | Organic acids | 1.72 | 0.03 | 1.26 |
| H20_GN vs H20_N | Nandrolone | Steroids and steroid derivatives | 1.75 | 0.02 | 1.28 |
| H20_GN vs H20_N | Scopolamine N-oxide hydrobromide | Alkaloids | 1.86 | 0.01 | 1.31 |
| H20_GN vs H20_N | Jervine | Alkaloids | 1.91 | 0.00 | 1.35 |
| H20_GN vs H20_N | Homoeriodictyol | Flavonoids | 1.99 | 0.00 | 1.43 |
| H20_GN vs H20_N | Piperlonguminine | Alkaloids | 1.64 | 0.05 | 1.44 |
| H20_GN vs H20_N | Pyridoxine | Vitamins | 1.77 | 0.02 | 1.45 |

| | | | | | |
|---|---|---|---|---|---|
| H20_GN vs H20_N | 2-Picolinic acid | Organic acids | 1.89 | 0.00 | 1.55 |
| H20_GN vs H20_N | Suberic acid | Fatty Acyls | 1.96 | 0.00 | 1.90 |
| H20_GN vs H20_N | Naringenin | flavonoids | 1.94 | 0.00 | 2.02 |
| H20_GN vs H20_N | Genipin-1-O-gentiobioside | Iridoids | 1.76 | 0.02 | 2.18 |
| H20_GN vs H20_N | N1-Methyl-2-pyridone-5-carboxamide | Pyridines and derivatives | 1.99 | 0.00 | 2.95 |
| H20_GN vs H20_N | Carbendazim | Benzimidazoles | 1.88 | 0.01 | 3.38 |
| H20_GN vs H20_N | N1-Methyl-4-pyridone-3-carboxamide | Pyridines and derivatives | 2.00 | 0.00 | 3.89 |
| H20_GN vs H20_N | Inosine | Nucleotide and its derivates | 2.00 | 0.00 | 8.53 |
| H20_G vs H20_CK | Ganoderic acid F | Triterpenoids | 1.55 | 0.03 | -3.13 |
| H20_G vs H20_CK | Homoeriodictyol | Flavonoids | 1.75 | 0.00 | -3.04 |
| H20_G vs H20_CK | Ponasterone A | Steroids | 1.64 | 0.01 | -2.57 |
| H20_G vs H20_CK | "2,3,5,7-Tetrahydroxyflavone" | Flavonoids | 1.77 | 0.00 | -2.43 |
| H20_G vs H20_CK | Medicagenic acid | Triterpenoids | 1.49 | 0.05 | -2.41 |
| H20_G vs H20_CK | Bruceine D | Diterpenoids | 1.75 | 0.00 | -2.30 |
| H20_G vs H20_CK | Suberic acid | Fatty Acyls | 1.73 | 0.00 | -2.23 |
| H20_G vs H20_CK | Hesperetin | flavonoids | 1.55 | 0.03 | -2.14 |
| H20_G vs H20_CK | Ginkgolic acid C15:1 | Phenols | 1.54 | 0.03 | -2.11 |
| H20_G vs H20_CK | Isosakuranetin | Flavonoids | 1.78 | 0.00 | -2.08 |
| H20_G vs H20_CK | Uplandicine | Alkaloids | 1.54 | 0.03 | -1.99 |
| H20_G vs H20_CK | Norbixin | Prenol lipids | 1.74 | 0.00 | -1.97 |
| H20_G vs H20_CK | gamma-Diasarone | Lignans | 1.69 | 0.01 | -1.96 |
| H20_G vs H20_CK | (1R)-(-)-Menthyl acetate | Monoterpenoids | 1.79 | 0.00 | -1.92 |
| H20_G vs H20_CK | Naringenin | flavonoids | 1.52 | 0.04 | -1.80 |
| H20_G vs H20_CK | Trehalose 6-phosphate | Carbohydrates | 1.64 | 0.01 | -1.80 |
| H20_G vs H20_CK | Enoxacin | Diazanaphthalenes | 1.52 | 0.04 | -1.80 |

| | | | | | |
|---|---|---|---|---|---|
| H20_G vs H20_CK | Flavone | Flavonoids | 1.48 | 0.05 | -1.78 |
| H20_G vs H20_CK | Eriodictyol | flavonoids | 1.67 | 0.01 | -1.66 |
| H20_G vs H20_CK | 1-Caffeoylquinic acid | Phenylpropanoids | 1.68 | 0.01 | -1.60 |
| H20_G vs H20_CK | Isoquercitrin | flavonoids | 1.65 | 0.01 | -1.59 |
| H20_G vs H20_CK | vitamin K2 | Vitamins | 1.69 | 0.01 | -1.58 |
| H20_G vs H20_CK | Dalbergioidin | Flavonoids | 1.78 | 0.00 | -1.54 |
| H20_G vs H20_CK | Kaempferol-3-O-rutinoside | flavonoids | 1.77 | 0.00 | -1.53 |
| H20_G vs H20_CK | Nandrolone | Steroids and steroid derivatives | 1.74 | 0.00 | -1.51 |
| H20_G vs H20_CK | 7-Hydroxyflavone | Flavonoids | 1.75 | 0.00 | -1.49 |
| H20_G vs H20_CK | Echinocystic acid | Triterpenoids | 1.66 | 0.01 | -1.49 |
| H20_G vs H20_CK | Piperlonguminine | Alkaloids | 1.76 | 0.00 | -1.45 |
| H20_G vs H20_CK | 6-(Furfurylamino)purine | Alkaloids | 1.72 | 0.00 | -1.45 |
| H20_G vs H20_CK | Genipin-1-O-gentiobioside | Iridoids | 1.62 | 0.02 | -1.45 |
| H20_G vs H20_CK | Anacrotine | Alkaloids | 1.50 | 0.04 | -1.44 |
| H20_G vs H20_CK | Beta-Elemonic acid | Triterpenoids | 1.55 | 0.03 | -1.36 |
| H20_G vs H20_CK | Lathyrol | Diterpenoids | 1.47 | 0.05 | -1.32 |
| H20_G vs H20_CK | Ganoderal A | Triterpenoids | 1.51 | 0.04 | -1.32 |
| H20_G vs H20_CK | Shikonin | Quinones | 1.54 | 0.03 | -1.32 |
| H20_G vs H20_CK | Reserpine | Alkaloids | 1.66 | 0.01 | -1.30 |
| H20_G vs H20_CK | Euxanthone | Xanthones | 1.61 | 0.02 | -1.27 |
| H20_G vs H20_CK | Dyclonine | Organooxygen compounds | 1.78 | 0.00 | -1.26 |
| H20_G vs H20_CK | Alizarin 2-methyl ether | Anthraquinones | 1.48 | 0.05 | -1.24 |
| H20_G vs H20_CK | Diosmin | Flavonoids | 1.76 | 0.00 | -1.14 |
| H20_G vs H20_CK | Morin | Flavonoids | 1.51 | 0.04 | -1.14 |
| H20_G vs H20_CK | Retinoic acid | Diterpenoids | 1.51 | 0.04 | -1.14 |

| | | | | | |
|---|---|---|---|---|---|
| H20_G vs H20_CK | Myristoleic acid | Lipids | 1.67 | 0.01 | -1.11 |
| H20_G vs H20_CK | Catalpalactone | Sesquiterpenoids | 1.70 | 0.01 | -1.11 |
| H20_G vs H20_CK | Physalin L | Steroids and steroid derivatives | 1.48 | 0.05 | -1.11 |
| H20_G vs H20_CK | Isoscoparin | Flavonoids | 1.64 | 0.01 | -1.10 |
| H20_G vs H20_CK | Sakuranetin | Flavonoids | 1.79 | 0.00 | -1.10 |
| H20_G vs H20_CK | 1-Naphthol | Naphthalenes | 1.58 | 0.03 | -1.07 |
| H20_G vs H20_CK | Quercetin 3-O-neohesperidoside | Flavonoids | 1.70 | 0.01 | -1.07 |
| H20_G vs H20_CK | Rutin | flavonoids | 1.75 | 0.00 | -1.07 |
| H20_G vs H20_CK | Narcissoside | flavonoids | 1.79 | 0.00 | -1.07 |
| H20_G vs H20_CK | Pyrocurzerenone | Sesquiterpenoids | 1.51 | 0.04 | -1.04 |
| H20_G vs H20_CK | Allicin | Miscellaneous | 1.51 | 0.04 | -1.01 |
| H20_G vs H20_CK | 6-Hydroxymelatonin | Tryptamine derivatives | 1.58 | 0.02 | -1.00 |
| H20_G vs H20_CK | Oleuroside | Iridoids | 1.53 | 0.03 | -1.00 |
| H20_G vs H20_CK | Aloperine | Alkaloids | 1.59 | 0.02 | -0.98 |
| H20_G vs H20_CK | Myricetin | flavonoids | 1.50 | 0.04 | -0.97 |
| H20_G vs H20_CK | Glycyrrhetic acid 3-O-mono-beta-D-glucuronide | Triterpenoids | 1.68 | 0.01 | -0.96 |
| H20_G vs H20_CK | "(R)-2-Hydroxy-2H-1,4-benzoxazin-3(4H)-one" | Benzoxazines | 1.67 | 0.01 | -0.96 |
| H20_G vs H20_CK | Levodopa | Amino acid and derivatives | 1.72 | 0.00 | -0.94 |
| H20_G vs H20_CK | Glucofrangulin B | Anthraquinones | 1.76 | 0.00 | -0.92 |
| H20_G vs H20_CK | Curzerene | Sesquiterpenoids | 1.64 | 0.01 | -0.88 |
| H20_G vs H20_CK | Sipeimine | Alkaloids | 1.54 | 0.03 | -0.87 |
| H20_G vs H20_CK | 3-Isomangostin hydrate | Xanthones | 1.77 | 0.00 | -0.86 |
| H20_G vs H20_CK | 4-Hydroxybenzoic acid | Phenols | 1.72 | 0.00 | -0.86 |
| H20_G vs H20_CK | 3-Hydroxyphenylacetic acid | Phenols | 1.80 | 0.00 | -0.82 |
| H20_G vs H20_CK | 2-Benzal-4-hydroxyacetophenone | Flavonoids | 1.55 | 0.03 | -0.82 |

| | | | | | |
|---|---|---|---|---|---|
| H20_G vs H20_CK | 3-Hydroxy-4-methoxycinnamic acid | Phenylpropanoids | 1.61 | 0.02 | -0.81 |
| H20_G vs H20_CK | Malonic acid | Carboxylic acids and derivatives | 1.53 | 0.04 | -0.81 |
| H20_G vs H20_CK | Calycosin | Flavonoids | 1.49 | 0.05 | -0.81 |
| H20_G vs H20_CK | Cephalotaxine | Alkaloids | 1.72 | 0.00 | -0.75 |
| H20_G vs H20_CK | Ferulic acid | Phenylpropanoids | 1.69 | 0.01 | -0.74 |
| H20_G vs H20_CK | Glutaric acid | Organic acids and derivatives | 1.75 | 0.00 | -0.72 |
| H20_G vs H20_CK | Hordenine | Alkaloids | 1.77 | 0.00 | -0.70 |
| H20_G vs H20_CK | (+)-Afzelechin | Flavonoids | 1.58 | 0.02 | -0.69 |
| H20_G vs H20_CK | Isatidine | Alkaloids | 1.49 | 0.05 | -0.69 |
| H20_G vs H20_CK | 5-Aminovaleric acid | Amino acid and derivatives | 1.79 | 0.00 | -0.68 |
| H20_G vs H20_CK | Baicalein | Flavonoids | 1.56 | 0.03 | -0.64 |
| H20_G vs H20_CK | Kaurenoic acid | Diterpenoids | 1.56 | 0.03 | -0.62 |
| H20_G vs H20_CK | D-(-)-Quinic acid | Quinolines and derivatives | 1.71 | 0.01 | -0.61 |
| H20_G vs H20_CK | 1-O-Caffeoylglucose | Carbohydrates | 1.52 | 0.04 | 0.62 |
| H20_G vs H20_CK | 7-Methylxanthine | Nucleotide and its derivates | 1.56 | 0.03 | 0.63 |
| H20_G vs H20_CK | Fumaric acid | Organic acids and derivatives | 1.74 | 0.00 | 0.68 |
| H20_G vs H20_CK | Chamazulene | lipids | 1.48 | 0.05 | 0.81 |
| H20_G vs H20_CK | Pimelic acid | Fatty Acyls | 1.63 | 0.01 | 0.82 |
| H20_G vs H20_CK | N-Feruloyl putrescine | Phenolamides | 1.68 | 0.01 | 1.02 |
| H20_G vs H20_CK | Absinthiin | Triterpenoids | 1.66 | 0.01 | 1.13 |
| H20_G vs H20_CK | Cordycepin | Nucleotide and its derivates | 1.54 | 0.03 | 1.16 |
| H20_G vs H20_CK | L-Ornithine | Amino acid and derivatives | 1.57 | 0.03 | 1.17 |
| H20_G vs H20_CK | Inosine 5-monophosphate | Nucleotide and its derivates | 1.63 | 0.01 | 1.29 |
| H20_G vs H20_CK | 7-Ethyl-10-Hydroxycamptothecin | Alkaloids | 1.51 | 0.04 | 1.41 |
| H20_G vs H20_CK | CYS-GLY | Amino acid and derivatives | 1.69 | 0.01 | 1.42 |

| | | | | | |
|---|---|---|---|---|---|
| H20_G vs H20_CK | "9,10-DHOME" | Fatty Acyls | 1.63 | 0.02 | 1.56 |
| H20_G vs H20_CK | Panaxynol | Miscellaneous | 1.64 | 0.01 | 1.70 |
| H20_G vs H20_CK | Bergamotine | Coumarins | 1.71 | 0.00 | 1.71 |
| H20_G vs H20_CK | Deoxyguanosine | Nucleotide and its derivates | 1.65 | 0.01 | 1.77 |
| H20_G vs H20_CK | Mannose 6-phosphate | Organooxygen compounds | 1.56 | 0.03 | 1.79 |
| H20_G vs H20_CK | Amabiline | Alkaloids | 1.59 | 0.02 | 1.81 |
| H20_G vs H20_CK | Desmethylxanthohumol | Chalcones | 1.56 | 0.03 | 1.81 |
| H20_G vs H20_CK | (+)-Corynoline | Alkaloids | 1.60 | 0.02 | 1.86 |
| H20_G vs H20_CK | Octadecanamide | Fatty Acyls | 1.78 | 0.00 | 1.95 |
| H20_G vs H20_CK | Oleic acid | Fatty Acyls | 1.72 | 0.00 | 2.03 |
| H20_G vs H20_CK | 2-(Methylamino)benzoic acid | Benzene and substituted derivatives | 1.59 | 0.02 | 2.04 |
| H20_G vs H20_CK | Oleamide | Fatty Acyls | 1.57 | 0.03 | 2.08 |
| H20_G vs H20_CK | 4-Hydroxyphenylacetylglutamic acid | Organic acids | 1.72 | 0.00 | 2.14 |
| H20_G vs H20_CK | Artemisinin | Sesquiterpenoids | 1.69 | 0.01 | 2.16 |
| H20_G vs H20_CK | Miltirone | Diterpenoids | 1.65 | 0.01 | 2.16 |
| H20_G vs H20_CK | Octyl Gallate | Phenols | 1.62 | 0.02 | 2.17 |
| H20_G vs H20_CK | Palmitic acid | Lipids | 1.71 | 0.00 | 2.44 |
| H20_G vs H20_CK | 7-(4-Hydroxyphenyl)-1-phenyl-4-hepten-3-one | Phenols | 1.68 | 0.01 | 2.64 |
| H20_N vs H20_CK | Ganoderic acid F | Triterpenoids | 1.73 | 0.02 | -5.19 |
| H20_N vs H20_CK | Oxychelerythrine | Alkaloids | 1.72 | 0.02 | -2.69 |
| H20_N vs H20_CK | Naringenin | flavonoids | 1.69 | 0.03 | -2.41 |
| H20_N vs H20_CK | Hesperetin | flavonoids | 1.67 | 0.03 | -2.37 |
| H20_N vs H20_CK | "2,3,5,7-Tetrahydroxyflavone" | Flavonoids | 1.91 | 0.00 | -2.32 |
| H20_N vs H20_CK | Quinapril | Carboxylic acids and derivatives | 1.84 | 0.00 | -2.29 |
| H20_N vs H20_CK | Homoeriodictyol | Flavonoids | 1.92 | 0.00 | -2.26 |

| | | | | | |
|---|---|---|---|---|---|
| H20_N vs H20_CK | Enoxacin | Diazanaphthalenes | 1.65 | 0.03 | -2.22 |
| H20_N vs H20_CK | Canadine | Alkaloids | 1.88 | 0.00 | -2.05 |
| H20_N vs H20_CK | Theaflavin | Flavonoids | 1.70 | 0.02 | -2.05 |
| H20_N vs H20_CK | Diosmin | Flavonoids | 1.87 | 0.00 | -2.03 |
| H20_N vs H20_CK | Isosakuranetin | Flavonoids | 1.92 | 0.00 | -1.95 |
| H20_N vs H20_CK | Norbixin | Prenol lipids | 1.84 | 0.00 | -1.87 |
| H20_N vs H20_CK | vitamin K2 | Vitamins | 1.89 | 0.00 | -1.87 |
| H20_N vs H20_CK | Nandrolone | Steroids and steroid derivatives | 1.90 | 0.00 | -1.83 |
| H20_N vs H20_CK | Piperlonguminine | Alkaloids | 1.93 | 0.00 | -1.79 |
| H20_N vs H20_CK | Kaempferol-3-O-rutinoside | flavonoids | 1.91 | 0.00 | -1.75 |
| H20_N vs H20_CK | 7-Hydroxyflavone | Flavonoids | 1.92 | 0.00 | -1.72 |
| H20_N vs H20_CK | beta-Cryptoxanthin | Miscellaneous | 1.68 | 0.03 | -1.71 |
| H20_N vs H20_CK | (1R)-(-)-Menthyl acetate | Monoterpenoids | 1.79 | 0.01 | -1.59 |
| H20_N vs H20_CK | 6-(Furfurylamino)purine | Alkaloids | 1.90 | 0.00 | -1.52 |
| H20_N vs H20_CK | Bruceine D | Diterpenoids | 1.62 | 0.04 | -1.43 |
| H20_N vs H20_CK | Trehalose 6-phosphate | Carbohydrates | 1.69 | 0.03 | -1.41 |
| H20_N vs H20_CK | Acacetin | Flavonoids | 1.70 | 0.02 | -1.39 |
| H20_N vs H20_CK | Morin | Flavonoids | 1.77 | 0.01 | -1.32 |
| H20_N vs H20_CK | Dyclonine | Organooxygen compounds | 1.90 | 0.00 | -1.28 |
| H20_N vs H20_CK | 1-Caffeoylquinic acid | Phenylpropanoids | 1.60 | 0.05 | -1.26 |
| H20_N vs H20_CK | Narcissoside | flavonoids | 1.92 | 0.00 | -1.26 |
| H20_N vs H20_CK | Quercetin-7-O-beta-D-glucopyranoside | Flavonoids | 1.60 | 0.05 | -1.22 |
| H20_N vs H20_CK | Suberic acid | Fatty Acyls | 1.72 | 0.02 | -1.18 |
| H20_N vs H20_CK | Catalpalactone | Sesquiterpenoids | 1.91 | 0.00 | -1.14 |
| H20_N vs H20_CK | Capsanthin | Terpene | 1.63 | 0.04 | -1.12 |

| | | | | | |
|---|---|---|---|---|---|
| H20_N vs H20_CK | 3-Hydroxy-4-methoxycinnamic acid | Phenylpropanoids | 1.75 | 0.02 | -1.12 |
| H20_N vs H20_CK | Isoscoparin | Flavonoids | 1.77 | 0.01 | -1.09 |
| H20_N vs H20_CK | Dalbergioidin | Flavonoids | 1.91 | 0.00 | -1.09 |
| H20_N vs H20_CK | Calystegine A3 | Alkaloids | 1.61 | 0.04 | -1.06 |
| H20_N vs H20_CK | Cosmosiin | Flavonoids | 1.82 | 0.01 | -1.04 |
| H20_N vs H20_CK | Eriodictyol | flavonoids | 1.83 | 0.01 | -1.01 |
| H20_N vs H20_CK | Quercetin 3-O-neohesperidoside | Flavonoids | 1.63 | 0.04 | -1.00 |
| H20_N vs H20_CK | 2-Picolinic acid | Organic acids | 1.59 | 0.05 | -1.00 |
| H20_N vs H20_CK | Sakuranetin | Flavonoids | 1.72 | 0.02 | -0.95 |
| H20_N vs H20_CK | Reserpine | Alkaloids | 1.69 | 0.03 | -0.92 |
| H20_N vs H20_CK | 2-(4-Methoxybenzal)acetophenone | Flavonoids | 1.58 | 0.05 | -0.92 |
| H20_N vs H20_CK | 4-Hydroxybenzoic acid | Phenols | 1.76 | 0.01 | -0.90 |
| H20_N vs H20_CK | Alizarin 2-methyl ether | Anthraquinones | 1.68 | 0.03 | -0.90 |
| H20_N vs H20_CK | N5-(L-1-Carboxyethyl)-L-ornithine | Organic acids | 1.61 | 0.04 | -0.86 |
| H20_N vs H20_CK | "1H-Indole-2,3-dione" | Indoles and derivatives | 1.87 | 0.00 | -0.85 |
| H20_N vs H20_CK | DL-Benzylsuccinic acid | Phenylpropanoids | 1.70 | 0.02 | -0.83 |
| H20_N vs H20_CK | Calycosin | Flavonoids | 1.62 | 0.04 | -0.74 |
| H20_N vs H20_CK | "(R)-2-Hydroxy-2H-1,4-benzoxazin-3(4H)-one" | Benzoxazines | 1.72 | 0.02 | -0.70 |
| H20_N vs H20_CK | Kaurenoic acid | Diterpenoids | 1.69 | 0.03 | -0.69 |
| H20_N vs H20_CK | Rutin | flavonoids | 1.75 | 0.02 | -0.68 |
| H20_N vs H20_CK | (+)-Afzelechin | Flavonoids | 1.82 | 0.01 | -0.67 |
| H20_N vs H20_CK | Glucofrangulin B | Anthraquinones | 1.87 | 0.00 | -0.66 |
| H20_N vs H20_CK | Ferulic acid | Phenylpropanoids | 1.76 | 0.01 | -0.63 |
| H20_N vs H20_CK | 2-Deoxyinosine | Nucleotide and its derivates | 1.71 | 0.02 | -0.63 |
| H20_N vs H20_CK | Amygdalin | Phenols | 1.62 | 0.04 | -0.61 |

| | | | | | |
|---|---|---|---|---|---|
| H20_N vs H20_CK | N-Feruloyl putrescine | Phenolamides | 1.61 | 0.04 | 0.66 |
| H20_N vs H20_CK | Indole-3-carboxaldehyde | Indoles and derivatives | 1.66 | 0.03 | 0.73 |
| H20_N vs H20_CK | 7-Methylxanthine | Nucleotide and its derivates | 1.60 | 0.05 | 0.80 |
| H20_N vs H20_CK | 3-Methylxanthine | Nucleotide and its derivates | 1.77 | 0.01 | 0.80 |
| H20_N vs H20_CK | Clovin | Flavonoids | 1.79 | 0.01 | 0.90 |
| H20_N vs H20_CK | "5-Tricosyl-1,3-benzenediol" | Phenols | 1.73 | 0.02 | 0.90 |
| H20_N vs H20_CK | Lactupicrin | Sesquiterpenoids | 1.79 | 0.01 | 0.91 |
| H20_N vs H20_CK | Flavonol | Flavonoids | 1.72 | 0.02 | 0.97 |
| H20_N vs H20_CK | Oleocanthal | Phenols | 1.78 | 0.01 | 1.04 |
| H20_N vs H20_CK | Alternariol | Phenols | 1.72 | 0.02 | 1.05 |
| H20_N vs H20_CK | L-Malic acid | Hydroxy acids and derivatives | 1.77 | 0.01 | 1.10 |
| H20_N vs H20_CK | 2(3H)-Benzothiazolethione | Benzothiazoles | 1.60 | 0.05 | 1.15 |
| H20_N vs H20_CK | N-gamma-Acetyl-N-2-Formyl-5-methoxykynurenamine | Amino acid and derivatives | 1.61 | 0.04 | 1.16 |
| H20_N vs H20_CK | Arachidonic acid | Fatty Acyls | 1.70 | 0.02 | 1.22 |
| H20_N vs H20_CK | Dihydrokavain | Phenols | 1.70 | 0.02 | 1.34 |
| H20_N vs H20_CK | Aloeemodin | Anthraquinones | 1.59 | 0.05 | 1.45 |
| H20_N vs H20_CK | Bergamotine | Coumarins | 1.72 | 0.02 | 1.67 |
| H20_N vs H20_CK | Panaxynol | Miscellaneous | 1.81 | 0.01 | 1.83 |
| H20_N vs H20_CK | Mannose 6-phosphate | Organooxygen compounds | 1.82 | 0.01 | 1.90 |
| H20_N vs H20_CK | Desmethylxanthohumol | Chalcones | 1.66 | 0.03 | 1.93 |
| H20_N vs H20_CK | Amabiline | Alkaloids | 1.84 | 0.00 | 2.03 |
| H20_N vs H20_CK | Octadecanamide | Fatty Acyls | 1.83 | 0.01 | 2.14 |
| H20_N vs H20_CK | Oleic acid | Fatty Acyls | 1.86 | 0.00 | 2.20 |
| H20_N vs H20_CK | 4-Hydroxyphenylacetylglutamic acid | Organic acids | 1.88 | 0.00 | 2.21 |
| H20_N vs H20_CK | Forskolin | Diterpenoids | 1.63 | 0.04 | 2.27 |

| | | | | | |
|---|---|---|---|---|---|
| H20_N vs H20_CK | Miltirone | Diterpenoids | 1.74 | 0.02 | 2.34 |
| H20_N vs H20_CK | Glucose 1-phosphate | Organooxygen compounds | 1.88 | 0.00 | 2.34 |
| H20_N vs H20_CK | Artemisinin | Sesquiterpenoids | 1.76 | 0.01 | 2.35 |
| H20_N vs H20_CK | Octyl Gallate | Phenols | 1.81 | 0.01 | 2.44 |
| H20_N vs H20_CK | 7-(4-Hydroxyphenyl)-1-phenyl-4-hepten-3-one | Phenols | 1.63 | 0.04 | 2.47 |
| H20_N vs H20_CK | Oleamide | Fatty Acyls | 1.80 | 0.01 | 2.50 |
| H20_N vs H20_CK | Glycerophosphocholine | Cholines | 1.79 | 0.01 | 2.66 |
| H20_N vs H20_CK | Palmitic acid | Lipids | 1.69 | 0.03 | 2.75 |

**Table S2.** Classification of NIF-induced differentially accumulated metabolites in the seedings of H01 and H20 inbred lines.

| Inbred lines name | Compound name | CLASS | $log_2$ FC (CK vs N) | Accumulation |
|---|---|---|---|---|
| H01 | Mesembrine | Alkaloids | -1.7828 | Down |
| H01 | Hypotaurine | Alkaloids | -1.3716 | Down |
| H01 | Tombozine | Alkaloids | -1.1234 | Down |
| H01 | Neolitsine | Alkaloids | -1.0728 | Down |
| H01 | Tropine acetate | Alkaloids | -1.0578 | Down |
| H01 | Gentioflavin | Alkaloids | -0.9446 | Down |
| H01 | "(-)-3-(3,4-Dihydroxyphenyl)-2-methylalanine" | Amino acid and its derivatives | -0.78594 | Down |
| H01 | "(R)-2-Hydroxy-2H-1,4-benzoxazin-3(4H)-one" | Benzoxazines | -0.86023 | Down |
| H01 | Palmitoylethanolamide | Carboximidic acids and derivatives | -0.90252 | Down |
| H01 | Wedelolactone | Coumarins | -2.2162 | Down |
| H01 | Stevioside | Diterpenoids | -2.4898 | Down |
| H01 | Suberic acid | Fatty Acyls | -1.8349 | Down |
| H01 | "Malvidin 3,5-diglucoside (Malvin)" | Flavonoids | -1.7736 | Down |
| H01 | Quercetin-3-O-sophoroside | Flavonoids | -1.3254 | Down |
| H01 | Isoscoparin | Flavonoids | -1.2114 | Down |
| H01 | Isosakuranetin | Flavonoids | -1.1687 | Down |
| H01 | Morin | Flavonoids | -0.97487 | Down |
| H01 | "2,3,5,7-Tetrahydroxyflavone" | Flavonoids | -0.86334 | Down |
| H01 | Peonidin-3-glucoside | Flavonoids | -0.651 | Down |
| H01 | 7-Methylguanine | Imidazopyrimidines | -1.0248 | Down |
| H01 | Podophyllotoxinone | Lignans | -0.90665 | Down |

| | | | | |
|---|---|---|---|---|
| H01 | Delta-Nonalactone | Miscellaneous | -0.9833 | Down |
| H01 | 5-S-Methyl-5-thioadenosine | Nucleotide and its derivates | -0.70958 | Down |
| H01 | 2-Picolinic acid | Organic acids | -1.4469 | Down |
| H01 | Atranorin | Phenols | -1.3649 | Down |
| H01 | 4-Hydroxybenzaldehyde | Phenols | -1.3368 | Down |
| H01 | Gamma-Tocotrienol | Phenols | -1.2454 | Down |
| H01 | Protocatechuic acid | Phenols | -0.72769 | Down |
| H01 | Guaiacol | Phenols | -0.61231 | Down |
| H01 | Norbixin | Prenol lipids | -0.95958 | Down |
| H01 | Convallatoxin | Steroids | -0.83593 | Down |
| H01 | Calenduloside E | Triterpenoids | -0.94808 | Down |
| H01 | Riboflavine | Vitamins | -0.92816 | Down |
| H01 | Allocryptopine | Alkaloids | 1.1733 | Up |
| H01 | Amabiline | Alkaloids | 1.5452 | Up |
| H01 | 5-Aminovaleric acid | Amino acid and derivatives | 1.2588 | Up |
| H01 | 5-Hydroxylysine | Amino acid and its derivatives | 0.89398 | UP |
| H01 | 2-Deoxyribose 5-phosphate | Carbohydrates | 3.8643 | UP |
| H01 | N-Acetylornithine | Carboxylic acids and derivatives | 1.7091 | UP |
| H01 | Desmethylxanthohumol | Chalcones | 1.3317 | UP |
| H01 | Miltirone | Diterpenoids | 1.7087 | Up |
| H01 | "5,6-DHET" | Fatty Acyls | 0.7923 | Up |
| H01 | "8,9-DiHETrE" | Fatty Acyls | 1.0636 | Up |
| H01 | Octadecanamide | Fatty Acyls | 1.4169 | Up |
| H01 | Oleamide | Fatty Acyls | 1.4889 | UP |
| H01 | Oleic acid | Fatty Acyls | 1.6216 | UP |

| | | | | |
|---|---|---|---|---|
| H01 | Chrysoeriol 7-apiosylglucoside | Flavonoids | 0.67123 | Up |
| H01 | Clovin | Flavonoids | 1.0378 | Up |
| H01 | Farrerol | Flavonoids | 1.8028 | UP |
| H01 | Quercetin | Flavonoids | 2.4031 | UP |
| H01 | Homoorientin | Flavonoids | 4.8747 | Up |
| H01 | 4-Demethylpodophyllotoxin | Lignans | 2.0716 | UP |
| H01 | Savinin | Lignans | 2.4122 | Up |
| H01 | Palmitic acid | Lipids | 2.0957 | Up |
| H01 | beta-Cryptoxanthin | Miscellaneous | 0.67499 | UP |
| H01 | Phytic acid | Miscellaneous | 1.6537 | UP |
| H01 | Bornyl acetate | Monoterpenoids | 0.66962 | UP |
| H01 | "2,6-Dimethyl-7-octene-2,3,6-triol" | Monoterpenoids | 1.7239 | UP |
| H01 | Pyrophosphate | Non-metal oxoanionic compounds | 1.0382 | UP |
| H01 | Mannose 6-phosphate | Organooxygen compounds | 1.3679 | Up |
| H01 | Glucose 1-phosphate | Organooxygen compounds | 1.7149 | Up |
| H01 | N-Feruloyl putrescine | Phenolamides | 0.58505 | Up |
| H01 | Moracin C | Phenols | 0.69546 | Up |
| H01 | Encecalin | Phenols | 0.75345 | UP |
| H01 | Octyl Gallate | Phenols | 1.626 | Up |
| H01 | 4-Hydroxyphenyl-2-propionic acid | Phenylpropanoic acids | 1.2081 | UP |
| H01 | 4-Hydroxyphenylacetylglutamic acid | Organic acids | 1.7587 | Up |
| H01 | Pterosin D | Sesquiterpenoids | 1.0773 | UP |
| H01 | Artemisinin | Sesquiterpenoids | 1.8858 | Up |
| H01 | 6-Deoxyjacareubin | Xanthones | 1.3789 | UP |

| | | | | |
|---|---|---|---|---|
| H20 | Ganoderic acid F | Triterpenoids | -5.1906 | Down |
| H20 | Oxychelerythrine | Alkaloids | -2.6936 | Down |
| H20 | Naringenin | Flavonoids | -2.4148 | Down |
| H20 | Hesperetin | Flavonoids | -2.3728 | Down |
| H20 | "2,3,5,7-Tetrahydroxyflavone" | Flavonoids | -2.3199 | Down |
| H20 | Quinapril | Carboxylic acids and derivatives | -2.2911 | Down |
| H20 | Homoeriodictyol | Flavonoids | -2.2574 | Down |
| H20 | Enoxacin | Diazanaphthalenes | -2.2191 | Down |
| H20 | Canadine | Alkaloids | -2.0495 | Down |
| H20 | Theaflavin | Flavonoids | -2.0494 | Down |
| H20 | Diosmin | Flavonoids | -2.0329 | Down |
| H20 | Isosakuranetin | Flavonoids | -1.9463 | Down |
| H20 | Norbixin | Prenol lipids | -1.8746 | Down |
| H20 | vitamin K2 | Vitamins | -1.8734 | Down |
| H20 | Nandrolone | Steroids and steroid derivatives | -1.8339 | Down |
| H20 | Piperlonguminine | Alkaloids | -1.7938 | Down |
| H20 | Kaempferol-3-O-rutinoside | Flavonoids | -1.7483 | Down |
| H20 | 7-Hydroxyflavone | Flavonoids | -1.7233 | Down |
| H20 | beta-Cryptoxanthin | Miscellaneous | -1.7058 | Down |
| H20 | (1R)-(-)-Menthyl acetate | Monoterpenoids | -1.5859 | Down |
| H20 | 6-(Furfurylamino)purine | Alkaloids | -1.5239 | Down |
| H20 | Bruceine D | Diterpenoids | -1.4254 | Down |
| H20 | Trehalose 6-phosphate | Carbohydrates | -1.4098 | Down |
| H20 | Acacetin | Flavonoids | -1.3937 | Down |
| H20 | Morin | Flavonoids | -1.3204 | Down |

| | | | | |
|---|---|---|---|---|
| H20 | Dyclonine | Organooxygen compounds | -1.2816 | Down |
| H20 | 1-Caffeoylquinic acid | Phenylpropanoids | -1.259 | Down |
| H20 | Narcissoside | Flavonoids | -1.2574 | Down |
| H20 | Quercetin-7-O-beta-D-glucopyranoside | Flavonoids | -1.223 | Down |
| H20 | Suberic acid | Fatty Acyls | -1.1798 | Down |
| H20 | Catalpalactone | Sesquiterpenoids | -1.1351 | Down |
| H20 | Capsanthin | Terpene | -1.1228 | Down |
| H20 | 3-Hydroxy-4-methoxycinnamic acid | Phenylpropanoids | -1.1152 | Down |
| H20 | Isoscoparin | Flavonoids | -1.0884 | Down |
| H20 | Dalbergioidin | Flavonoids | -1.0868 | Down |
| H20 | Calystegine A3 | Alkaloids | -1.0577 | Down |
| H20 | Cosmosiin | Flavonoids | -1.0422 | Down |
| H20 | Eriodictyol | Flavonoids | -1.0078 | Down |
| H20 | Quercetin 3-O-neohesperidoside | Flavonoids | -1.0009 | Down |
| H20 | 2-Picolinic acid | Organic acids | -0.99993 | Down |
| H20 | Sakuranetin | Flavonoids | -0.95049 | Down |
| H20 | Reserpine | Alkaloids | -0.92066 | Down |
| H20 | 2-(4-Methoxybenzal)acetophenone | Flavonoids | -0.9185 | Down |
| H20 | 4-Hydroxybenzoic acid | Phenols | -0.90234 | Down |
| H20 | Alizarin 2-methyl ether | Anthraquinones | -0.90142 | Down |
| H20 | N5-(L-1-Carboxyethyl)-L-ornithine | Organic acids | -0.86314 | Down |
| H20 | "1H-Indole-2,3-dione" | Indoles and derivatives | -0.85049 | Down |
| H20 | DL-Benzylsuccinic acid | Phenylpropanoids | -0.83486 | Down |
| H20 | Calycosin | Flavonoids | -0.73676 | Down |
| H20 | "(R)-2-Hydroxy-2H-1,4-benzoxazin-3(4H)-one" | Benzoxazines | -0.69991 | Down |

| | | | | |
|---|---|---|---|---|
| H20 | Kaurenoic acid | Diterpenoids | -0.69051 | Down |
| H20 | Rutin | Flavonoids | -0.67513 | Down |
| H20 | (+)-Afzelechin | Flavonoids | -0.66701 | Down |
| H20 | Glucofrangulin B | Anthraquinones | -0.65532 | Down |
| H20 | Ferulic acid | Phenylpropanoids | -0.63097 | Down |
| H20 | 2-Deoxyinosine | Nucleotide and its derivates | -0.62573 | Down |
| H20 | Amygdalin | Phenols | -0.60636 | Down |
| H20 | N-Feruloyl putrescine | Phenolamides | 0.65515 | Up |
| H20 | Indole-3-carboxaldehyde | Indoles and derivatives | 0.73088 | Up |
| H20 | 7-Methylxanthine | Nucleotide and its derivates | 0.79594 | Up |
| H20 | 3-Methylxanthine | Nucleotide and its derivates | 0.80091 | Up |
| H20 | Clovin | Flavonoids | 0.8951 | Up |
| H20 | "5-Tricosyl-1,3-benzenediol" | Phenols | 0.89625 | Up |
| H20 | Lactupicrin | Sesquiterpenoids | 0.91465 | Up |
| H20 | Flavonol | Flavonoids | 0.96627 | Up |
| H20 | Oleocanthal | Phenols | 1.037 | Up |
| H20 | Alternariol | Phenols | 1.0467 | Up |
| H20 | L-Malic acid | Hydroxy acids and derivatives | 1.1049 | Up |
| H20 | 2(3H)-Benzothiazolethione | Benzothiazoles | 1.1487 | Up |
| H20 | N-gamma-Acetyl-N-2-Formyl-5-methoxykynurenamine | Amino acid and derivatives | 1.1617 | Up |
| H20 | Arachidonic acid | Fatty Acyls | 1.2158 | Up |
| H20 | Dihydrokavain | Phenols | 1.3388 | Up |
| H20 | Aloeemodin | Anthraquinones | 1.4456 | Up |
| H20 | Bergamotine | Coumarins | 1.6674 | Up |

| | | | | |
|---|---|---|---|---|
| H20 | Panaxynol | Miscellaneous | 1.8296 | Up |
| H20 | Mannose 6-phosphate | Organooxygen compounds | 1.9001 | Up |
| H20 | Desmethylxanthohumol | Chalcones | 1.9284 | Up |
| H20 | Amabiline | Alkaloids | 2.034 | Up |
| H20 | Octadecanamide | Fatty Acyls | 2.1365 | Up |
| H20 | Oleic acid | Fatty Acyls | 2.1985 | Up |
| H20 | 4-Hydroxyphenylacetylglutamic acid | Organic acids | 2.2104 | Up |
| H20 | Forskolin | Diterpenoids | 2.2661 | Up |
| H20 | Miltirone | Diterpenoids | 2.3354 | Up |
| H20 | Glucose 1-phosphate | Organooxygen compounds | 2.339 | Up |
| H20 | Artemisinin | Sesquiterpenoids | 2.3487 | Up |
| H20 | Octyl Gallate | Phenols | 2.4436 | Up |
| H20 | 7-(4-Hydroxyphenyl)-1-phenyl-4-hepten-3-one | Phenols | 2.469 | Up |
| H20 | Oleamide | Fatty Acyls | 2.496 | Up |
| H20 | Glycerophosphocholine | Cholines | 2.6648 | Up |
| H20 | Palmitic acid | Lipids | 2.751 | Up |

**Table S3**. The NIF-induced differentially accumulated metabolites in the seedings of H01 and H20 inbred lines.

| Inbred line name | Compound name | CLASS | $log_2$ FC (CK vs N) | $log_2$ FC (CK vs GN) |
|---|---|---|---|---|
| H01 | Mesembrine | Alkaloids | -1.7828 | NA |
| H01 | Hypotaurine | Alkaloids | -1.3716 | NA |
| H01 | Tombozine | Alkaloids | -1.1234 | NA |
| H01 | Neolitsine | Alkaloids | -1.0728 | NA |
| H01 | Tropine acetate | Alkaloids | -1.0578 | NA |
| H01 | Gentioflavin | Alkaloids | -0.9446 | NA |
| H01 | Allocryptopine | Alkaloids | 1.1733 | NA |
| H01 | Amabiline | Alkaloids | 1.5452 | NA |
| H01 | 5-Aminovaleric acid | Amino acid and derivatives | 1.2588 | 0.73351 |
| H01 | "(-)-3-(3,4-Dihydroxyphenyl)-2-methylalanine" | Amino acid and its derivatives | -0.78594 | NA |
| H01 | 5-Hydroxylysine | Amino acid and its derivatives | 0.89398 | NA |
| H01 | "(R)-2-Hydroxy-2H-1,4-benzoxazin-3(4H)-one" | Benzoxazines | -0.86023 | 0.68283 |
| H01 | 2-Deoxyribose 5-phosphate | Carbohydrates | 3.8643 | NA |
| H01 | Palmitoylethanolamide | Carboximidic acids and derivatives | -0.90252 | NA |
| H01 | N-Acetylornithine | Carboxylic acids and derivatives | 1.7091 | NA |
| H01 | Desmethylxanthohumol | Chalcones | 1.3317 | NA |
| H01 | Wedelolactone | Coumarins | -2.2162 | NA |
| H01 | Stevioside | Diterpenoids | -2.4898 | -1.9085 |
| H01 | Miltirone | Diterpenoids | 1.7087 | NA |
| H01 | Suberic acid | Fatty Acyls | -1.8349 | -1.1082 |
| H01 | "5,6-DHET" | Fatty Acyls | 0.7923 | NA |
| H01 | "8,9-DiHETrE" | Fatty Acyls | 1.0636 | NA |

| | | | | |
|---|---|---|---|---|
| H01 | Octadecanamide | Fatty Acyls | 1.4169 | NA |
| H01 | Oleamide | Fatty Acyls | 1.4889 | NA |
| H01 | Oleic acid | Fatty Acyls | 1.6216 | NA |
| H01 | "Malvidin 3,5-diglucoside (Malvin)" | Flavonoids | -1.7736 | -0.73081 |
| H01 | Quercetin-3-O-sophoroside | Flavonoids | -1.3254 | NA |
| H01 | Isoscoparin | Flavonoids | -1.2114 | NA |
| H01 | Isosakuranetin | Flavonoids | -1.1687 | NA |
| H01 | Morin | Flavonoids | -0.97487 | NA |
| H01 | "2,3,5,7-Tetrahydroxyflavone" | Flavonoids | -0.86334 | NA |
| H01 | Peonidin-3-glucoside | Flavonoids | -0.651 | NA |
| H01 | Chrysoeriol 7-apiosylglucoside | Flavonoids | 0.67123 | NA |
| H01 | Clovin | Flavonoids | 1.0378 | 1.0148 |
| H01 | Farrerol | Flavonoids | 1.8028 | NA |
| H01 | Quercetin | Flavonoids | 2.4031 | 1.9081 |
| H01 | Homoorientin | Flavonoids | 4.8747 | NA |
| H01 | 7-Methylguanine | Imidazopyrimidines | -1.0248 | NA |
| H01 | Podophyllotoxinone | Lignans | -0.90665 | NA |
| H01 | 4-Demethylpodophyllotoxin | Lignans | 2.0716 | NA |
| H01 | Savinin | Lignans | 2.4122 | NA |
| H01 | Palmitic acid | Lipids | 2.0957 | NA |
| H01 | Delta-Nonalactone | Miscellaneous | -0.9833 | NA |
| H01 | beta-Cryptoxanthin | Miscellaneous | 0.67499 | NA |
| H01 | Phytic acid | Miscellaneous | 1.6537 | NA |
| H01 | Bornyl acetate | Monoterpenoids | 0.66962 | NA |
| H01 | "2,6-Dimethyl-7-octene-2,3,6-triol" | Monoterpenoids | 1.7239 | NA |

| | | | | |
|---|---|---|---|---|
| H01 | Pyrophosphate | Non-metal oxoanionic compounds | 1.0382 | NA |
| H01 | 5-S-Methyl-5-thioadenosine | Nucleotide and its derivates | -0.70958 | NA |
| H01 | 2-Picolinic acid | Organic acids | -1.4469 | NA |
| H01 | Mannose 6-phosphate | Organooxygen compounds | 1.3679 | NA |
| H01 | Glucose 1-phosphate | Organooxygen compounds | 1.7149 | NA |
| H01 | N-Feruloyl putrescine | Phenolamides | 0.58505 | NA |
| H01 | Atranorin | Phenols | -1.3649 | NA |
| H01 | 4-Hydroxybenzaldehyde | Phenols | -1.3368 | NA |
| H01 | Gamma-Tocotrienol | Phenols | -1.2454 | NA |
| H01 | Protocatechuic acid | Phenols | -0.72769 | -0.68553 |
| H01 | Guaiacol | Phenols | -0.61231 | 0.80313 |
| H01 | Moracin C | Phenols | 0.69546 | NA |
| H01 | Encecalin | Phenols | 0.75345 | NA |
| H01 | Octyl Gallate | Phenols | 1.626 | NA |
| H01 | 4-Hydroxyphenyl-2-propionic acid | Phenylpropanoic acids | 1.2081 | NA |
| H01 | Norbixin | Prenol lipids | -0.95958 | NA |
| H01 | Pterosin D | Sesquiterpenoids | 1.0773 | 1.3764 |
| H01 | Artemisinin | Sesquiterpenoids | 1.8858 | NA |
| H01 | Convallatoxin | Steroids | -0.83593 | NA |
| H01 | Calenduloside E | Triterpenoids | -0.94808 | -1.6809 |
| H01 | Riboflavine | Vitamins | -0.92816 | NA |
| H01 | 6-Deoxyjacareubin | Xanthones | 1.3789 | NA |
| H01 | 4-Hydroxyphenylacetylglutamic acid | Organic acids | 1.7587 | NA |
| H20 | Oxychelerythrine | Alkaloids | -2.6936 | NA |
| H20 | Canadine | Alkaloids | -2.0495 | -2.2495 |

| | | | | |
|---|---|---|---|---|
| H20 | Piperlonguminine | Alkaloids | -1.7938 | NA |
| H20 | 6-(Furfurylamino)purine | Alkaloids | -1.5239 | -1.5213 |
| H20 | Calystegine A3 | Alkaloids | -1.0577 | NA |
| H20 | Reserpine | Alkaloids | -0.92066 | -3.184 |
| H20 | Amabiline | Alkaloids | 2.034 | NA |
| H20 | N-gamma-Acetyl-N-2-Formyl-5-methoxykynurenamine | Amino acid and derivatives | 1.1617 | NA |
| H20 | Alizarin 2-methyl ether | Anthraquinones | -0.90142 | NA |
| H20 | Glucofrangulin B | Anthraquinones | -0.65532 | -1.1516 |
| H20 | Aloeemodin | Anthraquinones | 1.4456 | NA |
| H20 | 2(3H)-Benzothiazolethione | Benzothiazoles | 1.1487 | NA |
| H20 | "(R)-2-Hydroxy-2H-1,4-benzoxazin-3(4H)-one" | Benzoxazines | -0.69991 | NA |
| H20 | Trehalose 6-phosphate | Carbohydrates | -1.4098 | NA |
| H20 | Quinapril | Carboxylic acids and derivatives | -2.2911 | -1.6363 |
| H20 | Desmethylxanthohumol | Chalcones | 1.9284 | NA |
| H20 | Glycerophosphocholine | Cholines | 2.6648 | NA |
| H20 | Bergamotine | Coumarins | 1.6674 | NA |
| H20 | Enoxacin | Diazanaphthalenes | -2.2191 | NA |
| H20 | Bruceine D | Diterpenoids | -1.4254 | -2.3376 |
| H20 | Kaurenoic acid | Diterpenoids | -0.69051 | -0.66854 |
| H20 | Forskolin | Diterpenoids | 2.2661 | NA |
| H20 | Miltirone | Diterpenoids | 2.3354 | NA |
| H20 | Suberic acid | Fatty Acyls | -1.1798 | 0.7198 |
| H20 | Arachidonic acid | Fatty Acyls | 1.2158 | NA |
| H20 | Octadecanamide | Fatty Acyls | 2.1365 | NA |

| | | | | |
|---|---|---|---|---|
| H20 | Oleic acid | Fatty Acyls | 2.1985 | NA |
| H20 | Oleamide | Fatty Acyls | 2.496 | NA |
| H20 | Naringenin | Flavonoids | -2.4148 | NA |
| H20 | Hesperetin | Flavonoids | -2.3728 | NA |
| H20 | "2,3,5,7-Tetrahydroxyflavone" | Flavonoids | -2.3199 | -1.8169 |
| H20 | Homoeriodictyol | Flavonoids | -2.2574 | -0.82729 |
| H20 | Theaflavin | Flavonoids | -2.0494 | -1.7718 |
| H20 | Diosmin | Flavonoids | -2.0329 | -1.2404 |
| H20 | Isosakuranetin | Flavonoids | -1.9463 | -1.2202 |
| H20 | Kaempferol-3-O-rutinoside | Flavonoids | -1.7483 | -0.83891 |
| H20 | 7-Hydroxyflavone | Flavonoids | -1.7233 | -2.0049 |
| H20 | Acacetin | Flavonoids | -1.3937 | NA |
| H20 | Morin | Flavonoids | -1.3204 | -0.83212 |
| H20 | Narcissoside | Flavonoids | -1.2574 | NA |
| H20 | Quercetin-7-O-beta-D-glucopyranoside | Flavonoids | -1.223 | -1.3501 |
| H20 | Isoscoparin | Flavonoids | -1.0884 | NA |
| H20 | Dalbergioidin | Flavonoids | -1.0868 | -1.1983 |
| H20 | Cosmosiin | Flavonoids | -1.0422 | NA |
| H20 | Eriodictyol | Flavonoids | -1.0078 | NA |
| H20 | Quercetin 3-O-neohesperidoside | Flavonoids | -1.0009 | -1.5335 |
| H20 | Sakuranetin | Flavonoids | -0.95049 | -0.65665 |
| H20 | 2-(4-Methoxybenzal)acetophenone | Flavonoids | -0.9185 | NA |
| H20 | Calycosin | Flavonoids | -0.73676 | NA |
| H20 | Rutin | Flavonoids | -0.67513 | -0.75862 |
| H20 | (+)-Afzelechin | Flavonoids | -0.66701 | -0.62651 |

| | | | | |
|---|---|---|---|---|
| H20 | Clovin | Flavonoids | 0.8951 | NA |
| H20 | Flavonol | Flavonoids | 0.96627 | NA |
| H20 | L-Malic acid | Hydroxy acids and derivatives | 1.1049 | NA |
| H20 | "1H-Indole-2,3-dione" | Indoles and derivatives | -0.85049 | -1.3336 |
| H20 | Indole-3-carboxaldehyde | Indoles and derivatives | 0.73088 | NA |
| H20 | Palmitic acid | Lipids | 2.751 | NA |
| H20 | beta-Cryptoxanthin | Miscellaneous | -1.7058 | NA |
| H20 | Panaxynol | Miscellaneous | 1.8296 | NA |
| H20 | (1R)-(-)-Menthyl acetate | Monoterpenoids | -1.5859 | NA |
| H20 | 2-Deoxyinosine | Nucleotide and its derivates | -0.62573 | NA |
| H20 | 7-Methylxanthine | Nucleotide and its derivates | 0.79594 | NA |
| H20 | 3-Methylxanthine | Nucleotide and its derivates | 0.80091 | NA |
| H20 | 2-Picolinic acid | Organic acids | -0.99993 | NA |
| H20 | Dyclonine | Organooxygen compounds | -1.2816 | -2.1396 |
| H20 | Mannose 6-phosphate | Organooxygen compounds | 1.9001 | NA |
| H20 | Glucose 1-phosphate | Organooxygen compounds | 2.339 | NA |
| H20 | N-Feruloyl putrescine | Phenolamides | 0.65515 | NA |
| H20 | 4-Hydroxybenzoic acid | Phenols | -0.90234 | -0.69341 |
| H20 | Amygdalin | Phenols | -0.60636 | NA |
| H20 | "5-Tricosyl-1,3-benzenediol" | Phenols | 0.89625 | NA |
| H20 | Oleocanthal | Phenols | 1.037 | NA |
| H20 | Alternariol | Phenols | 1.0467 | NA |
| H20 | Dihydrokavain | Phenols | 1.3388 | NA |
| H20 | Octyl Gallate | Phenols | 2.4436 | NA |
| H20 | 7-(4-Hydroxyphenyl)-1-phenyl-4-hepten-3-one | Phenols | 2.469 | NA |

| | | | | |
|---|---|---|---|---|
| H20 | 1-Caffeoylquinic acid | Phenylpropanoids | -1.259 | -2.0857 |
| H20 | 3-Hydroxy-4-methoxycinnamic acid | Phenylpropanoids | -1.1152 | NA |
| H20 | DL-Benzylsuccinic acid | Phenylpropanoids | -0.83486 | -1.4593 |
| H20 | Ferulic acid | Phenylpropanoids | -0.63097 | -0.8203 |
| H20 | Norbixin | Prenol lipids | -1.8746 | -1.5254 |
| H20 | Catalpalactone | Sesquiterpenoids | -1.1351 | -0.90594 |
| H20 | Lactupicrin | Sesquiterpenoids | 0.91465 | NA |
| H20 | Artemisinin | Sesquiterpenoids | 2.3487 | NA |
| H20 | Nandrolone | Steroids and steroid derivatives | -1.8339 | NA |
| H20 | Capsanthin | Terpene | -1.1228 | -1.3253 |
| H20 | Ganoderic acid F | Triterpenoids | -5.1906 | NA |
| H20 | vitamin K2 | Vitamins | -1.8734 | -0.63607 |
| H20 | N5-(L-1-Carboxyethyl)-L-ornithine | Organic acids | -0.86314 | -0.80443 |
| H20 | 4-Hydroxyphenylacetylglutamic acid | Organic acids | 2.2104 | NA |

**Table S4**. The GO-induced differentially accumulated metabolites in the seedings of H01 and H20 inbred lines under NIF treatment.

| **Inbred line name** | **Compound name** | **CLASS** | **$log_2$ FC (GN vs N)** | **Accumulation** |
|---|---|---|---|---|
| H01 | Petasitenine | Alkaloids | -4.8516 | Down |
| H01 | Amabiline | Alkaloids | -2.2776 | Down |
| H01 | Symlandine | Alkaloids | -1.4335 | Down |
| H01 | Methylisopelletierine | Alkaloids | -1.1263 | Down |
| H01 | Mitraphylline | Alkaloids | -1.0828 | Down |
| H01 | Desmethylxanthohumol | Chalcones | -1.875 | Down |
| H01 | Glycerophosphocholine | Cholines | -3.019 | Down |
| H01 | Bergamotine | Coumarins | -2.0778 | Down |
| H01 | Miltirone | Diterpenoids | -2.288 | Down |
| H01 | Oleic acid | Fatty Acyls | -2.4751 | Down |
| H01 | "8,9-DiHETrE" | Fatty Acyls | -2.3778 | Down |
| H01 | Octadecanamide | Fatty Acyls | -2.2313 | Down |
| H01 | Oleamide | Fatty Acyls | -1.9256 | Down |
| H01 | Cyanidin-3-O-rhamnoside chloride | Flavonoids | -0.92369 | Down |
| H01 | Chrysoeriol 7-apiosylglucoside | Flavonoids | -0.74061 | Down |
| H01 | Palmitic acid | Lipids | -2.4059 | Down |
| H01 | Aristolochic acid A | Miscellaneous | -3.8776 | Down |
| H01 | Panaxynol | Miscellaneous | -2.4922 | Down |
| H01 | Phosphonoacetate | Organic acids | -0.68183 | Down |
| H01 | Glucose 1-phosphate | Organooxygen compounds | -2.2133 | Down |
| H01 | Mannose 6-phosphate | Organooxygen compounds | -1.8035 | Down |
| H01 | 7-(4-Hydroxyphenyl)-1-phenyl-4-hepten-3-one | Phenols | -2.8435 | Down |

| | | | | |
|---|---|---|---|---|
| H01 | Octyl Gallate | Phenols | -2.7163 | Down |
| H01 | Moracin C | Phenols | -0.93513 | Down |
| H01 | Artemisinin | Sesquiterpenoids | -2.4481 | Down |
| H01 | Calenduloside E | Triterpenoids | -0.73284 | Down |
| H01 | 4-Hydroxyphenylacetylglutamic acid | Organic acids | -2.4879 | Down |
| H01 | Hordenine | Alkaloids | 0.60072 | Up |
| H01 | Desoxypeganine | Alkaloids | 0.61286 | Up |
| H01 | 1H-Indole-3-carboxylic acid | Alkaloids | 0.66037 | Up |
| H01 | Melatonin | Alkaloids | 0.95517 | Up |
| H01 | Dehydronuciferine | Alkaloids | 0.96755 | Up |
| H01 | Piperlonguminine | Alkaloids | 1.229 | Up |
| H01 | Mesembrine | Alkaloids | 1.6803 | Up |
| H01 | L-Isoleucine | Amino acid and derivatives | 1.0675 | Up |
| H01 | "(-)-3-(3,4-Dihydroxyphenyl)-2-methylalanine" | Amino acid and its derivatives | 0.86553 | Up |
| H01 | Cascaroside A | Anthraquinones | 1.3197 | Up |
| H01 | Benzocaine | Benzene and substituted derivatives | 1.0256 | Up |
| H01 | Formylanthranilic acid | Benzene and substituted derivatives | 1.2515 | Up |
| H01 | Dimethylbenzimidazole | Benzimidazoles | 0.91573 | Up |
| H01 | "(R)-2-Hydroxy-2H-1,4-benzoxazin-3(4H)-one" | Benzoxazines | 1.5431 | Up |
| H01 | "2,6-Dihydroxy 4-methoxydihydrochalcone" | Chalcones | 1.4948 | Up |
| H01 | Carbofuran | Coumarans | 0.77062 | Up |
| H01 | Tripdiolide | Diterpenoids | 1.0666 | Up |
| H01 | Suberic acid | Fatty Acyls | 0.72671 | Up |
| H01 | Stearidonic acid | Fatty Acyls | 1.2687 | Up |
| H01 | Arachidonic acid | Fatty Acyls | 2.2835 | Up |

| | | | | |
|---|---|---|---|---|
| H01 | Daidzein | Flavonoids | 0.69831 | Up |
| H01 | isoliquiritigenin | Flavonoids | 0.78787 | Up |
| H01 | Gossypetin | Flavonoids | 0.87793 | Up |
| H01 | Garbanzol | Flavonoids | 0.90664 | Up |
| H01 | Quercetin-3-O-glucuronide | Flavonoids | 1.0448 | Up |
| H01 | Pelargonidin | Flavonoids | 1.0494 | Up |
| H01 | Kaempferol-3-O-rutinoside | Flavonoids | 1.0722 | Up |
| H01 | Taxifolin | Flavonoids | 1.0814 | Up |
| H01 | Dalbergioidin | Flavonoids | 1.1101 | Up |
| H01 | Tricetin | Flavonoids | 1.1498 | Up |
| H01 | Isoscoparin | Flavonoids | 1.2437 | Up |
| H01 | "2,3,5,7-Tetrahydroxyflavone" | Flavonoids | 1.3303 | Up |
| H01 | Isosakuranetin | Flavonoids | 1.5115 | Up |
| H01 | Eriodictyol | Flavonoids | 1.5849 | Up |
| H01 | Apigenin | Flavonoids | 1.6714 | Up |
| H01 | Blumeatin | Flavonoids | 1.7175 | Up |
| H01 | Saponarin | Flavonoids | 1.7921 | Up |
| H01 | Quercetin-3-O-sophoroside | Flavonoids | 1.9163 | Up |
| H01 | Glycitein | Flavonoids | 2.927 | Up |
| H01 | Hesperetin | Flavonoids | 3.0461 | Up |
| H01 | Naringenin | Flavonoids | 3.1787 | Up |
| H01 | Homoeriodictyol | Flavonoids | 3.2181 | Up |
| H01 | Sinapyl alcohol | Hydroxycinnamoyl derivatives | 1.0838 | Up |
| H01 | 7-Methylguanine | Imidazopyrimidines | 1.2144 | Up |
| H01 | Podophyllotoxinone | Lignans | 0.73799 | Up |

| | | | | |
|---|---|---|---|---|
| H01 | Deoxypodophyllotoxin | Lignans | 1.0537 | Up |
| H01 | Yatein | Lignans | 1.2813 | Up |
| H01 | 5-S-Methyl-5-thioadenosine | Nucleotide and its derivates | 0.74167 | Up |
| H01 | Deoxyguanosine | Nucleotide and its derivates | 1.1205 | Up |
| H01 | 2-Picolinic acid | Organic acids | 1.4331 | Up |
| H01 | L-Arabitol | Organooxygen compounds | 0.68397 | Up |
| H01 | Glucoiberin | Organooxygen compounds | 1.164 | Up |
| H01 | "3,4-Dihydroxybenzaldehyde" | Phenols | 0.69753 | Up |
| H01 | "5-Tricosyl-1,3-benzenediol" | Phenols | 0.76595 | Up |
| H01 | Sesamol | Phenols | 0.89158 | Up |
| H01 | 3-Hydroxyphenylacetic acid | Phenols | 0.92486 | Up |
| H01 | N-(p-Hydroxyphenethyl)actinidine | Phenols | 1.0222 | Up |
| H01 | Atranorin | Phenols | 1.2814 | Up |
| H01 | Guaiacol | Phenols | 1.4154 | Up |
| H01 | 1-Acetoxychavicol acetate | Phenols | 1.7677 | Up |
| H01 | Oleocanthal | Phenols | 1.9512 | Up |
| H01 | "trans-3,5-Dimethoxy-4-hydroxy cinnamaldehydee" | Phenylpropanoids | 1.0634 | Up |
| H01 | Cinnamyl cinnamate | Phenylpropanoids | 1.4837 | Up |
| H01 | 3-Indolebutyric acid | phytohormone | 1.7798 | Up |
| H01 | Dihydrojasmonic Acid | phytohormone | 1.8805 | Up |
| H01 | Lutein | Prenol lipids | 1.7309 | Up |
| H01 | Norbixin | Prenol lipids | 1.7453 | Up |
| H01 | Psychosine | Sphingolipids | 1.581 | Up |
| H01 | Withaferin A | Steroids and steroid derivatives | 1.0417 | Up |
| H01 | Nandrolone | Steroids and steroid derivatives | 1.4002 | Up |

| | | | | |
|---|---|---|---|---|
| H01 | Loganin | Terpene | 1.5565 | Up |
| H01 | (-)-Salsoline | Tetrahydroisoquinolines | 0.75047 | Up |
| H01 | Betulinic acid | Triterpenoids | 1.0113 | Up |
| H01 | 11-Keto-beta-boswellic acid | Triterpenoids | 1.1002 | Up |
| H01 | Medicagenic acid | Triterpenoids | 1.6087 | Up |
| H01 | vitamin K2 | Vitamins | 0.71564 | Up |
| H01 | Riboflavine | Vitamins | 0.96023 | Up |
| H01 | Euxanthone | Xanthones | 1.0667 | Up |
| H01 | "cis-3-(Carboxy-ethyl)-3,5-cyclo-hexadiene-1,2-diol" | organic ester | 0.98096 | Up |
| H01 | O-Succinyl-L-homoserine | Amino acid and derivatives | 1.1286 | Up |
| H01 | N-D-Glucosylarylamine | Glucose | 1.3778 | Up |
| H20 | Fumitremorgin B | Alkaloids | -2.36 | Down |
| H20 | Reserpine | Alkaloids | -2.2633 | Down |
| H20 | Petasitenine | Alkaloids | -2.053 | Down |
| H20 | Hordenine | Alkaloids | -0.78146 | Down |
| H20 | 5-oxoproline | Amino acid and derivatives | -0.75335 | Down |
| H20 | Aloeemodin | Anthraquinones | -1.6729 | Down |
| H20 | N-Acetyl-L-glutamate 5-semialdehyde | Carboxylic acids and derivatives | -1.7542 | Down |
| H20 | Esculin | Coumarins | -0.89897 | Down |
| H20 | Samidin | Coumarins | -0.79943 | Down |
| H20 | Decursinol | Coumarins | -0.71006 | Down |
| H20 | Forskolin | Diterpenoids | -2.1954 | Down |
| H20 | Lathyrol | Diterpenoids | -1.0414 | Down |
| H20 | Dodecanedioic aicd | Fatty Acyls | -1.1152 | Down |

| | | | | |
|---|---|---|---|---|
| H20 | Saponarin | Flavonoids | -1.8094 | Down |
| H20 | Cimifugin | Flavonoids | -1.4257 | Down |
| H20 | Chrysoeriol 7-apiosylglucoside | Flavonoids | -0.95347 | Down |
| H20 | Rhoifolin | Flavonoids | -0.74593 | Down |
| H20 | Cynaroside | Flavonoids | -0.70554 | Down |
| H20 | Isovitexin | Flavonoids | -0.60944 | Down |
| H20 | L-Malic acid | Hydroxy acids and derivatives | -1.1064 | Down |
| H20 | 3-Methylxanthine | Nucleotide and its derivates | -1.298 | Down |
| H20 | Dyclonine | Organooxygen compounds | -0.85794 | Down |
| H20 | N-Feruloyl putrescine | Phenolamides | -0.92597 | Down |
| H20 | alpha-Tocopherol | Phenols | -2.8153 | Down |
| H20 | Gamma-Tocotrienol | Phenols | -1.7875 | Down |
| H20 | Sesamol | Phenols | -1.247 | Down |
| H20 | Dihydrokavain | Phenols | -1.0129 | Down |
| H20 | Protocatechuic acid | Phenols | -0.73554 | Down |
| H20 | 4-Hydroxyphenylacetic acid | Phenols | -0.70238 | Down |
| H20 | beta-Asarone | Phenylpropanoids | -1.0233 | Down |
| H20 | (+/-)-Jasmonic acid | phytohormone | -1.575 | Down |
| H20 | Folinic acid | Pteridines and derivatives | -1.4656 | Down |
| H20 | Picrotoxinin | Sesquiterpenoids | -2.7339 | Down |
| H20 | Parthenolide | Sesquiterpenoids | -0.99199 | Down |
| H20 | Lactupicrin | Sesquiterpenoids | -0.98422 | Down |
| H20 | Testosterone | Steroids | -1.1354 | Down |
| H20 | Tetrahymanol | Triterpenoids | -1.5116 | Down |
| H20 | Niloticin | Triterpenoids | -1.4525 | Down |

| | | | | |
|---|---|---|---|---|
| H20 | 3-Isomangostin hydrate | Xanthones | -0.96351 | Down |
| H20 | 5-Carboxyvanillic acid | Organic acids | -0.76002 | Down |
| H20 | Harmaline | Alkaloids | 0.72746 | Up |
| H20 | Scopolamine N-oxide hydrobromide | Alkaloids | 1.3103 | Up |
| H20 | Jervine | Alkaloids | 1.3491 | Up |
| H20 | Piperlonguminine | Alkaloids | 1.4449 | Up |
| H20 | L-Ornithine | Amino acid and derivatives | 0.6314 | Up |
| H20 | Acetyl tryptophan | Amino acid and derivatives | 0.8408 | Up |
| H20 | Diphenylamine | Benzene and substituted derivatives | 0.62603 | Up |
| H20 | Carbendazim | Benzimidazoles | 3.377 | Up |
| H20 | Neoglycyrol | Coumarins | 0.71675 | Up |
| H20 | Suberic acid | Fatty Acyls | 1.8996 | Up |
| H20 | Isosakuranetin | Flavonoids | 0.72603 | Up |
| H20 | Cosmosiin | Flavonoids | 0.77463 | Up |
| H20 | Narcissoside | flavonoids | 0.88521 | Up |
| H20 | Isoscoparin | Flavonoids | 0.89573 | Up |
| H20 | Kaempferol-3-O-rutinoside | flavonoids | 0.90939 | Up |
| H20 | Homoeriodictyol | Flavonoids | 1.4301 | Up |
| H20 | Naringenin | flavonoids | 2.0239 | Up |
| H20 | Genipin-1-O-gentiobioside | Iridoids | 2.1785 | Up |
| H20 | Inosine | Nucleotide and its derivates | 8.5308 | Up |
| H20 | Oxoadipic acid | Organic acids | 1.2591 | Up |
| H20 | 2-Picolinic acid | Organic acids | 1.5483 | Up |
| H20 | N1-Methyl-2-pyridone-5-carboxamide | Pyridines and derivatives | 2.952 | Up |
| H20 | N1-Methyl-4-pyridone-3-carboxamide | Pyridines and derivatives | 3.8908 | Up |

| | | | | |
|---|---|---|---|---|
| H20 | Nandrolone | Steroids and steroid derivatives | 1.2771 | Up |
| H20 | vitamin K2 | Vitamins | 1.2373 | Up |
| H20 | Pyridoxine | Vitamins | 1.4489 | Up |

**Table S5.** Correlation analysis of DEMs in the H01 seedings and physiological parameters (SOD, POD, APX, CAT, $O_2^-$ , $H_2O_2$), SR.

| Traits | Related metabolites | CLASS | Correlation | P-VALUE |
|---|---|---|---|---|
| APX | Tropine acetate | Alkaloids | -0.993 | 0.007 |
| APX | Stevioside | Diterpenoids | -0.996 | 0.004 |
| APX | Homoorientin | Flavonoids | 0.993 | 0.007 |
| APX | Lonicerin | Flavonoids | 0.990 | 0.010 |
| CAT | 5-Hydroxylysine | Amino acids | 0.979 | 0.021 |
| CAT | 6-Methoxymellein | Benzopyrans | 0.971 | 0.029 |
| CAT | Homoorientin | Flavonoids | 0.961 | 0.039 |
| CAT | 6-Deoxyjacareubin | Xanthones | 0.988 | 0.012 |
| CAT | Stevioside | Diterpenoids | -0.988 | 0.012 |
| $H_2O_2$ | N-Acetylarylamine | Benzene and substituted derivatives | -0.977 | 0.023 |
| $H_2O_2$ | Morin | Flavonoids | -0.985 | 0.015 |
| $H_2O_2$ | Atranorin | Phenols | -0.991 | 0.009 |
| $O_2^-$ | Tropine acetate | Alkaloids | -0.968 | 0.032 |
| $O_2^-$ | Stevioside | Diterpenoids | -0.998 | 0.002 |
| $O_2^-$ | Homoorientin | Flavonoids | 0.965 | 0.035 |
| $O_2^-$ | Lonicerin | Flavonoids | 0.981 | 0.019 |
| $O_2^-$ | 6-Deoxyjacareubin | Xanthones | 0.953 | 0.047 |
| POD | Tropine acetate | Alkaloids | -0.959 | 0.041 |
| POD | Stevioside | Diterpenoids | -0.996 | 0.004 |
| POD | 5-Hydroxylysine | Amino acids | 0.953 | 0.047 |
| POD | Homoorientin | Flavonoids | 0.962 | 0.038 |

| | | | | |
|---|---|---|---|---|
| POD | Lonicerin | Flavonoids | 0.971 | 0.029 |
| POD | 6-Deoxyjacareubin | Xanthones | 0.966 | 0.034 |
| SOD | Neolitsine | Alkaloids | -0.966 | 0.034 |
| SOD | N-Acetylarylamine | Benzene and substituted derivatives | -0.997 | 0.003 |
| SOD | Atranorin | Phenols | -0.989 | 0.011 |
| SR | Doronine | Alkaloids | 0.974 | 0.026 |
| SR | 5-Hydroxylysine | Amino acids | -0.958 | 0.042 |
| SR | Bruceine D | Diterpenoids | -0.983 | 0.017 |
| SR | Clovin | Flavonoids | -0.998 | 0.002 |
| SR | Quercetin | Flavonoids | -0.988 | 0.012 |
| SR | Phytic acid | Miscellaneous | -0.956 | 0.044 |
| SR | 2,6-Dimethyl-7-octene-2,3,6-triol | Monoterpenoids | -1.000 | 0.000 |

**Table S6**. Correlation analysis of DEMs in the H20 seedings and physiological parameters (SOD, POD, APX, CAT, $O_2^-$ , $H_2O_2$), SR.

| Traits | Related metabolites | CLASS | Correlation | P-VALUE |
|---|---|---|---|---|
| APX | 1H-Indole-2,3-dione | Indoles and derivatives | -0.994 | 0.006 |
| APX | DL-Benzylsuccinic acid | Phenylpropanoids | -0.961 | 0.039 |
| CAT | 2(3H)-Benzothiazolethione | Benzothiazoles | 0.980 | 0.020 |
| CAT | Arachidonic acid | Fatty Acyls | 0.971 | 0.029 |
| CAT | Indole-3-carboxaldehyde | Indoles and derivatives | 0.992 | 0.008 |
| $H_2O_2$ | 2(3H)-Benzothiazolethione | Benzothiazoles | 0.976 | 0.024 |
| $H_2O_2$ | Forskolin | Diterpenoids | 0.965 | 0.035 |
| $H_2O_2$ | Arachidonic acid | Fatty Acyls | 0.982 | 0.018 |
| $H_2O_2$ | Flavonol | Flavonoids | 0.988 | 0.012 |
| $H_2O_2$ | Clovin | Flavonoids | 0.999 | 0.001 |
| $H_2O_2$ | L-Malic acid | Hydroxy acids and derivatives | 0.957 | 0.043 |
| $H_2O_2$ | Indole-3-carboxaldehyde | Indoles and derivatives | 0.958 | 0.042 |
| $H_2O_2$ | Oleocanthal | Phenols | 0.983 | 0.017 |
| $H_2O_2$ | Alternariol | Phenols | 0.992 | 0.008 |
| $O_2^-$ | 2(3H)-Benzothiazolethione | Benzothiazoles | 0.964 | 0.036 |
| $O_2^-$ | Forskolin | Diterpenoids | 0.976 | 0.024 |
| $O_2^-$ | Arachidonic acid | Fatty Acyls | 0.972 | 0.028 |
| $O_2^-$ | Flavonol | Flavonoids | 0.992 | 0.008 |
| $O_2^-$ | Clovin | Flavonoids | 0.999 | 0.001 |
| $O_2^-$ | L-Malic acid | Hydroxy acids and derivatives | 0.969 | 0.031 |

| | | | | |
|---|---|---|---|---|
| $O_2^-$ | Oleocanthal | Phenols | 0.978 | 0.022 |
| $O_2^-$ | Alternariol | Phenols | 0.990 | 0.010 |
| SOD | beta-Cryptoxanthin | Miscellaneous | -0.955 | 0.045 |
| SOD | Aloeemodin | Anthraquinones | 0.970 | 0.030 |
| SOD | Forskolin | Diterpenoids | 0.994 | 0.006 |
| SOD | Clovin | Flavonoids | 0.956 | 0.044 |
| SOD | Flavonol | Flavonoids | 0.977 | 0.023 |
| SOD | L-Malic acid | Hydroxy acids and derivatives | 0.996 | 0.004 |
| SOD | Dihydrokavain | Phenols | 0.956 | 0.044 |
| SR | Forskolin | Diterpenoids | -1.000 | 0.000 |
| SR | L-Malic acid | Hydroxy acids and derivatives | -1.000 | 0.000 |
| SR | Flavonol | Flavonoids | -0.968 | 0.032 |
| SR | Clovin | Flavonoids | -0.967 | 0.033 |
| SR | Lactupicrin | Sesquiterpenoids | -0.954 | 0.046 |

**Table S7**. Correlation analysis of DEMs in the H01 seedings and photosynthetic parameters and chlorophyll fluorescence parameters

| **Traits** | **Related metabolites** | **CLASS** | **Correlation** | **P-VALUE** |
|---|---|---|---|---|
| SC | Protocatechuic acid | Phenols | 0.9987165 | 0.0012835 |
| Ci | Lusianthridin | Miscellaneous | 0.9931358 | 0.0068642 |
| Ls | Ganoderic acid F | Triterpenoids | 0.9858108 | 0.0141892 |
| SC | Palmitoylethanolamide | Carboximidic acids and derivatives | 0.9838308 | 0.0161692 |
| Ci | Encecalin | Phenols | 0.9830142 | 0.0169858 |
| SC | Doronine | Alkaloids | 0.9689069 | 0.0310931 |
| Ci | 5-Aminovaleric acid | Amino acid and derivatives | 0.9609579 | 0.0390421 |
| PR | 7-Methylxanthine | Nucleotide and its derivates | 0.9602341 | 0.0397659 |
| Ls | Calenduloside E | Triterpenoids | 0.9569427 | 0.0430573 |
| TR | Bruceine D | Diterpenoids | -0.952183 | 0.047817 |
| SC | beta-Cryptoxanthin | Miscellaneous | -0.952507 | 0.0474929 |
| SC | 4-Hydroxyphenyl-2-propionic acid | Phenylpropanoic acids | -0.955208 | 0.0447923 |
| PR | Tropine acetate | Alkaloids | -0.957237 | 0.042763 |
| SC | Allocryptopine | Alkaloids | -0.959333 | 0.0406669 |
| Ci | Suberic acid | Fatty Acyls | -0.963421 | 0.0365787 |
| Ls | Darlingine | Alkaloids | -0.964622 | 0.0353777 |
| SC | 4-Pyridoxic acid | Pyridine derivatives | -0.967344 | 0.0326564 |
| Ls | 6-Aminocaproic acid | Fatty Acyls | -0.967688 | 0.0323121 |
| Ci | Podophyllotoxinone | Lignans | -0.96776 | 0.0322396 |
| SC | Aloeemodin | Anthraquinones | -0.969981 | 0.0300193 |

| | | | | |
|---|---|---|---|---|
| Ls | Loganin | Terpene | -0.972384 | 0.0276162 |
| SC | Bornyl acetate | Monoterpenoids | -0.973867 | 0.0261333 |
| SC | Pterosin D | Sesquiterpenoids | -0.974377 | 0.0256229 |
| TR | Quercetin | flavonoids | -0.979006 | 0.0209937 |
| Ls | Vincamine | Alkaloids | -0.981345 | 0.018655 |
| SC | Ganoderic acid L | Alkaloids | -0.981697 | 0.0183027 |
| Ci | Hypotaurine | Alkaloids | -0.98204 | 0.0179595 |
| TR | 6-Deoxyjacareubin | Xanthones | -0.983355 | 0.016645 |
| Ci | 6-Phosphogluconic acid | Organooxygen compounds | -0.985179 | 0.0148211 |
| Ls | Desoxypeganine | Alkaloids | -0.985809 | 0.0141909 |
| TR | 5-Hydroxylysine | amino acids | -0.986689 | 0.0133107 |
| SC | Phytic acid | Miscellaneous | -0.986706 | 0.013294 |
| SC | Savinin | Lignans | -0.98694 | 0.0130596 |
| TR | 2,6-Dimethyl-7-octene-2,3,6-triol | Monoterpenoids | -0.98864 | 0.0113602 |
| TR | Clovin | Flavonoids | -0.994548 | 0.0054524 |
| SC | Farrerol | flavonoids | -0.995866 | 0.0041338 |
| Ls | Deoxyguanosine | Nucleotide and its derivates | -0.997061 | 0.0029395 |
| Ci | Peonidin-3-glucoside | Flavonoids | -0.998181 | 0.0018188 |
| SC | N-Acetylornithine | Carboxylic acids and derivatives | -0.999679 | 0.000321 |
| ETR | 2-Picolinic acid | Organic acids | 0.9994652 | 0.0005348 |
| NPQ | (-)-Anonaine | Alkaloids | 0.9993958 | 0.0006042 |
| ETR | Isoscoparin | Flavonoids | 0.9992517 | 0.0007483 |
| ETR | Riboflavine | Vitamins | 0.9974411 | 0.0025589 |
| ETR | (-)-3-(3,4-Dihydroxyphenyl)-2-methylalanine | Amino acid and its derivatives | 0.9947657 | 0.0052343 |
| NPQ | N-Feruloyl putrescine | Phenolamides | 0.9942326 | 0.0057674 |

| | | | | |
|---|---|---|---|---|
| qP | Gamma-Tocotrienol | Phenols | 0.9885781 | 0.0114219 |
| Fv | Diosmin | Flavonoids | 0.9883687 | 0.0116313 |
| ETR | Delta-Nonalactone | Miscellaneous | 0.9850884 | 0.0149116 |
| Fv | Hypotaurine | Alkaloids | 0.9841726 | 0.0158274 |
| ETR | Wedelolactone | Coumarins | 0.9824214 | 0.0175786 |
| Fv | Isosakuranetin | Flavonoids | 0.982402 | 0.017598 |
| qP | (R)-2-Hydroxy-2H-1,4-benzoxazin-3(4H)-one | Benzoxazines | 0.9821882 | 0.0178118 |
| Fv | Podophyllotoxinone | Lignans | 0.9804165 | 0.0195835 |
| ETR | Convallatoxin | Steroids | 0.9730055 | 0.0269945 |
| ETR | 7-Methylguanine | Imidazopyrimidines | 0.9715945 | 0.0284055 |
| NPQ | 7-Methylxanthine | Nucleotide and its derivates | 0.9666979 | 0.0333021 |
| NPQ | Homoorientin | flavonoids | 0.964492 | 0.035508 |
| qP | Gentioflavin | Alkaloids | 0.9629963 | 0.0370037 |
| NPQ | Eicosadienoic acid | Fatty Acyls | 0.9590586 | 0.0409414 |
| qP | Formylanthranilic acid | Benzene and substituted derivatives | 0.9582863 | 0.0417137 |
| qP | Wedelolactone | Coumarins | 0.9565538 | 0.0434462 |
| Fv | 7-Methylguanine | Imidazopyrimidines | 0.955991 | 0.044009 |
| NPQ | 6-Methoxymellein | Benzopyrans | 0.9559038 | 0.0440962 |
| Fv | Peonidin-3-glucoside | Flavonoids | 0.9541521 | 0.044009 |
| NPQ | Cathinone | Alkaloids | 0.9525628 | 0.0474372 |
| Fv | Quercetin-3-O-sophoroside | Flavonoids | 0.9516023 | 0.0447388 |
| Fv | 4-Hydroxybenzaldehyde | Phenols | 0.9512 | 0.0278759 |
| Fv | Octadecanamide | Fatty Acyls | -0.955261 | 0.0447388 |
| Fv | Oleic acid | Fatty Acyls | -0.957189 | 0.0428112 |
| Fv | Moracin C | Phenols | -0.961353 | 0.0386474 |

| | | | | |
|---|---|---|---|---|
| Fv | Amabiline | Alkaloids | -0.965745 | 0.0342551 |
| Fv | Lusianthridin | Miscellaneous | -0.965897 | 0.0341028 |
| Fv | 4-Hydroxyphenylacetylglutamic acid | Organic acids | -0.972124 | 0.0278759 |
| Fv | Desmethylxanthohumol | Chalcones | -0.972869 | 0.0271311 |
| Fv | Miltirone | Diterpenoids | -0.980015 | 0.0199849 |
| Fv | Mannose 6-phosphate | Organooxygen compounds | -0.981547 | 0.0184528 |
| Fv | Oleamide | Fatty Acyls | -0.982219 | 0.0177807 |
| Fv | Artemisinin | Sesquiterpenoids | -0.982965 | 0.0170345 |
| Fv | Glucose 1-phosphate | Organooxygen compounds | -0.983967 | 0.0160333 |
| Fv | Palmitic acid | Lipids | -0.995186 | 0.0048137 |
| Fv | Chrysoeriol 7-apiosylglucoside | Flavonoids | -0.997284 | 0.0027158 |

**Table S8**. Correlation analysis of DEMs in the H20 seedings and photosynthetic parameters and chlorophyll fluorescence parameters

| **Traits** | **Related metabolites** | **CLASS** | **Correlation** | **P-VALUE** |
|---|---|---|---|---|
| TR | L-Malic acid | Hydroxy acids and derivatives | -0.999519 | 0.0160103 |
| TR | Forskolin | Diterpenoids | -0.999326 | 0.0103864 |
| PR | Clovin | Flavonoids | -0.99534 | 0.0357425 |
| PR | Forskolin | Diterpenoids | -0.989614 | 0.0103864 |
| PR | L-Malic acid | Hydroxy acids and derivatives | -0.98399 | 0.0160103 |
| PR | Flavonol | Flavonoids | -0.981638 | 0.0222846 |
| SC | Aloeemodin | Anthraquinones | -0.977422 | 0.0225778 |
| PR | Alternariol | Phenols | -0.969785 | 0.0302151 |
| TR | Flavonol | Flavonoids | -0.964555 | 0.0222846 |
| TR | Clovin | Flavonoids | -0.964258 | 0.0357425 |
| TR | Lactupicrin | Sesquiterpenoids | -0.9559 | 0.0441002 |
| PR | Lactupicrin | Sesquiterpenoids | -0.953419 | 0.0441002 |
| PR | Oleocanthal | Phenols | -0.951556 | 0.0484437 |
| Ls | Homoeriodictyol | Flavonoids | 0.9528405 | 0.0471595 |
| Ci | Forskolin | Diterpenoids | 0.9634418 | 0.0365582 |
| Ls | (R)-2-Hydroxy-2H-1,4-benzoxazin-3(4H)-one | Benzoxazines | 0.9643506 | 0.0356494 |
| Ci | L-Malic acid | Hydroxy acids and derivatives | 0.967085 | 0.000481 |
| Ci | Aloeemodin | Anthraquinones | 0.9760921 | 0.0225778 |
| Ci | Flavonol | Flavonoids | 0.9777154 | 0.0354452 |

| | | | | |
|---|---|---|---|---|
| SC | Acacetin | Flavonoids | 0.9878377 | 0.0121623 |
| Ci | Dihydrokavain | Phenols | 0.9880142 | 0.0119858 |
| SC | beta-Cryptoxanthin | Miscellaneous | 0.9998106 | 0.0001894 |
| Fv | Clovin | Flavonoids | -0.998456 | 0.0357425 |
| ETR | L-Malic acid | Hydroxy acids and derivatives | -0.997064 | 0.0336588 |
| ETR | Forskolin | Diterpenoids | -0.995741 | 0.0251961 |
| NPQ | Ganoderic acid F | Triterpenoids | -0.98064 | 0.0193599 |
| NPQ | Oxychelerythrine | Alkaloids | -0.975346 | 0.0246538 |
| qP | 3-Methylxanthine | Nucleotide and its derivates | -0.974833 | 0.025167 |
| Fv | Forskolin | Diterpenoids | -0.974804 | 0.0251961 |
| Fv | Flavonol | Flavonoids | -0.974314 | 0.0256861 |
| Fv | Alternariol | Phenols | -0.97293 | 0.0270704 |
| Fv | L-Malic acid | Hydroxy acids and derivatives | -0.966341 | 0.0336588 |
| Fv | Arachidonic acid | Fatty Acyls | -0.963295 | 0.0367045 |
| ETR | Lactupicrin | Sesquiterpenoids | -0.960706 | 0.0392936 |
| Fv | 2(3H)-Benzothiazolethione | Benzothiazoles | -0.958908 | 0.0410919 |
| Fv | Oleocanthal | Phenols | -0.958138 | 0.0418615 |
| Fv | Lactupicrin | Sesquiterpenoids | -0.950291 | 0.0015441 |
| ETR | Clovin | Flavonoids | -0.950051 | 0.0499491 |
| ETR | beta-Cryptoxanthin | Miscellaneous | 0.9594644 | 0.0405356 |
| qP | Acacetin | Flavonoids | 0.9616663 | 0.0383337 |
| qP | beta-Cryptoxanthin | Miscellaneous | 0.9653788 | 0.0346212 |
| NPQ | Flavonol | Flavonoids | 0.9673133 | 0.0326867 |
| NPQ | Dihydrokavain | Phenols | 0.9984562 | 0.0015438 |
