# Supplementary figures and images for: Metabolomic analyses reveal that graphene oxide alleviates nicosulfuron toxicity in sweet corn
Source: Front Plant Sci. 2025 Feb 25;16:1529598. doi: 10.3389/fpls.2025.1529598 (PMC11893866; doi:10.3389/fpls.2025.1529598)

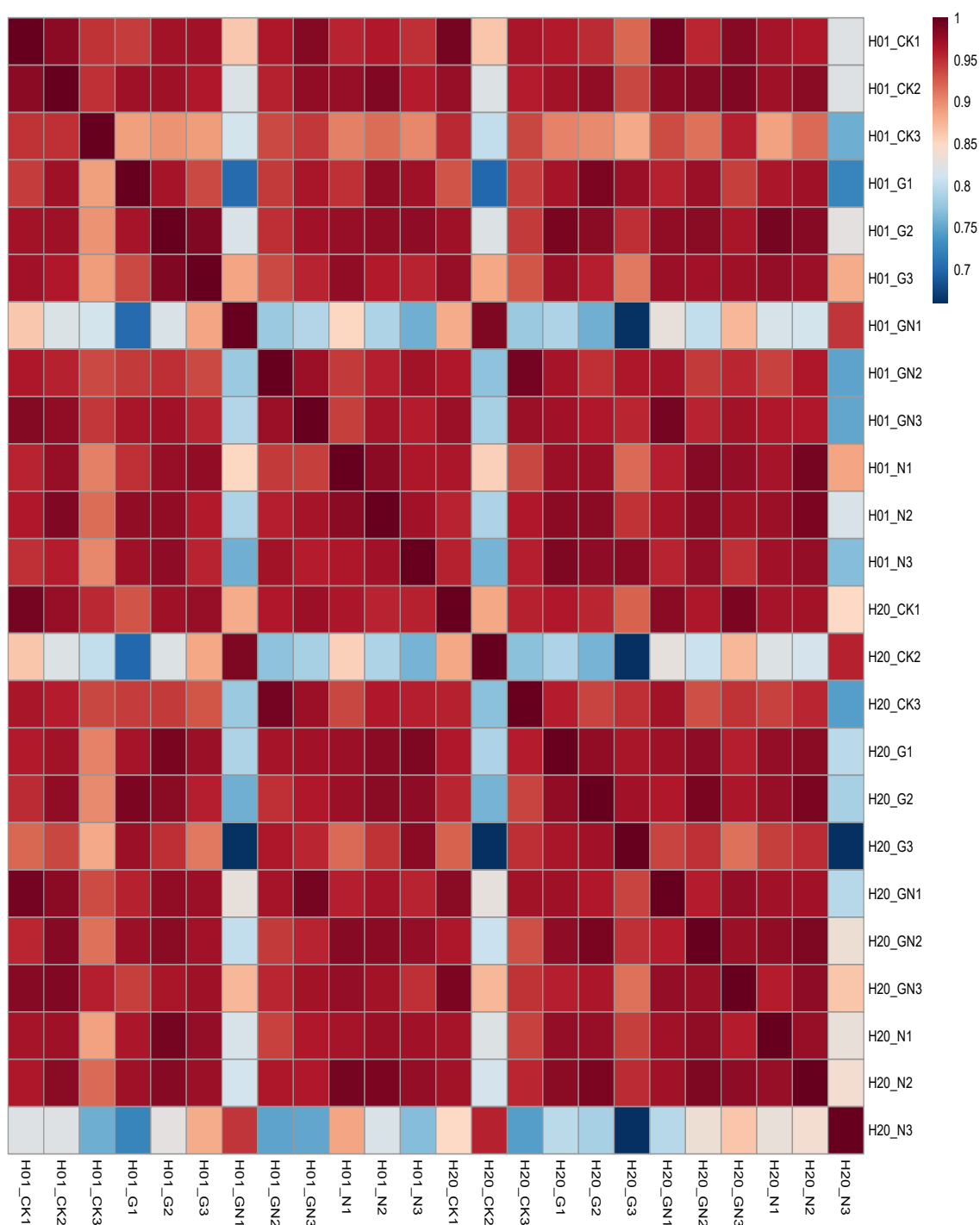

**Fig. S1.** Correlation diagram of all the samples.

Supplement: Supplementary file 2 [file Image1.pdf]

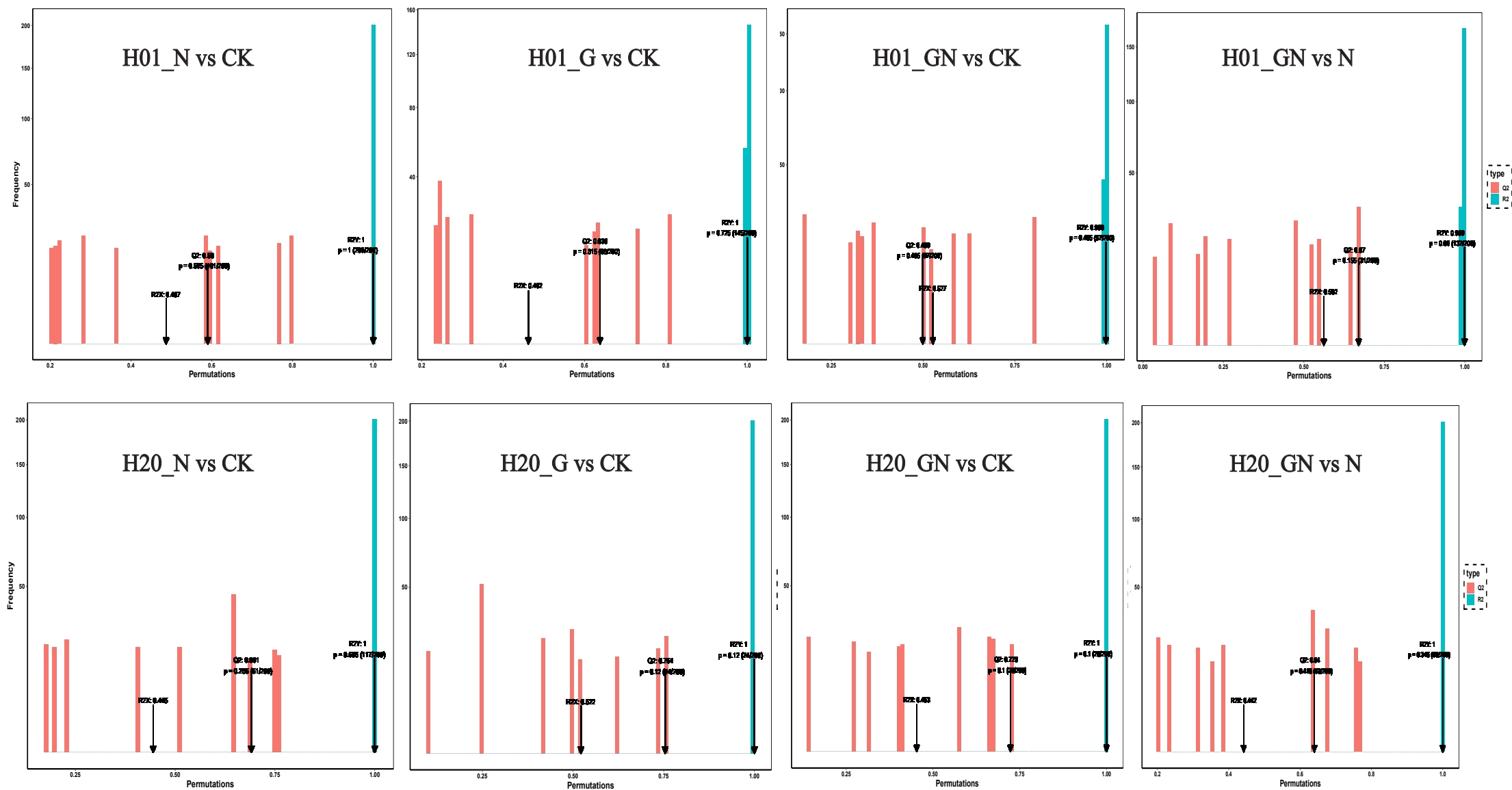

Fig. S2. OPLS-DA analysis of different group.

Supplement: Supplementary file 3 [file Image2.pdf]

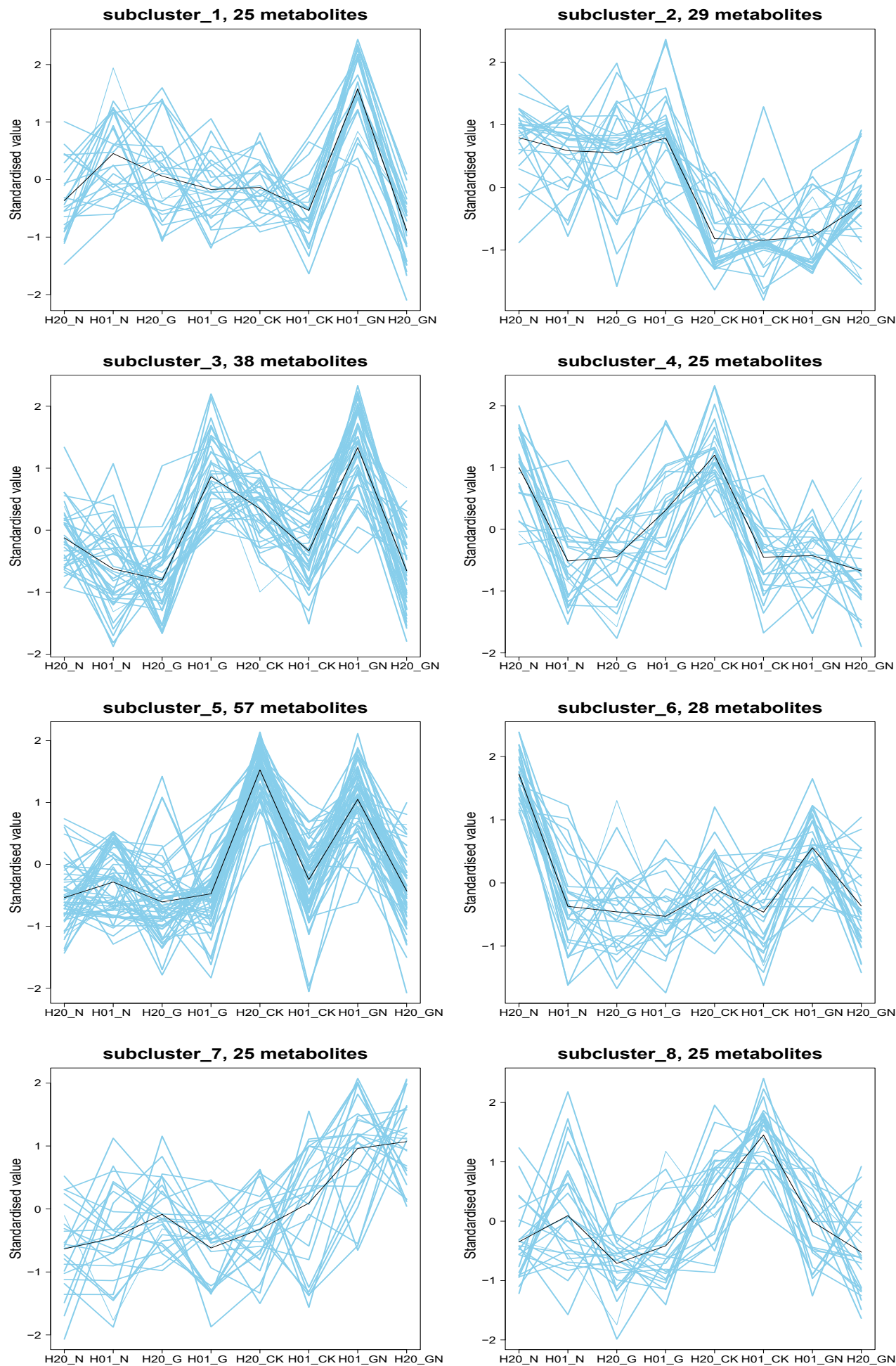

Fig. S3. K-means analysis of DEMs.

Supplement: Supplementary file 4 [file Image3.pdf]

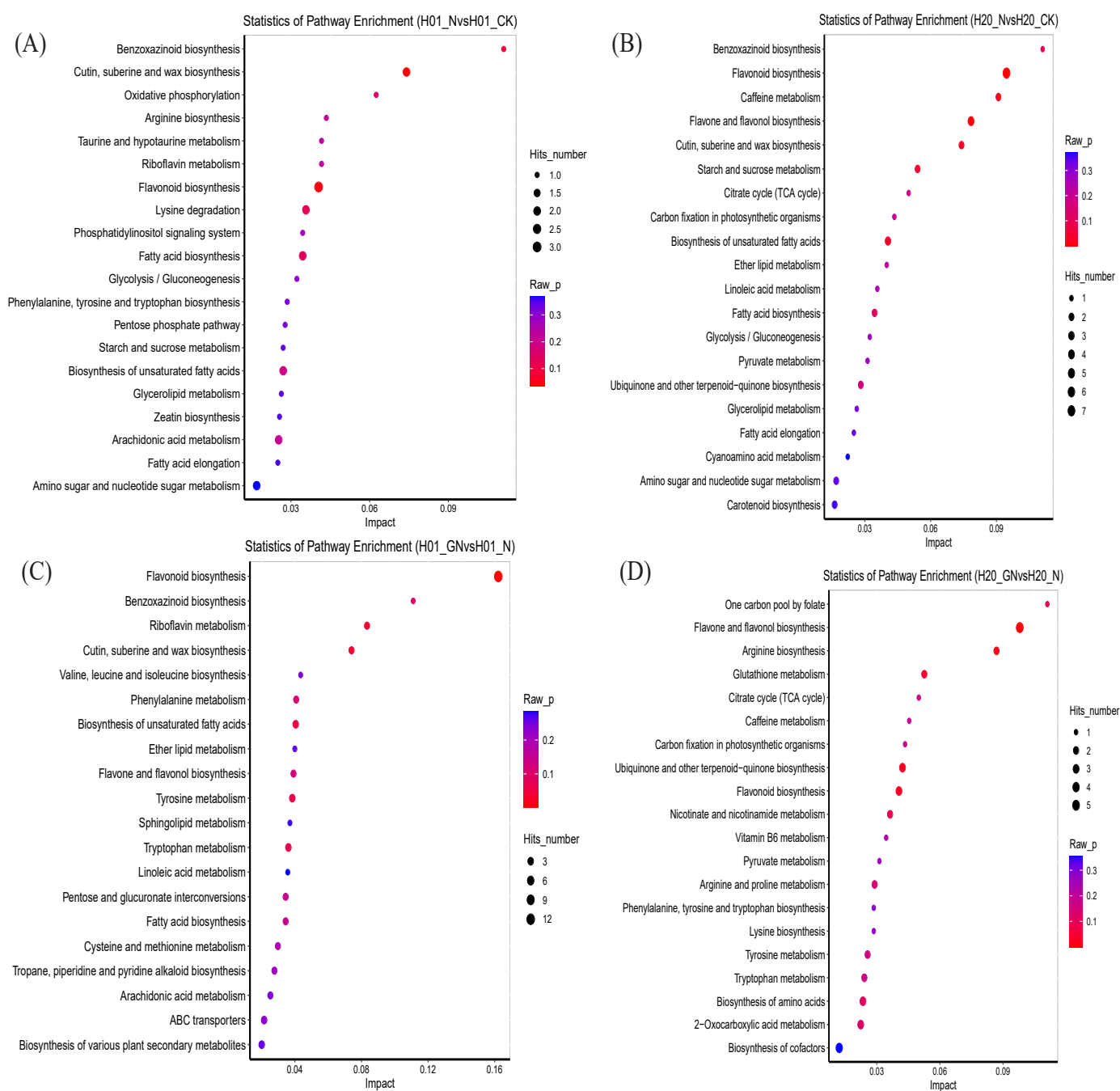

**Fig. S10.** KEGG enrichment of DEMs. Pathway enrichment of H01\_N vs CK (A) and H20\_N vs CK (B), H01\_GN vs N (C) and H20\_GN vs N (D).

Supplement: Supplementary file 11 [file Image10.pdf]
