# Supplementary material for: Metabolomic analyses reveal that graphene oxide alleviates nicosulfuron toxicity in sweet corn
Source: Front Plant Sci. 2025 Feb 25;16:1529598. doi: 10.3389/fpls.2025.1529598 (PMC11893866; doi:10.3389/fpls.2025.1529598)
Supplement: Supplementary file 6 [file Image5.pdf]

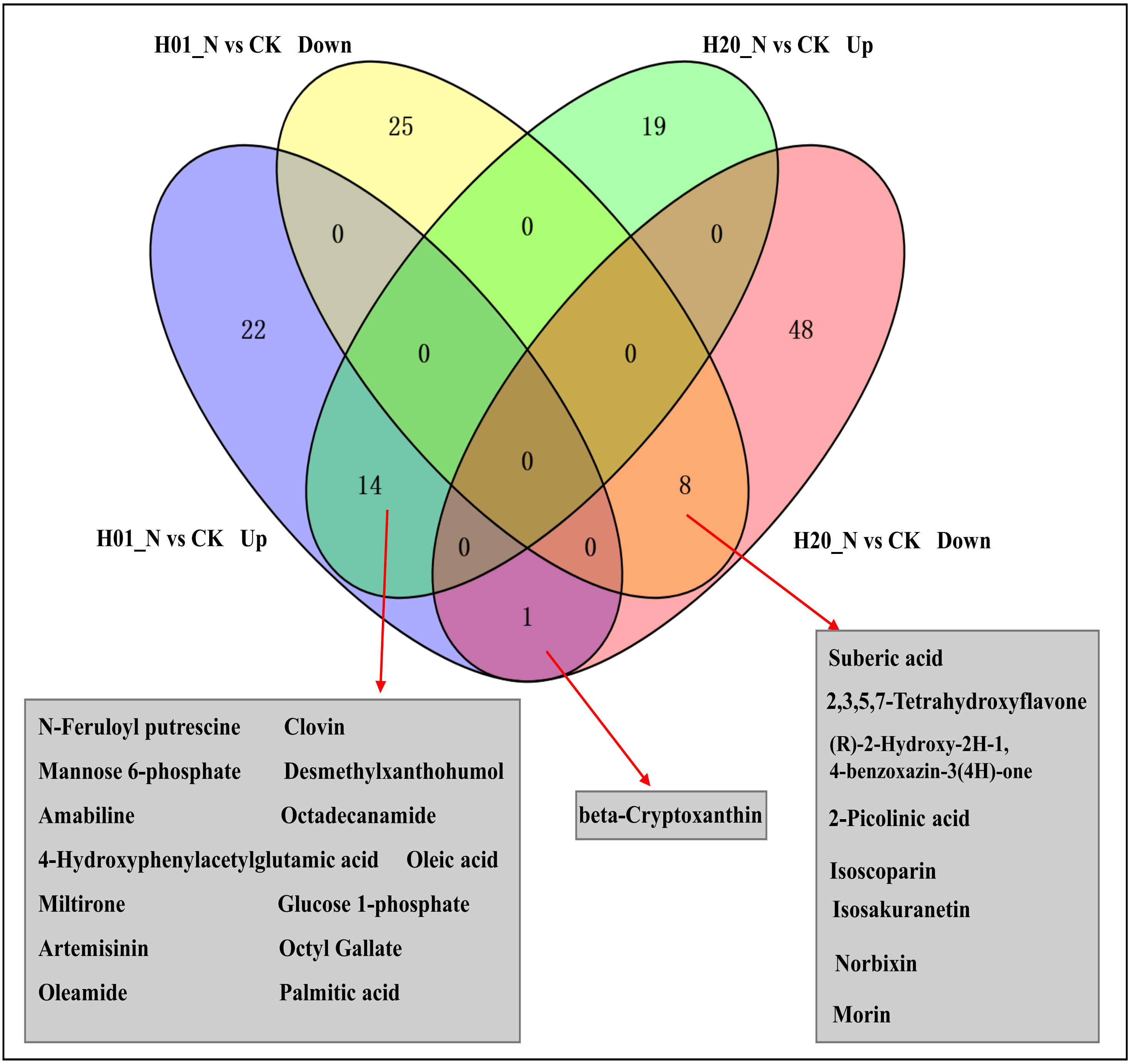


**Fig. S5**. Venn analysis of differentially accumulated metabolites in the H01 and H20 seedlings exposed to NIF stress. Up, upregulated metabolites; down, downregulated metabolites (N vs CK).
