# Supplementary material for: Metabolomic analyses reveal that graphene oxide alleviates nicosulfuron toxicity in sweet corn
Source: Front Plant Sci. 2025 Feb 25;16:1529598. doi: 10.3389/fpls.2025.1529598 (PMC11893866; doi:10.3389/fpls.2025.1529598)
Supplement: Supplementary file 7 [file Image6.pdf]

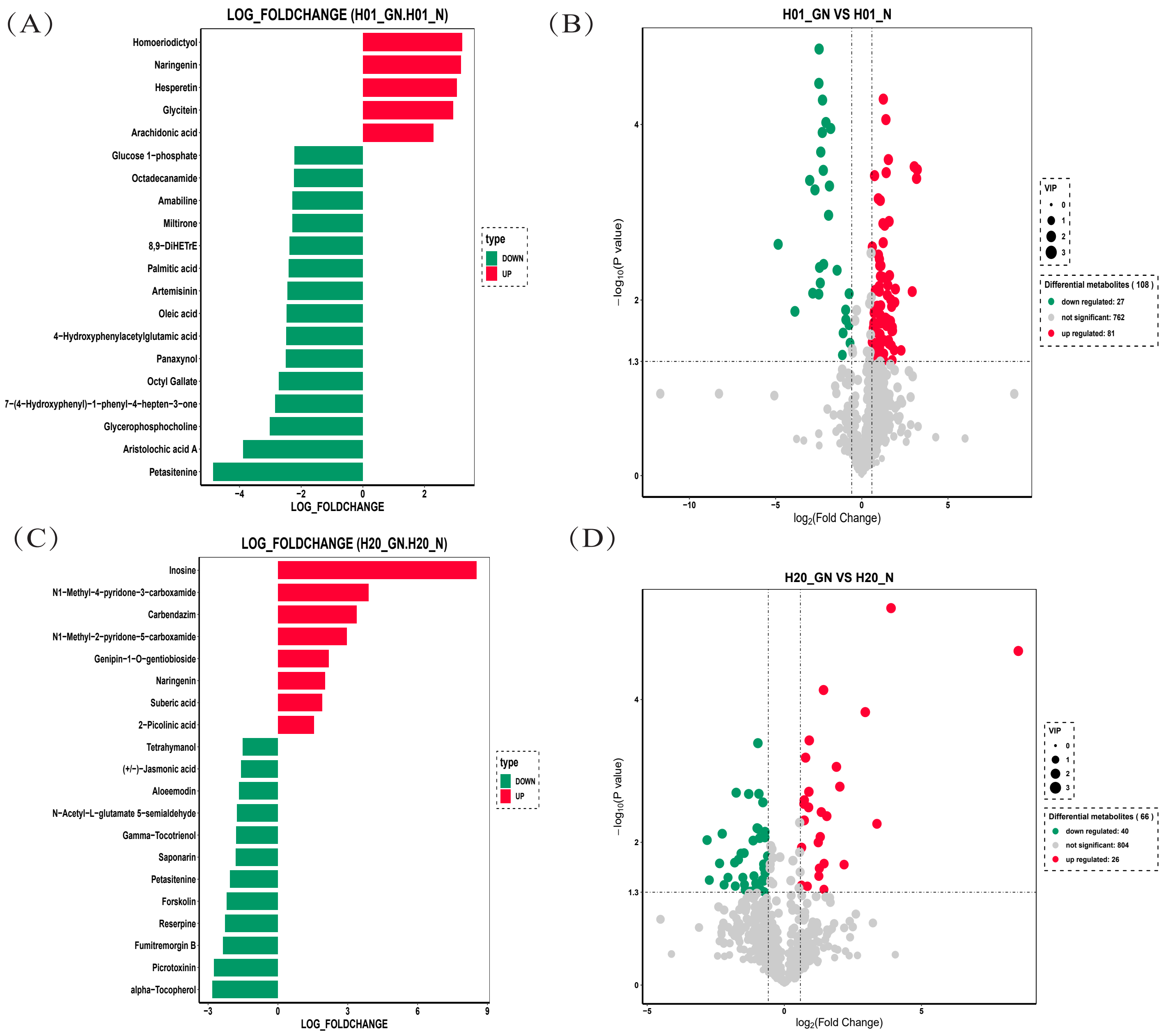

Fig. S6. GO-induced differentially expressed metabolites in the NIF-treated H01 inbred line (A, B) and H20 inbred line (C, D).
