# Supplementary material for: Metabolomic analyses reveal that graphene oxide alleviates nicosulfuron toxicity in sweet corn
Source: Front Plant Sci. 2025 Feb 25;16:1529598. doi: 10.3389/fpls.2025.1529598 (PMC11893866; doi:10.3389/fpls.2025.1529598)
Supplement: Supplementary file 8 [file Image7.pdf]

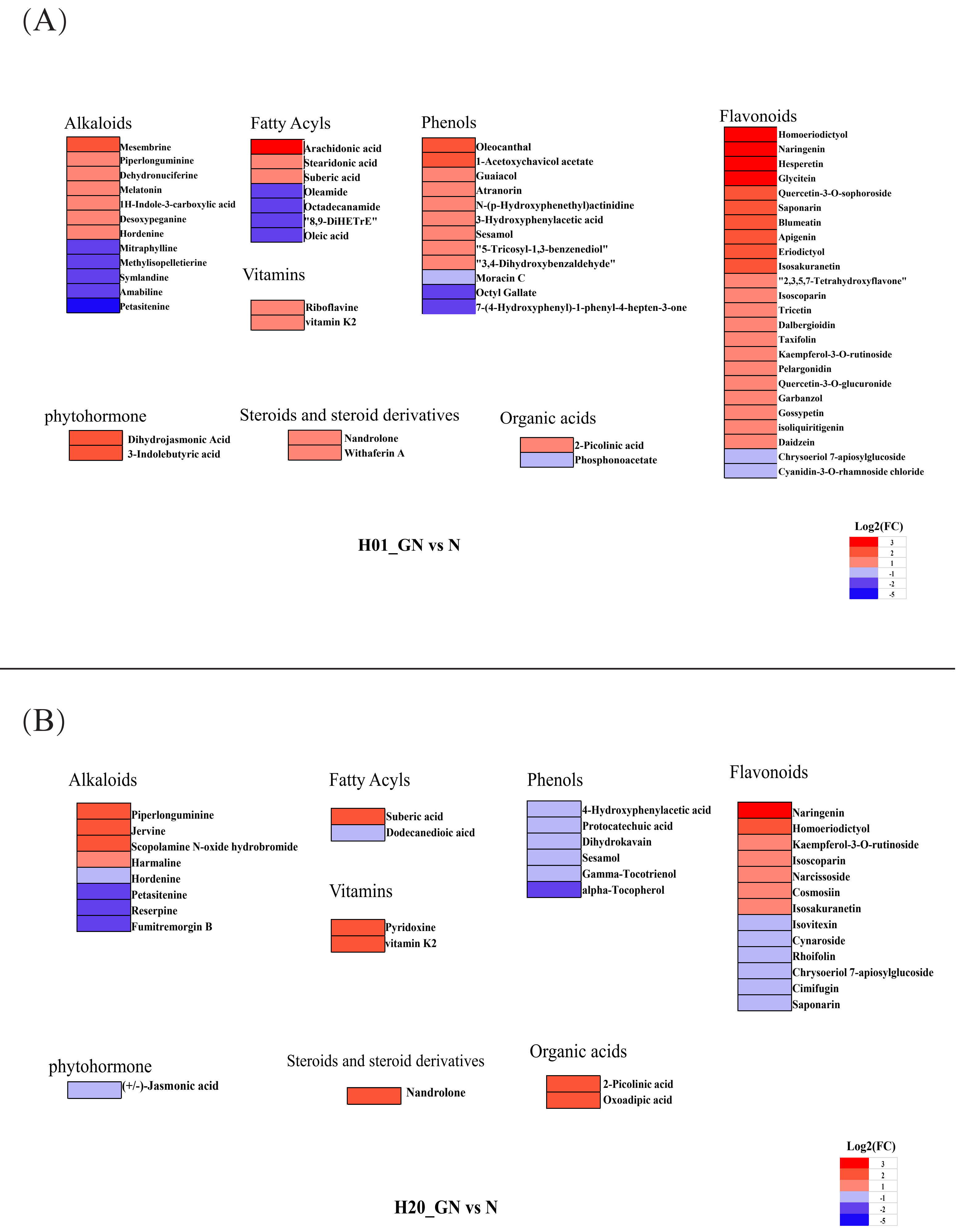

Fig.S7. Comparative analyses of DEMs between GN and NIF stress. (A) Heat map of significant DEMs in the H01 seedings (B) Heat map of significant DEMs in the H20 seedings.
