# Supplementary material for: Metabolomic analyses reveal that graphene oxide alleviates nicosulfuron toxicity in sweet corn
Source: Front Plant Sci. 2025 Feb 25;16:1529598. doi: 10.3389/fpls.2025.1529598 (PMC11893866; doi:10.3389/fpls.2025.1529598)
Supplement: Supplementary file 9 [file Image8.pdf]

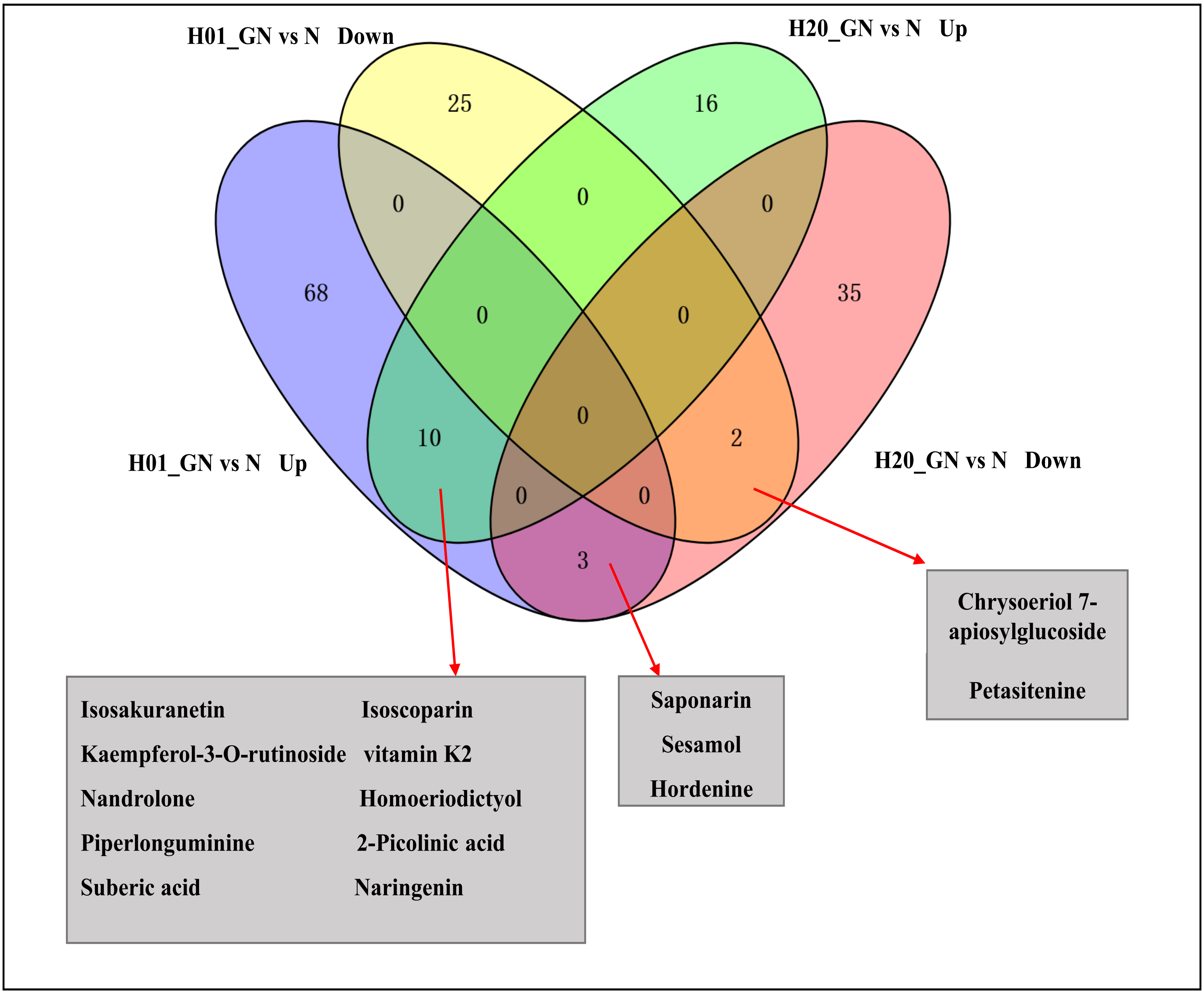

Fig. S8. Venn analysis of differentially accumulated metabolites in the H01 and H20 of NIF-treated seedlings exposed to GO (GN vs N).
