# Supplementary material for: Metabolomic analyses reveal that graphene oxide alleviates nicosulfuron toxicity in sweet corn
Source: Front Plant Sci. 2025 Feb 25;16:1529598. doi: 10.3389/fpls.2025.1529598 (PMC11893866; doi:10.3389/fpls.2025.1529598)
Supplement: Supplementary file 10 [file Image9.pdf]

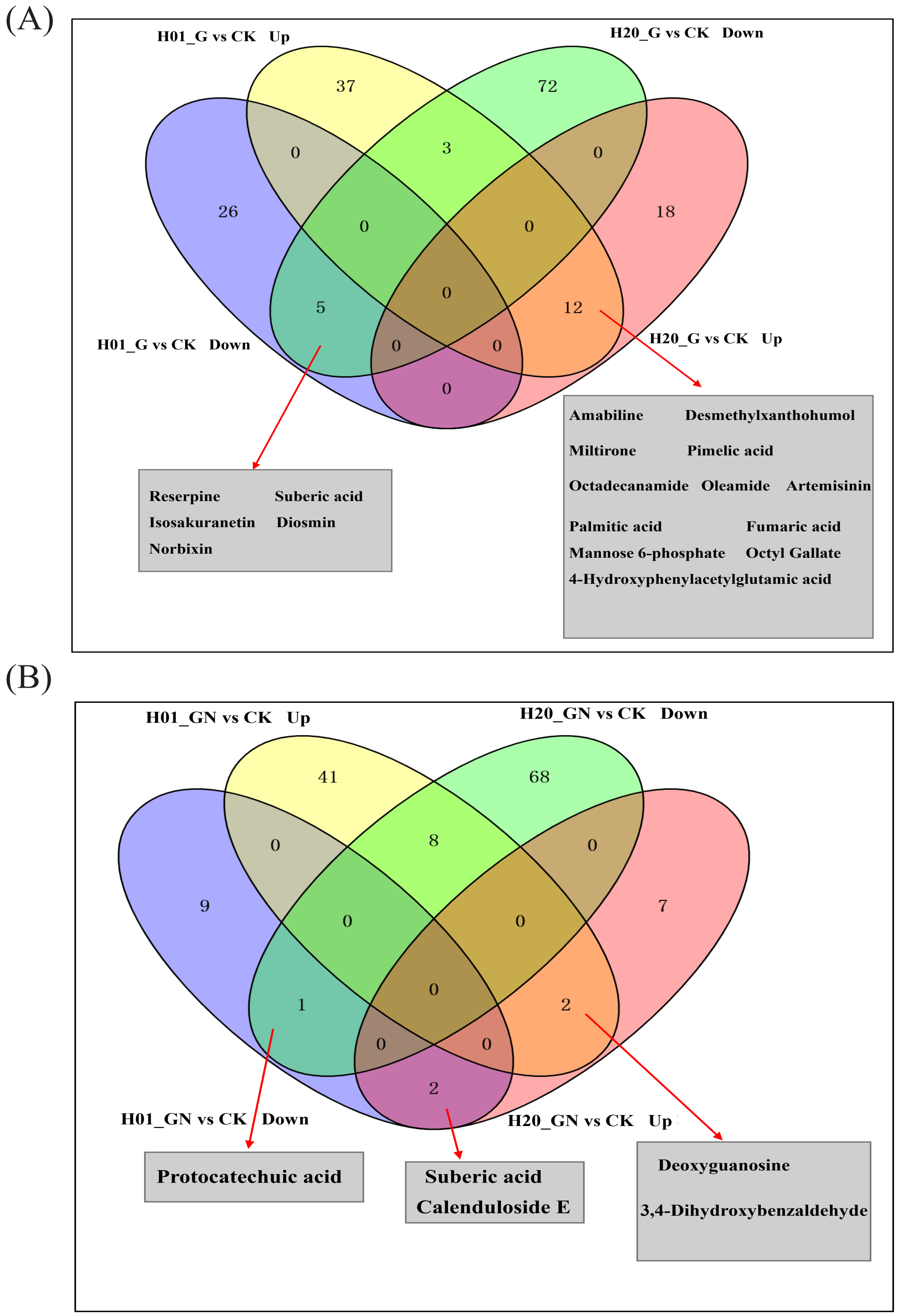

Fig. S9. Venn analysis of DEMs among different group. (A) Venn analysis of DEMs in the H01 and H20 of seedlings under GO treatment (G vs CK). (B) Venn analysis of DEMs in the H01 and H20 seedlings exposed to GN (GN vs CK).
